# Supplementary material for: AI-guided few-shot inverse design of HDP-mimicking polymers against drug-resistant bacteria
Source: Nat Commun. 2024 Jul 26;15:6288. doi: 10.1038/s41467-024-50533-4 (PMC11282099; doi:10.1038/s41467-024-50533-4)
Supplement: Supplementary file 1 — Supplementary Information [file 41467_2024_50533_MOESM1_ESM.pdf]

Supplementary Information

AI-guided Few-shot Inverse Design of HDP-Mimicking Polymers against Drug-Resistant Bacteria

Tianyu Wu[1†], Min Zhou[2†], Jingcheng Zou[3], Qi Chen[3], Feng Qian[1], Jürgen Kurths[4,5], Runhui Liu[2,3*], Yang Tang[1*]

[1]Key Laboratory of Smart Manufacturing in Energy Chemical Process, East China University of Science and Technology, Shanghai, 200237, China.
[2]State Key Laboratory of Bioreactor Engineering, East China University of Science and Technology, Shanghai, 200237, China.
[3]Shanghai Frontiers Science Center of Optogenetic Techniques for Cell Metabolism, Frontiers Science Center for Materiobiology and Dynamic Chemistry, Key Laboratory for Ultrafine Materials of Ministry of Education, Research Center for Biomedical Materials of Ministry of Education, School of Materials Science and Engineering, East China University of Science and Technology, Shanghai, 200237, China.
[4]Potsdam Institute for Climate Impact Research (PIK), Potsdam, 14473, Germany.
[5]Institut für Physik, Humboldt-Universität zu Berlin, Berlin, 10115, Germany.

*Corresponding author(s). E-mail(s): rliu@ecust.edu.cn; yangtang@ecust.edu.cn;
Contributing authors: tianyuwu813@gmail.com; minzhou072@163.com; 13757533643@163.com; chenqi0526@foxmail.com; fqian@ecust.edu.cn; juergen.kurths@pik-potsdam.de;
†These authors contributed equally to this work.

List of Figures

S1 Supplementary Fig 1 1
S2 Supplementary Fig 2 2
S3 Supplementary Fig 3 3
S4 Supplementary Fig 4 4
S5 Supplementary Fig 5 5
S6 Supplementary Fig 6 6
S7 Supplementary Fig 7 7
S8 Supplementary Fig 8 8
S9 Supplementary Fig 9 9
S10 Supplementary Fig 10 10
S11 Supplementary Fig 11 11
S12 Supplementary Fig 12 11
S13 Supplementary Fig 13 13
S14 Supplementary Fig 14 14
S15 Supplementary Fig 15 14
S16 Supplementary Fig 16 15
S17 Supplementary Fig 17 16
S18 Supplementary Fig 18 17
S19 Supplementary Fig 19 17
S20 Supplementary Fig 20 18
S21 Supplementary Fig 21 18
S22 Supplementary Fig 22 19
S23 Supplementary Fig 23 20
S24 Supplementary Fig 24 21
S25 Supplementary Fig 25 22
S26 Supplementary Fig 26 23
S27 Supplementary Fig 27 23
S28 Supplementary Fig 28 24
S29 Supplementary Fig 29 24
S30 Supplementary Fig 30 25
S31 Supplementary Fig 31 26
S32 Supplementary Fig 32 27
S33 Supplementary Fig 33 28
S34 Supplementary Fig 34 29
S35 Supplementary Fig 35 30
S36 Supplementary Fig 36 31
S37 Supplementary Fig 37 32
S38 Supplementary Fig 38 33
S39 Supplementary Fig 39 34

List of Figures

40 Supplementary Fig 40 35
41 Supplementary Fig 41 36
42 Supplementary Fig 42 36
43 Supplementary Fig 43 37
44 Supplementary Fig 44 37
45 Supplementary Fig 45 38
46 Supplementary Fig 46 38
47 Supplementary Fig 47 39
48 Supplementary Fig 48 39
49 Supplementary Fig 49 40
50 Supplementary Fig 50 40
51 Supplementary Fig 51 41
52 Supplementary Fig 52 42
53 Supplementary Fig 53 43
54 Supplementary Fig 54 44
55 Supplementary Fig 55 44
56 Supplementary Fig 56 45
57 Supplementary Fig 57 45
58 Supplementary Fig 58 46
59 Supplementary Fig 59 47
60 Supplementary Fig 60 48
61 Supplementary Fig 61 49
62 Supplementary Fig 62 50
63 Supplementary Fig 63 51
64 Supplementary Fig 64 52
65 Supplementary Fig 65 53
66 Supplementary Fig 66 54
67 Supplementary Fig 67 55
68 Supplementary Fig 68 55
69 Supplementary Fig 69 56
70 Supplementary Fig 70 56
71 Supplementary Fig 71 57
72 Supplementary Fig 72 57
73 Supplementary Fig 73 58
74 Supplementary Fig 74 58

List of Tables

1. Supplementary Table 1 59
2. Supplementary Table 2 60
3. Supplementary Table 3 61
4. Supplementary Table 4 62
5. Supplementary Table 5 63
6. Supplementary Table 6 64
7. Supplementary Table 7 65
8. Supplementary Table 8 66
9. Supplementary Table 9 67
10. Supplementary Table 10 68
11. Supplementary Table 11 69
12. Supplementary Table 12 70
13. Supplementary Table 13 71
14. Supplementary Table 14 72
15. Supplementary Table 15 73
16. Supplementary Table 16 74
17. Supplementary Table 17 75
18. Supplementary Table 18 76
19. Supplementary Table 19 77
20. Supplementary Table 20 78

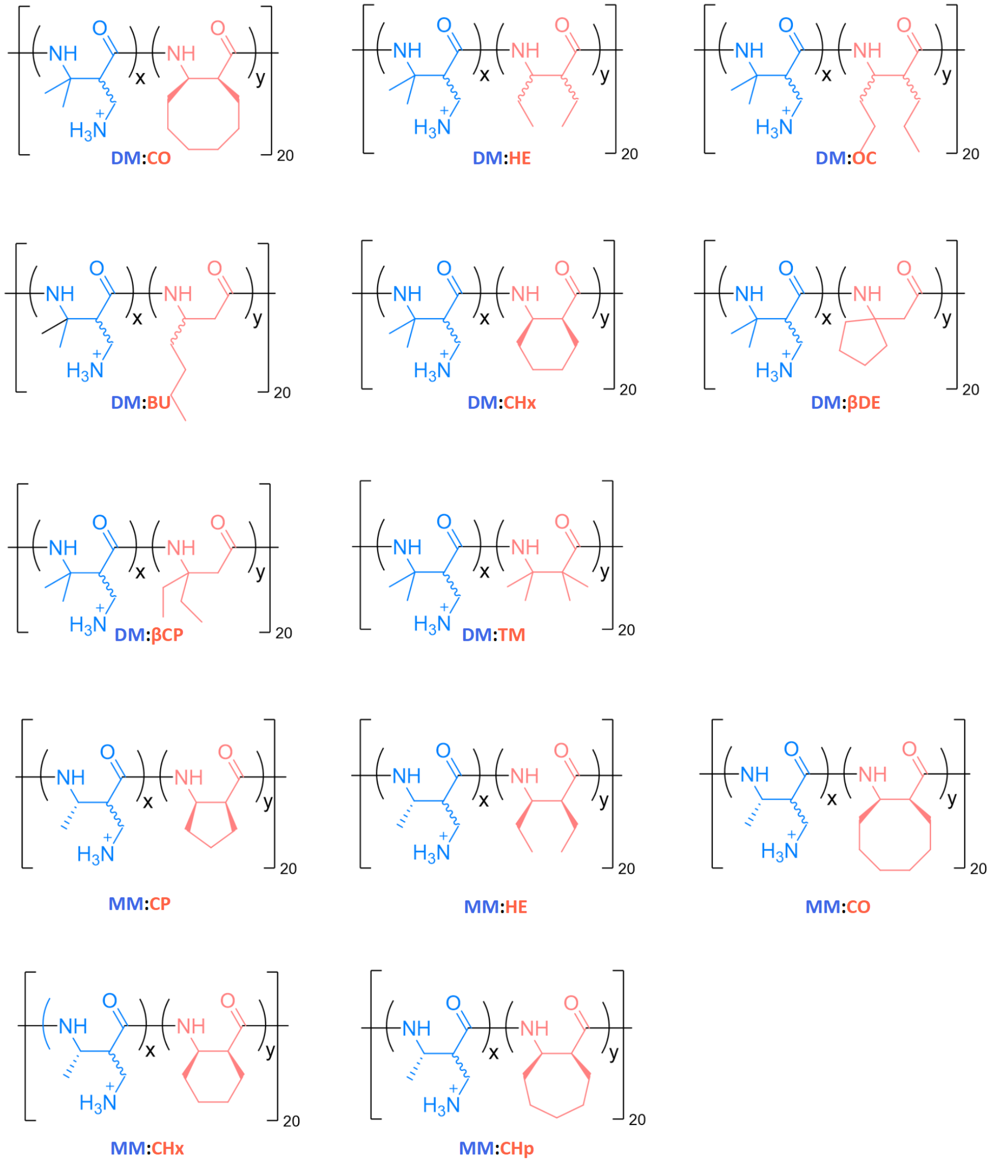

Supplementary Fig. 1 All cationic-hydrophobic amphiphilic β-amino acid polymers are reported in precedent literatures. Structures in blue and red are cationic and hydrophobic amino acid resides (subunits), respectively. x, y are their composition ratio; “20” means the total length of the polymer containing around 20 amino acid residues.

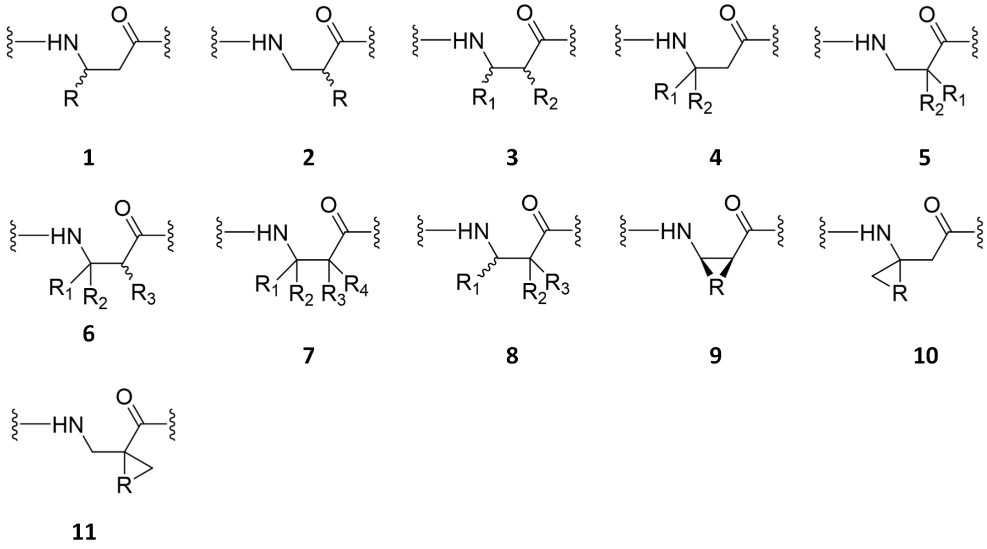

Supplementary Fig. 2 Created scaffolds according to the different position of side chain substituents and cyclic/non-cyclic substitution pattern of β-amino acid polymer. "R" is the substitution point of a scaffold. "R_1, R_2, R_3, R_4" means that more than one substitution point should be decorated.

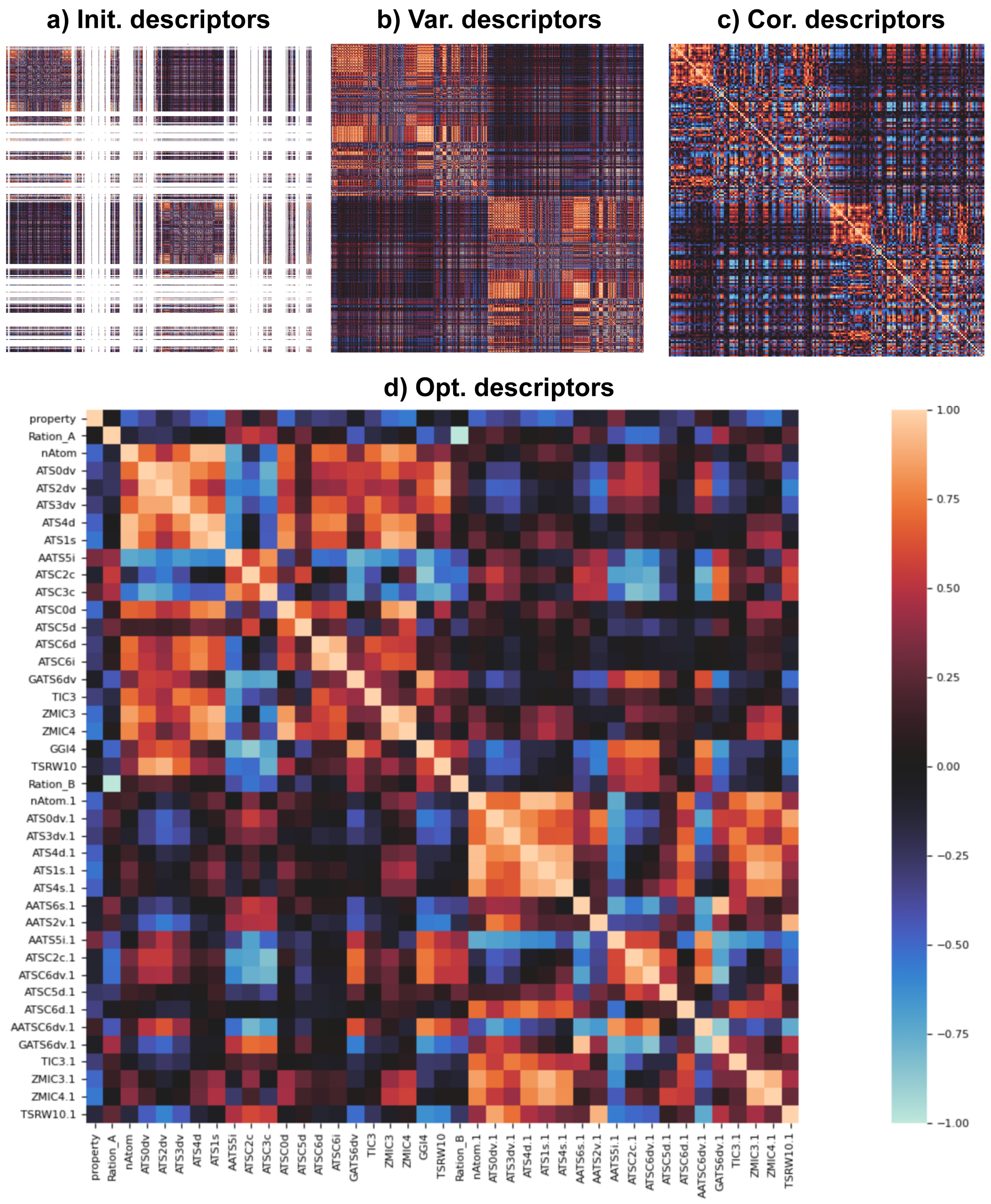

Supplementary Fig. 3 Spearman rank correlation matrices in the downselection process for MIC$_{S.\ aureus}$. a) Init. descriptors, b) Var. descriptors, c) Cor. descriptors and d) Opt. descriptors. Values close to 0 mean low correlations, while values near 1 or -1 mean high correlations.

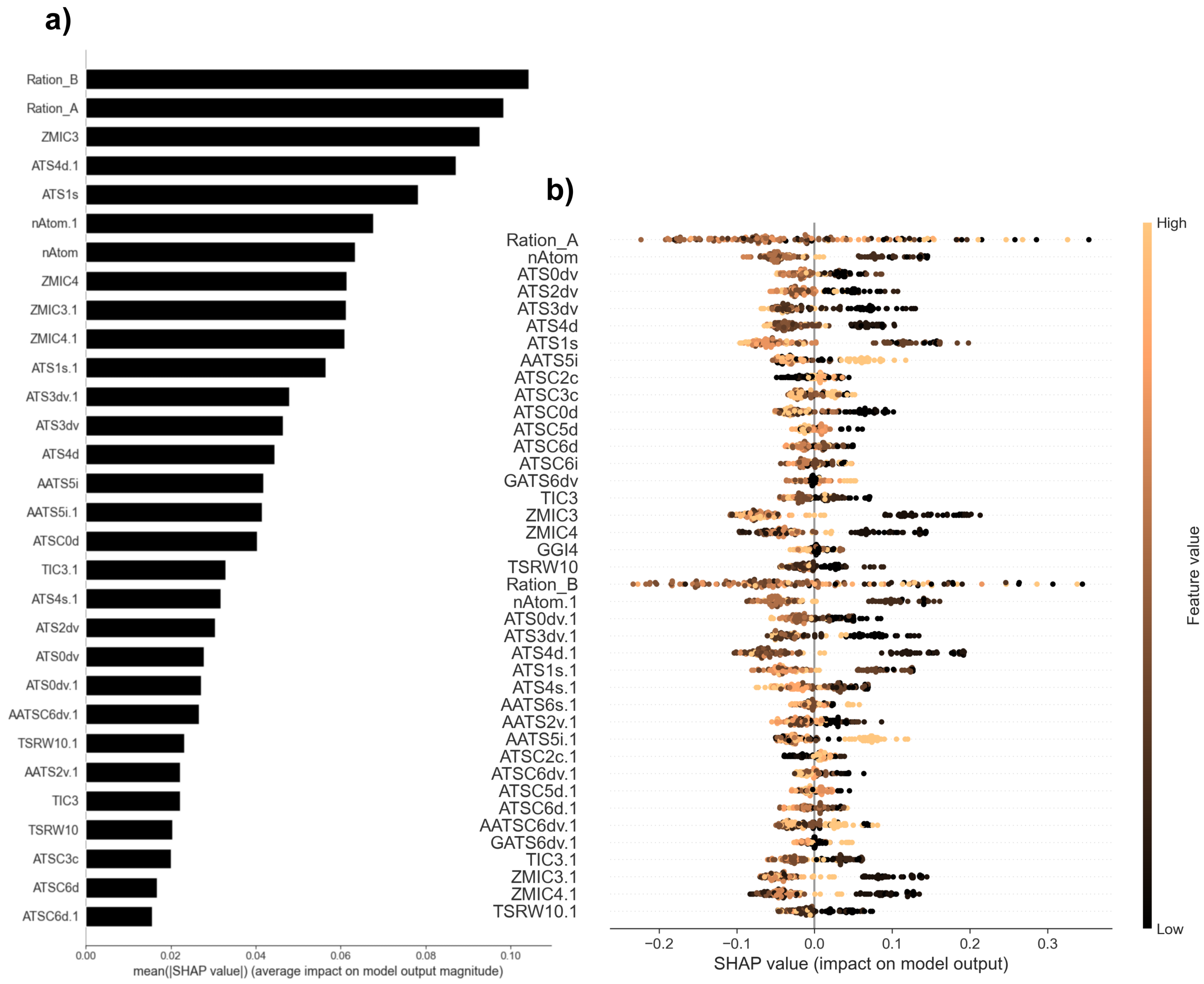

Supplementary Fig. 4 Importance ranking of descriptors and Shapley additive explanations (SHAP) analysis for MIC$_{S.\ aureus}$. a) Importance ranking of descriptors (top 30 descriptors are shown) for property of MIC$_{S.\ aureus}$ chosen in the random forest regression process with using recursive feature elimination (RFE). b) Shapley additive explanations (SHAP) analysis of the optimized descriptors. Note that descriptors which are decorated with ".1" means the descriptors for the second subunit.

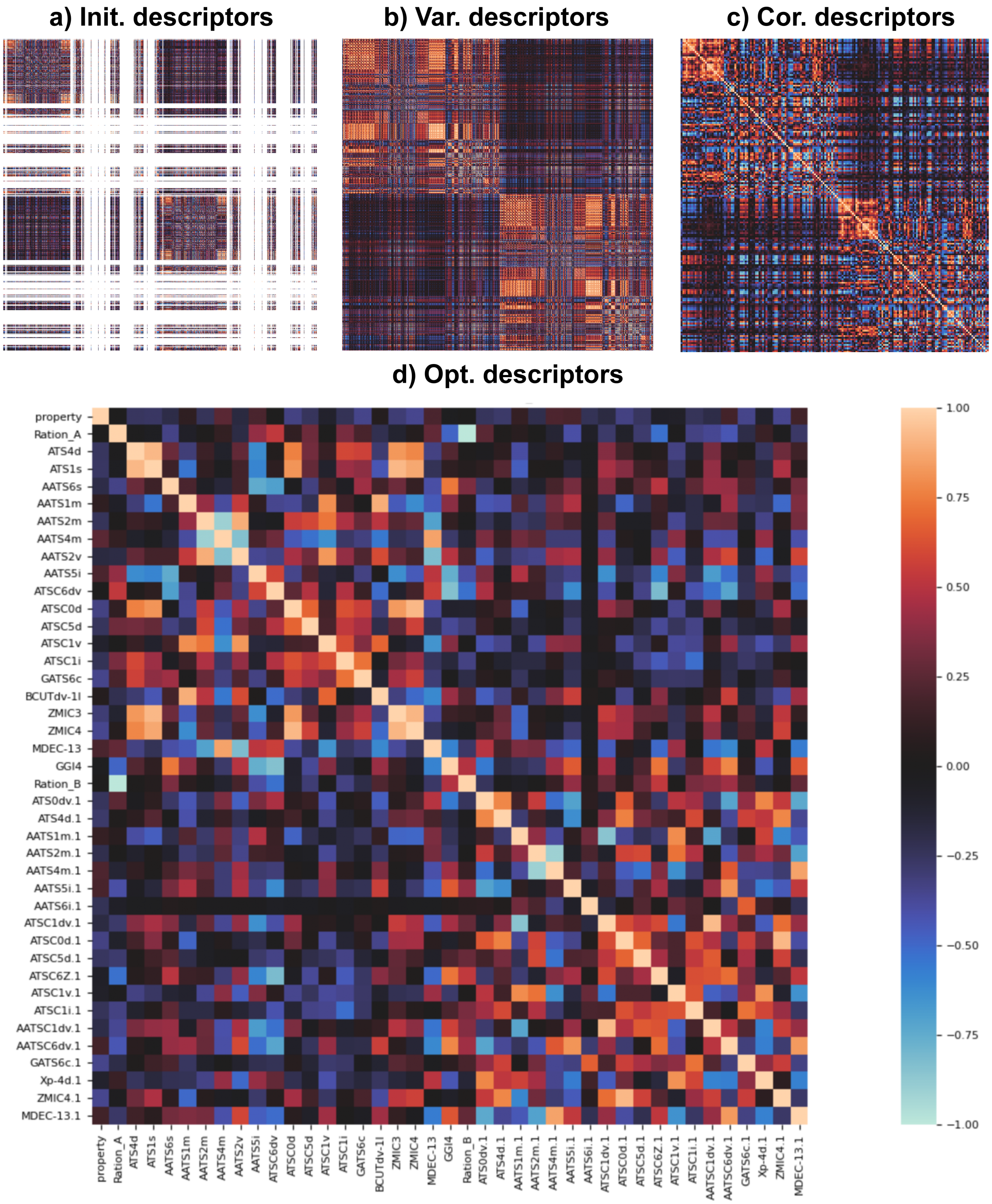

Supplementary Fig. 5 Spearman rank correlation matrices in the downselection process for MIC$_{E.\ coli}$. a) Init. descriptors, b) Var. descriptors, c) Cor. descriptors and d) Opt. descriptors. Values close to 0 mean low correlations, while values near 1 or -1 mean high correlations.

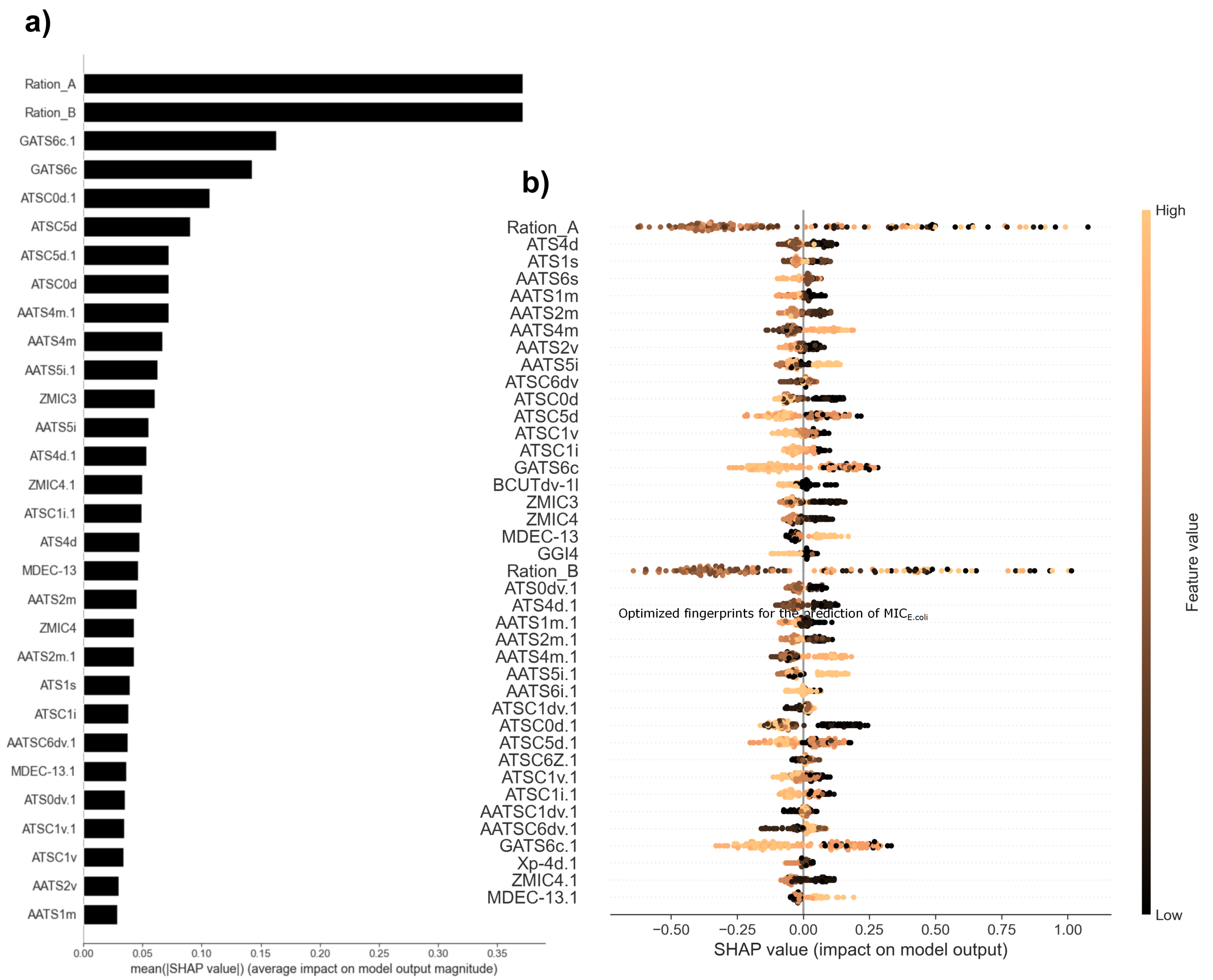

Supplementary Fig. 6 Importance ranking of descriptors and shapley additive explanations (SHAP) analysis for MIC$_{E.\ coli}$. a) Importance ranking of descriptors for property of MIC$_{E.\ coli}$ chosen in the random forest regression process with using recursive feature elimination (RFE). b) Shapley additive explanations (SHAP) analysis of the optimized descriptors. Note that descriptors which are decorated with ".1" means the descriptors for the second subunit

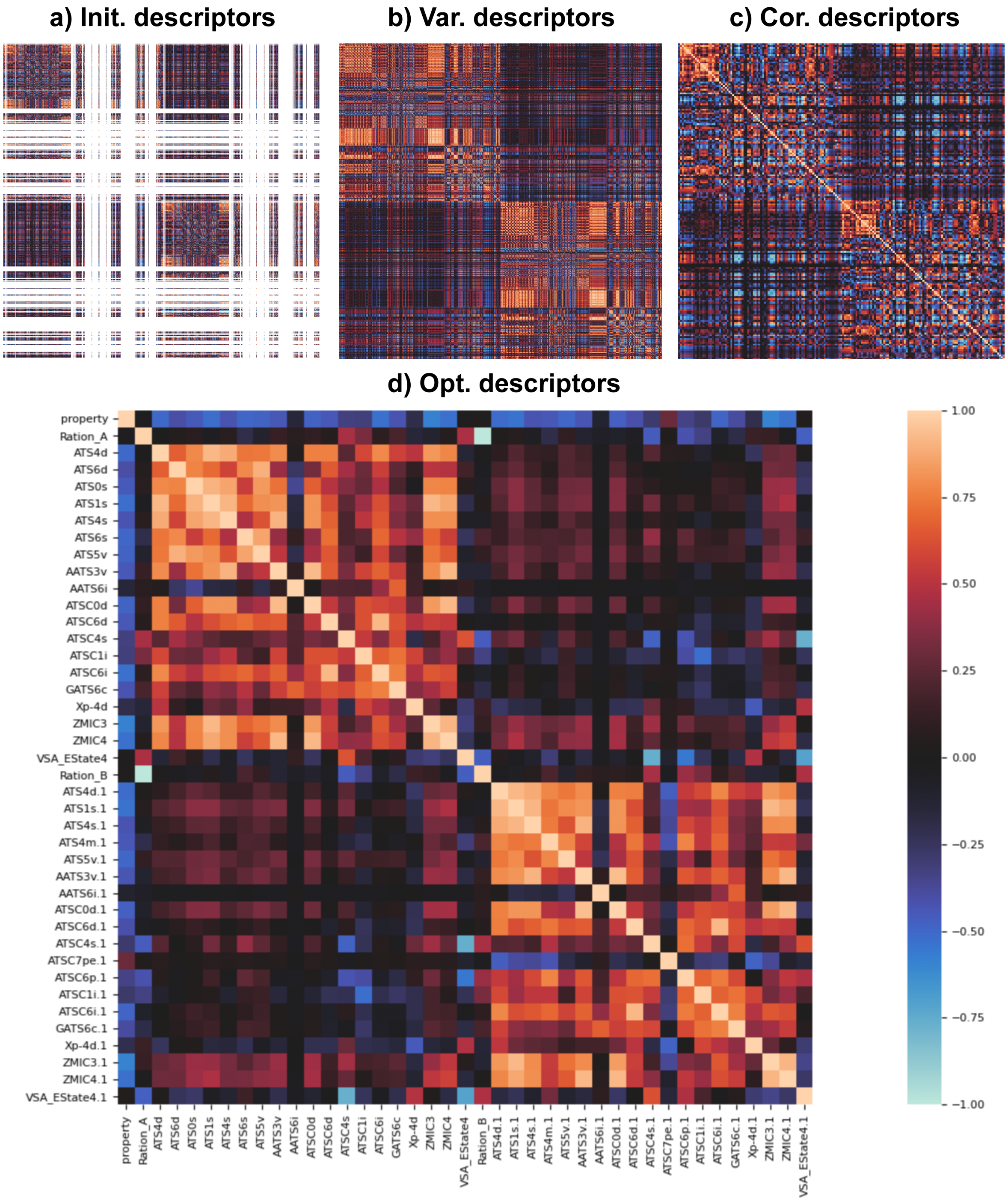

Supplementary Fig. 7 Spearman rank correlation matricesin the downselection process for HC_{10}. a) Init. descriptors, b) Var. descriptors, c) Cor. descriptors and d) Opt. descriptors. Values close to 0 mean low correlations, while values near 1 or -1 mean high correlations.

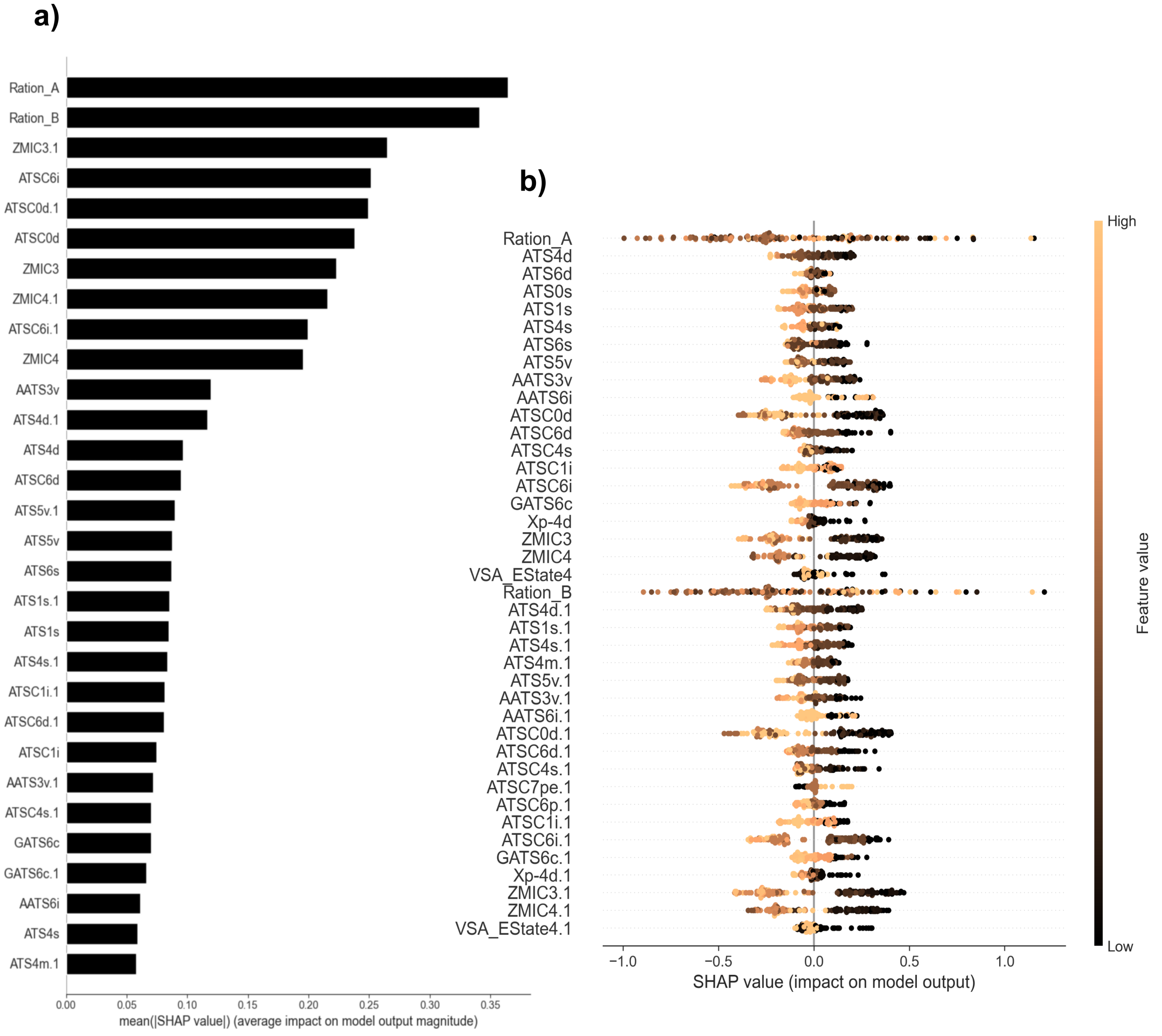

Supplementary Fig. 8 Importance ranking of descriptors and shapley additive explanations (SHAP) analysis for HC_{10}. a) Importance ranking of descriptors for property of HC_{10} chosen in the random forest regression process with using recursive feature elimination (RFE). b) Shapley additive explanations (SHAP) analysis of the optimized descriptors. Note that descriptors which are decorated with ".1" means the descriptors for the second subunit.

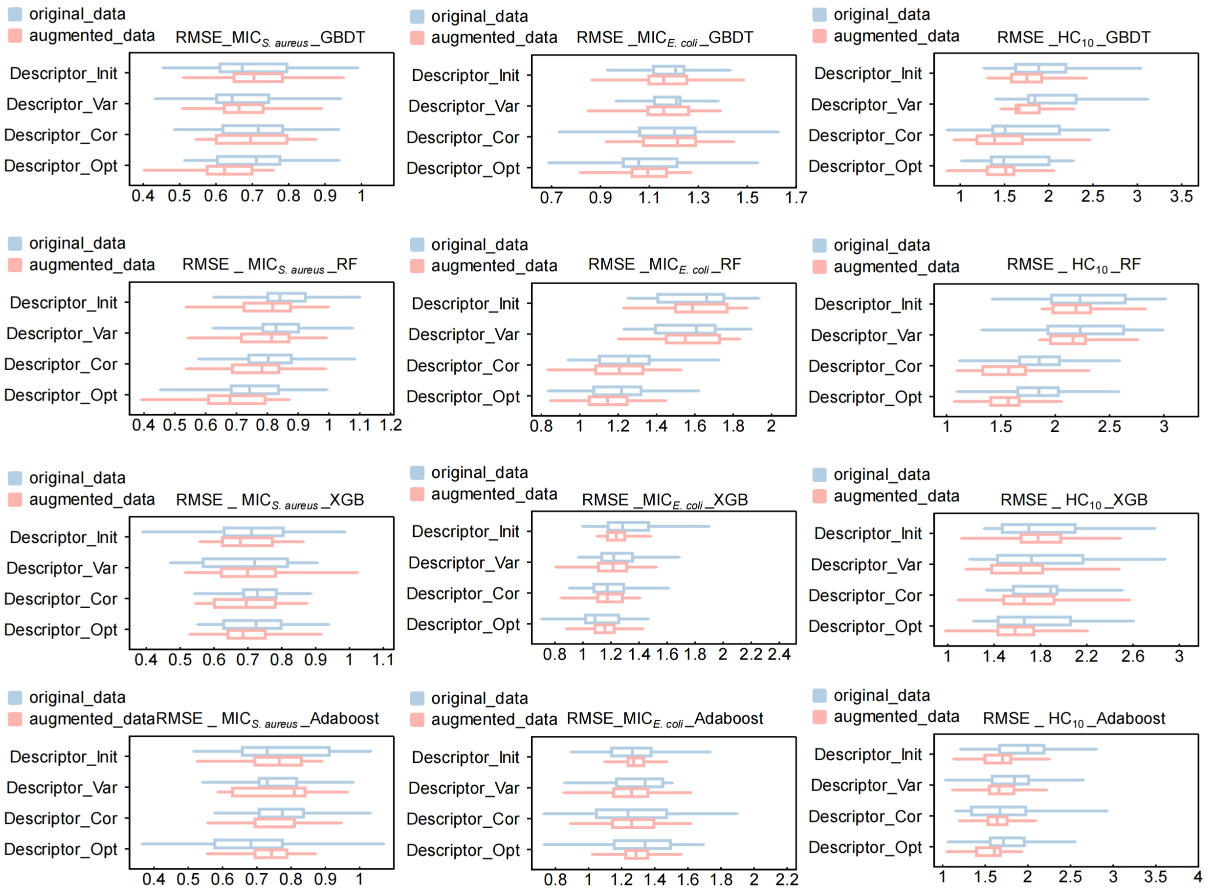

Supplementary Fig. 9 Cross validation results (n = 15 fold) on the value of MIC$_{S. aureus}$, MIC$_{E. coli}$ and HC$_{10}$ using GBDT, RF, XGB, Adaboost with and without augmentation using different selected descriptors with the metric of RMSE. The borders of the boxes indicate the first quartile (left) and the third quartile (right) of the results. The line in the box indicates the median. The whiskers refer to the most extreme, nonoutlier data points, with minima on the left and maxima on the right. With applying either descriptor downselection or data augmentation, the performance of the model can be improved.

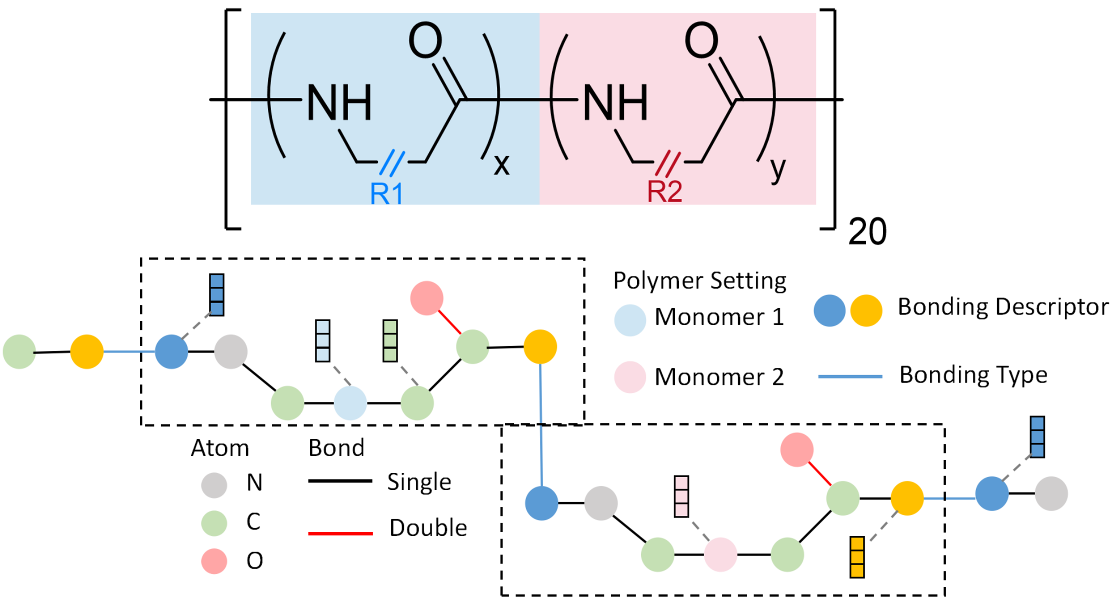

Supplementary Fig. 10 Overview of graph representation for cationic-hydrophobic amphiphilic β-amino acid polymer. Comparing with graph representation in micromolecule, several new polymer settings are introduced referenced from BigSMILES syntax. In additional to the normal molecular graph settings, we further introduce the bonding descriptors as new nodes and the edges between them. We use the graph in dashed box as the input graph.

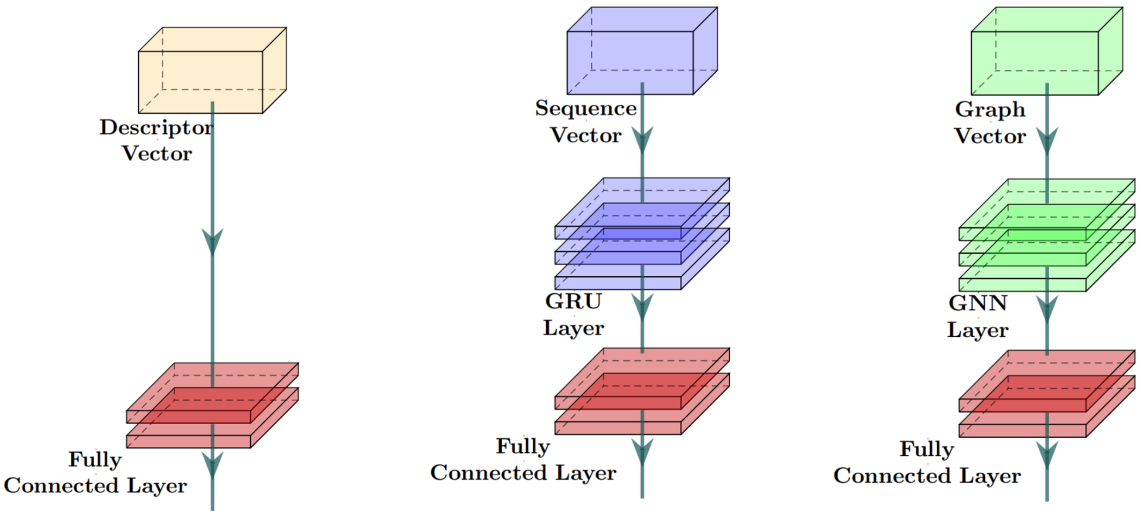

Supplementary Fig. 11 Overview of network structures for different single input. (left) Descriptor vector, (middle) Sequence vector, (right) Graph vector.

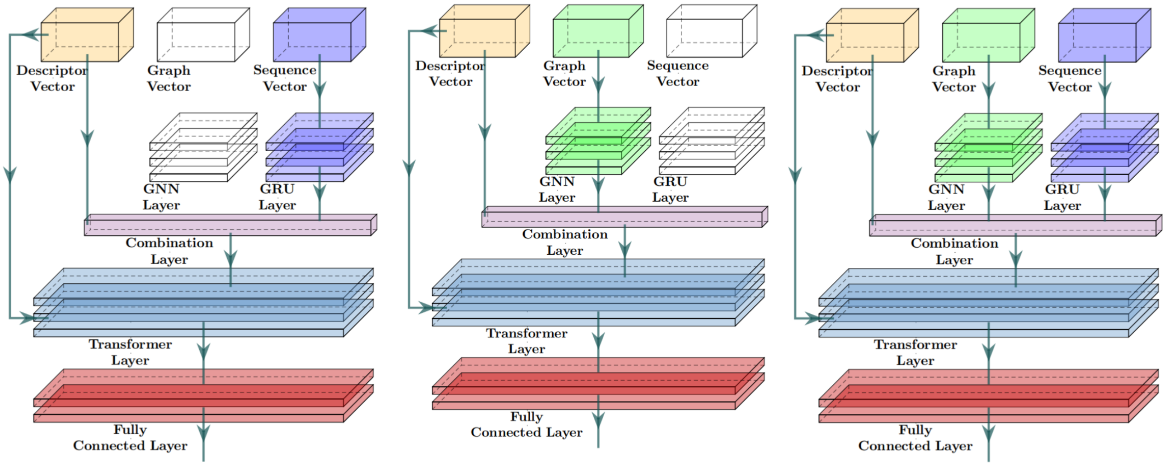

Supplementary Fig. 12 Overview of network structures for different combination input. (left) Sequence vector and Descriptor vector, (middle) Graph vector and Descriptor vector, (right) Sequence vector, graph vector and Descriptor vector.

Algorithm 1 Bidirectional Message Communication

Input: Graph G=$(\mathcal{V}, \mathcal{E})$; atom and bond attributes$\{x_v$,$\forall v \in \mathcal{V}$; $x_{e_{vw}}$, $\forall e_{vw} \in \mathcal{E}\}$; aggregate functions $\textbf{aggregate}_\mathbf{v}$ and $\textbf{aggregate}_\mathbf{e}$, communicate functions $\textbf{comm}_\mathbf{v}$ and $\textbf{comm}_\mathbf{e}$, update functions U_v and U_e, and readout functions $\textbf{readout}_\mathbf{v}$ and $\textbf{readout}_\mathbf{e}$ for nodes and edges respectively; network depth T.

Let $h_v^0 \leftarrow x_v, \forall v \in \mathcal{V}$; $h_{e_{vw}}^0 \leftarrow x_{e_{vw}}, \forall e_{vw} \in \mathcal{E}$
for $t = 0 \cdots T-1$ **do**
  for $\forall v \in \mathcal{V}$ **do**
    $m_v^{t+1} \leftarrow \textbf{aggregate}_\mathbf{e}(\{h_{e_{uv}}^t, u \in \mathcal{N}(v)\})$
    $p_v^{t+1} \leftarrow \textbf{comm}_\mathbf{v}(m_v^{t+1}, h_v^t)$
  end for
  for $\forall e \in \mathcal{E}$ **do**
    $m_{e_{vw}}^{t+1} \leftarrow \textbf{aggregate}_\mathbf{v}(h_v^t, h_w^t)$
    $p_{e_{vw}}^{t+1} \leftarrow \textbf{comm}_\mathbf{e}(m_{e_{vw}}^{t+1}, h_{e_{vw}}^t)$
  end for
  for $\forall e \in \mathcal{E}$ **do**
    $p_{e_{vw}}^{t+1} \leftarrow p_v^{t+1} - p_{e_{wv}}^{t+1}$
    $h_{e_{vw}}^{t+1} \leftarrow U_e^t(p_{e_{vw}}^{t+1}, h_{e_{vw}}^0)$
  end for
  for $\forall v \in \mathcal{V}$ **do**
    $h_v^{t+1} \leftarrow U_v^t(p_v^{t+1}, h_v^0)$
  end for
end for
$m_v^T \leftarrow \textbf{aggregate}_\mathbf{e}(\{h_{e_{uv}}^{T-1}, u \in \mathcal{N}(v)\})$
$h_v^T \leftarrow \textbf{comm}_\mathbf{v}(m_v^T, h_v^{T-1})$
$m_{e_{vw}}^T \leftarrow \textbf{aggregate}_\mathbf{v}(h_v^{T-1}, h_w^{T-1}, x_v)$
$h_{e_{vw}}^T \leftarrow \textbf{comm}_\mathbf{e}(m_{e_{vw}}^T, h_{e_{vw}}^{T-1}, x_{e_{vw}})$
$H_V = \textbf{readout}_\mathbf{v}(\{h_v^T, \forall v \in \mathcal{V}\})$
$H_E = \textbf{readout}_\mathbf{e}(\{h_e^T, \forall e \in \mathcal{E}\})$

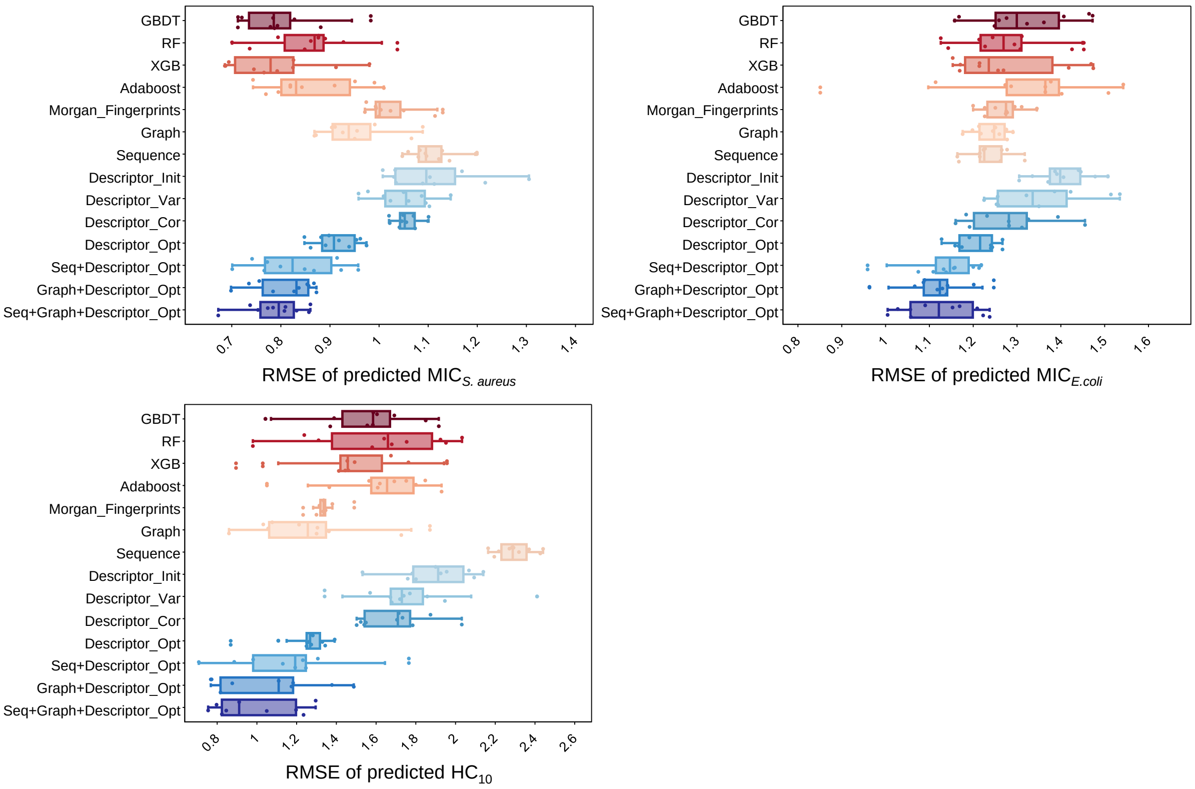

Supplementary Fig. 13 Predictive results on the value of MIC$_{S.\ aureus}$, MIC$_{E.\ coli}$ and HC$_{10}$ with the metric of RMSE with different polymer representation combination (n = 10). The borders of the boxes indicate the first quartile (left) and the third quartile (right) of the results. The line in the box indicates the median. The whiskers refer to the most extreme, nonoutlier data points, with minima on the left and maxima on the right. "Seq" is the abbreviation of "Sequence".

Poly(α-amino acid)s	x:y	Predicted values (μg mL^{-1})			Real values (μg mL^{-1}) [1]		
		HC$_{50}$	MIC$_{S.aureus}$	MIC$_{E.coli}$	HC$_{50}$	MIC$_{S.aureus}$	MIC$_{E.coli}$
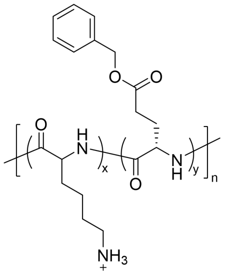	9:1	N/A	20.99	37.84	N/A	12	12
	8:2		16.03	35.52		25	12
	7:3		22.46	35.10		25	25
	6:4		19.46	37.07		25	25
	5:5		23.83	37.69		25	25
	4:6		24.92	43.74		50	50

Supplementary Fig. 14 Quantitative predictive results for Poly(α-amino acid)s compared with real values. Values are reported from the corresponding literature[1]. We default n to 20 for prediction. N/A is not available, meaning that real values are not reported in the literature and we do not make corresponding predictions.

Polymethacrylates	x:y	Predicted values (μg mL^{-1})			Real values (μg mL^{-1}) [2] [3]		
		HC$_{50}$	MIC$_{S.aureus}$	MIC$_{E.coli}$	HC$_{50}$	MIC$_{S.aureus}$	MIC$_{E.coli}$
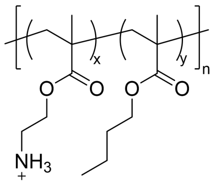	2:8	10.38	N/A	92.39	40	N/A	10
	2.8:7.2	10.81		92.08	16		1.1
	3.7:6.3	10.24		94.00	16		0.3
	4.5:5.5	9.62		95.69	16		0.8
	5.3:4.7	9.65		97.42	16		1
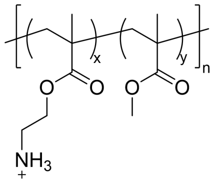	0:10	132.94	61.72	923.63	>2000	125	500
	1.2:8.8	165.29	63.95	483.30	>2000	125	500
	2.8:7.2	173.47	56.04	557.51	>2000	250	500
	4.7:6.3	152.30	65.53	585.97	>2000	125	63
	6.3:3.7	180.02	57.18	680.04	114	125	16

Supplementary Fig. 15 Quantitative predictive results for polymethacrylates compared with real values. Values are reported from the corresponding literature[2,3]. We default n to 20 for prediction. N/A is not available, meaning that real values are not reported in the literature and we do not make corresponding predictions.

Polymethacrylates	x:y	Predicted values (μg mL^{-1})			Real values (μg mL^{-1}) [4]		
		HC_{50}	$MIC_{S.aureus}$	$MIC_{E.coli}$	HC_{50}	$MIC_{S.aureus}$	$MIC_{E.coli}$
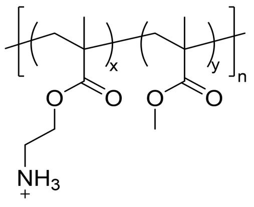	8.8:1.2	N/A		258.87	N/A		460
	3:7	N/A		204.67	N/A		460
	5.6:4.4	N/A	N/A	206.30	N/A	N/A	13
	4.4:5.6	11.27		204.10	100		10
	4:6	11.40		205.47	15		10
	3.6:6.4	12.02		202.78	10		10
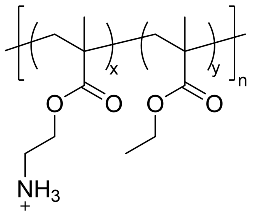	9:1	N/A		188.49	N/A		190
	8.4:1.6	N/A		178.60	N/A		170
	6.5:3.5	N/A	N/A	158.50	N/A	N/A	16
	5:5	43.89		150.86	1.3		2.9
	3:7	45.38		148.32	0.15		7.7
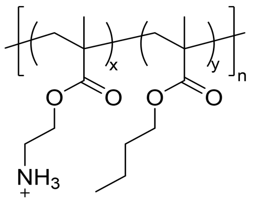	8.3:1.7	9.49		157.15	108		25
	7.6:2.4	9.22		144.49	15		13
	7.1:2.9	9.58		147.91	12		11
	6.8:3.2	9.77		138.37	6		8.8
	6.3:3.7	N/A	N/A	132.74	N/A	N/A	10
	6:4	9.13		128.40	1.7		5.3
	5.9:4.1	N/A		131.35	N/A		10
	5.6:4.4	9.23		123.82	1.3		10
	5.3:4.7	9.66		124.06	1.3		10

Supplementary Fig. 16 Quantitative predictive results for polymethacrylates compared with real values. Values are reported from the corresponding literature[4]. We default n to 20 for prediction. N/A is not available, meaning that real values are not reported in the literature and we do not make corresponding predictions.

Polymethacrylates	x:y	**Predicted values** (μg mL^{-1})			**Real values** (μg mL^{-1}) [4]		
		HC_{50}	$MIC_{S.aureus}$	$MIC_{E.coli}$	HC_{50}	$MIC_{S.aureus}$	$MIC_{E.coli}$
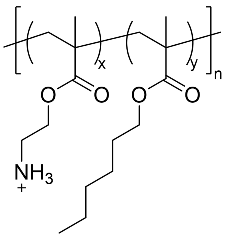	9.2:0.8	6.58		158.39	77		40
	8.4:1.6	6.40		146.25	10		12
	7.8:2.2	6.94	N/A	147.89	2.8	N/A	12
	7.3:2.7	6.92		141.90	1.3		12
	7:3	6.65		137.16	1		6
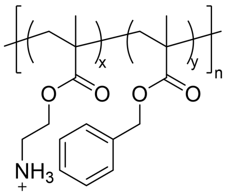	9.2:0.8	1.91		88.27	175		29
	8.4:1.6	1.88		81.00	10		15
	7.5:2.5	2.01	N/A	76.00	1	N/A	4.6
	6.7;3.3	2.02		68.97	0.24		4.6
	5.6:4.4	1.97		N/A	0.17		N/A
	4.9:5.1	2.03		N/A	0.16		N/A

Supplementary Fig. 17 Quantitative predictive results for polymethacrylates compared with real values. Values are reported from the corresponding literature[4]. We default n to 20 for prediction. N/A is not available, meaning that real values are not reported in the literature and we do not make corresponding predictions.

Polymethacrylates	x:y	Predicted values (μg mL^{-1})			Real values (μg mL^{-1}) [5]		
		HC_{50}	$MIC_{S.aureus}$	$MIC_{E.coli}$	HC_{50}	$MIC_{S.aureus}$	$MIC_{E.coli}$
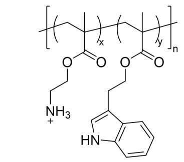	3.1:6.9		1.89			1500	
	1.9:8.1	N/A	1.31	N/A	N/A	94	N/A
	0.5:9.5		1.09			94	
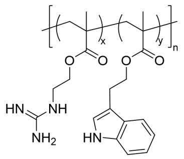	3.3:6.7		0.17			188	
	1.9:8.2	N/A	0.17	N/A	N/A	94	N/A
	0.6:9.4		0.17			47	

Supplementary Fig. 18 Quantitative predictive results for polymethacrylates compared with real values. Values are reported from the corresponding literature[5]. We default n to 20 for prediction. N/A is not available, meaning that real values are not reported in the literature and we do not make corresponding predictions.

Polymethacrylamides	x:y	Predicted values (μg mL^{-1})			Real values (μg mL^{-1}) [6]		
		HC_{50}	$MIC_{S.aureus}$	$MIC_{E.coli}$	HC_{50}	$MIC_{S.aureus}$	$MIC_{E.coli}$
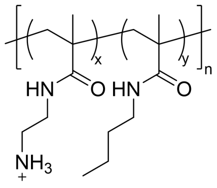	0:10	197.00	56.80	345.34	>1000	1.7	19
	2:8	163.77	58.50	307.74	>1000	13	278
	3.6:6.4	131.23	59.66	290.37	>1000	100	464
	5.4:4.6	93.47	60.37	303.43	>1000	117	170
	7.8:2.2	71.96	64.17	356.89	>1000	46	100
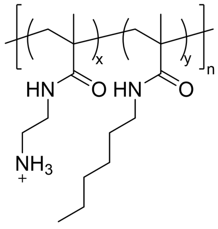	1.8:8.2	22.93	61.94	273.34	300	10	273.34
	3.3:6.7	20.59	56.11	257.12	12.5	13	257.12
	5.1:4.9	15.72	67.02	266.89	<0.16	11	266.89
	6.3:3.7	14.20	50.55	280.01	<0.16	13	280.01

Supplementary Fig. 19 Quantitative predictive results for polymethacrylamides compared with real values. Values are reported from the corresponding literature[6]. We default n to 20 for prediction. N/A is not available, meaning that real values are not reported in the literature and we do not make corresponding predictions.

Polynorbornenes	x:y	Predicted values (μg mL^{-1})			Real values (μg mL^{-1}) [7]		
		HC_{50}	$MIC_{S.aureus}$	$MIC_{E.coli}$	HC_{50}	$MIC_{S.aureus}$	$MIC_{E.coli}$
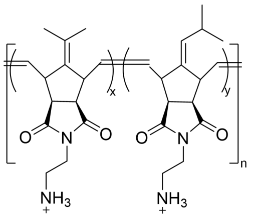	9:1	0.26	N/A	5897	>4000	N/A	40
	6.7:3.3	0.24		5855	>4000		40
	3.3:6.7	0.21		6017	<1		40
	2:8	0.2		6148	<1		40

Supplementary Fig. 20 Quantitative predictive results for polynorbornenes compared with real values. Values are reported from the corresponding literature[7]. We default n to 20 for prediction. N/A is not available, meaning that real values are not reported in the literature and we do not make corresponding predictions.

Pyridinium polymers	m	Predicted values (μg mL^{-1})			Real values (μg mL^{-1}) [8]		
		HC_{50}	$MIC_{S.aureus}$	$MIC_{E.coli}$	HC_{50}	$MIC_{S.aureus}$	$MIC_{E.coli}$
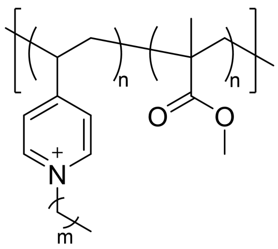	2	15.10	N/A	247.73	2393	N/A	600
	3	19.28		262.08	1897		200
	4	25.82		226.94	1709		30
	6	18.55		217.29	351		100
	8	17.89		215.47	229		450
	10	15.07		217.14	393		1100
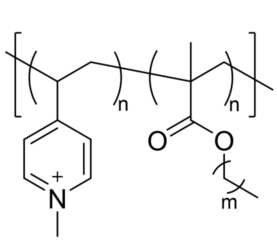	2	67.70	N/A	284.15	1147	N/A	350
	3	11.91		257.17	108		100
	4	5.08		247.71	0.23		15
	6	1.76		225.81	0.15		50
	8	1.23		221.29	0.11		125
	10	0.53		206.77	0.83		650

Supplementary Fig. 21 Quantitative predictive results for pyridinium polymers compared with real values. Values are reported from the corresponding literature[8]. We default n to 20 for prediction. N/A is not available, meaning that real values are not reported in the literature and we do not make corresponding predictions. The column for "m" means the number of atoms of carbon connected in the place.

Poly(vinyl ether)s	x:y	Predicted values (μg mL^{-1})			Real values (μg mL^{-1}) [9]		
		HC_{50}	$MIC_{S.aureus}$	$MIC_{E.coli}$	HC_{50}	$MIC_{S.aureus}$	$MIC_{E.coli}$
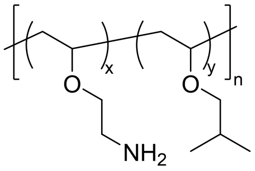	7.5:2.5	922.76		409.38	0.49		1.6
	4.7:5.3	894.94	N/A	380.92	1.8	N/A	3.1
	2.1:7.9	1062.14		375.50	18.9		31.3

Supplementary Fig. 22 Quantitative predictive results for poly(vinyl ether)s compared with real values. Values are reported from the corresponding literature[9]. We default n to 20 for prediction. N/A is not available, meaning that real values are not reported in the literature and we do not make corresponding predictions.

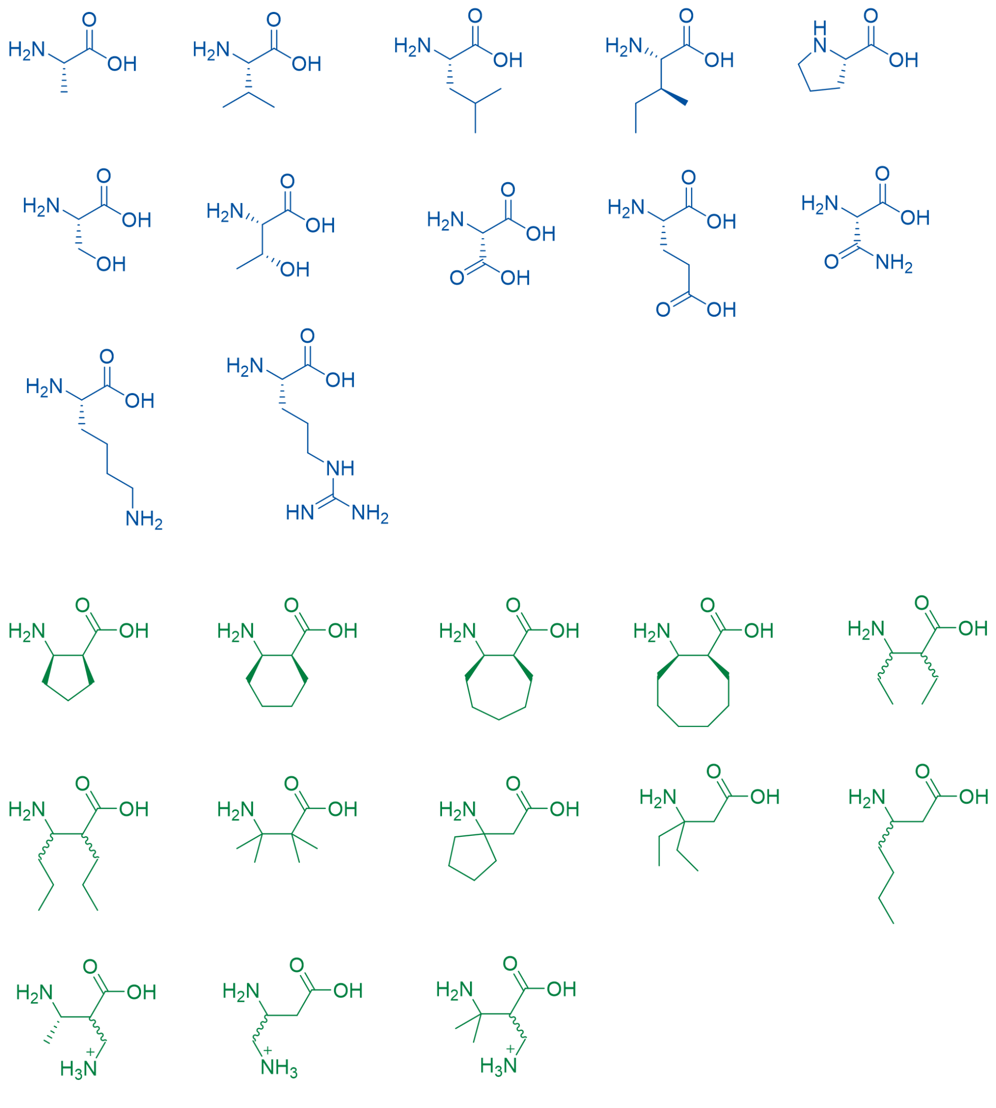

Supplementary Fig. 23 Data used for graph grammar distillation.

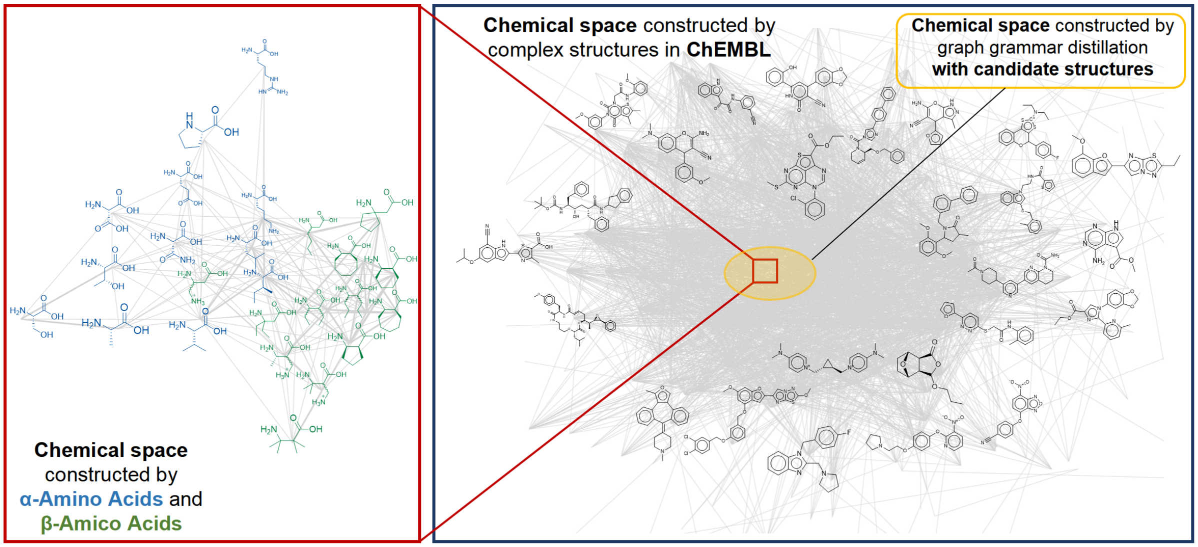

Supplementary Fig. 24 Visualization for graph grammar distillation. Generally, our β-amino acids have similar structures with natural α-amino acids (left). However, if we pre-training our model with large-scale public data, those rules for constructing complex structures or undesirable chemical elements (e.g. "Br","Cl") may also be embedded in the model (right). Thus, it takes a long time for further RL fine-tuning to adjust the parameters to avoid generating those undesirable subunits. While applying graph grammar distillation, all the grammar is distilled as new molecule set (yellow area). Thus the chemical space for the generative model to explore can be greatly restricted and more candidate structures can be found.

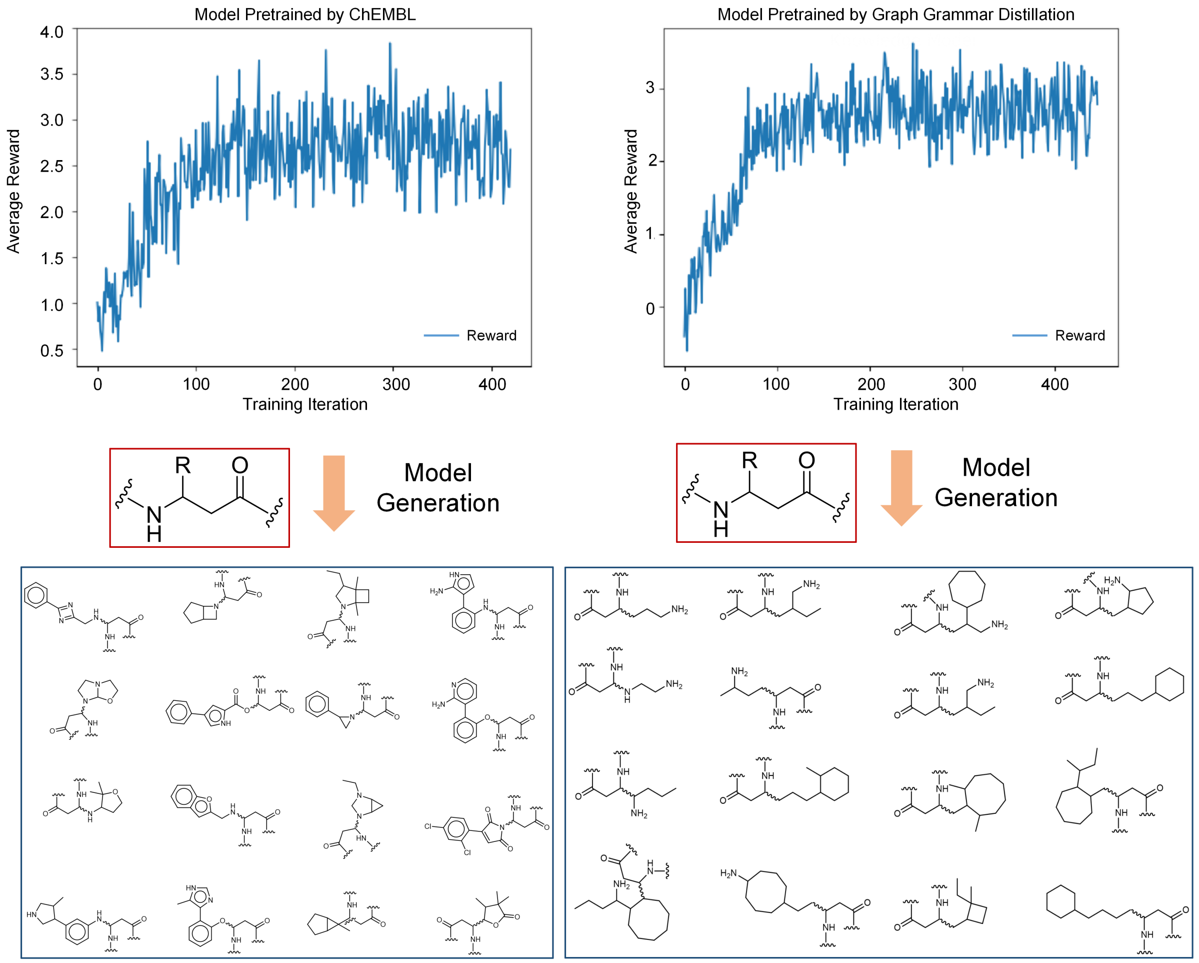

Supplementary Fig. 25 Comparison of the generated subunits with different pre-trained model. Left: Model pre-trained with ChEMBL dataset, Right: Model pre-trained with graph grammar distillation. Reward settings: $a = 1, b = 0, c = 0, X = 0, Y = 0$, with only property reward of $\text{MIC}_{\textit{S. aureus}}$ and the penalty of invalid molecules. Results show that both the pre-trained model can generate valid subunits. However, subunits generated by model pre-trained by ChEMBL dataset are generally too complex and hard to be synthesised.

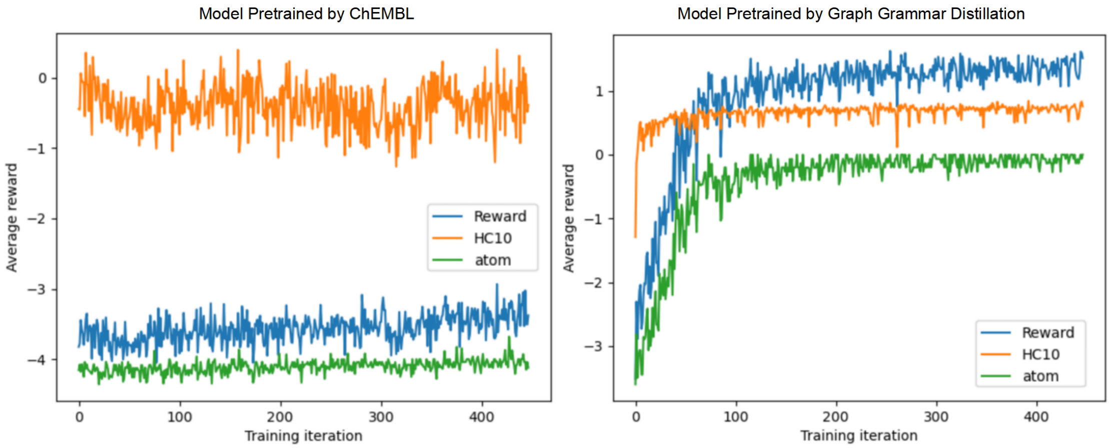

Supplementary Fig. 26 Comparison of RL training process for linear scaffold (scaffold 1-8 in Fig. S2) with property scores and structural constrains. Left: Model pre-trained with ChEMBL dataset, Right: Model pre-trained with graph grammar distillation. Reward settings: $a = 0, b = 0, c = 1, X = 11, Y = 1$, with only property reward of HC_{10}, the penalty of invalid molecules and unexpected elements, and carbon number constrain. Results exhibit that model pre-trained by ChEMBL dataset can not generate the expected molecules, while the reward curves of model pre-trained by graph grammar distillation are covergent.

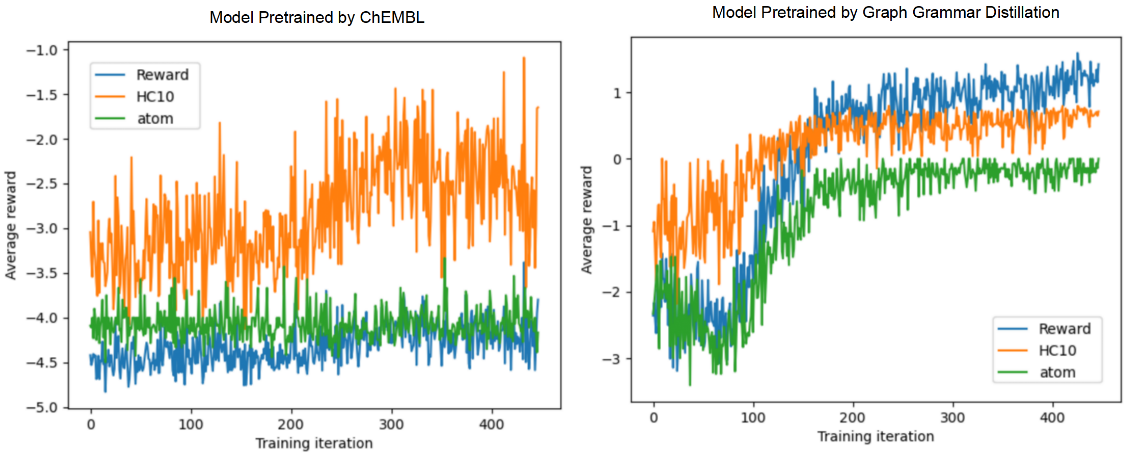

Supplementary Fig. 27 Comparison of RL training process for cyclic scaffold (scaffold 9-11 in Fig. S2) with property scores and structural constrains. Left: Model pre-trained with ChEMBL dataset, Right: Model pre-trained with graph grammar distillation. Reward settings: $a = 0, b = 0, c = 1, X = 11, Y = 1$, with only property reward of HC_{10}, the penalty of invalid molecules and unexpected elements, and carbon number constrain. Results exhibit that model pre-trained by ChEMBL dataset can not generate the expected molecules, while the reward curves of agent pre-trained by graph grammar distillation are covergent.

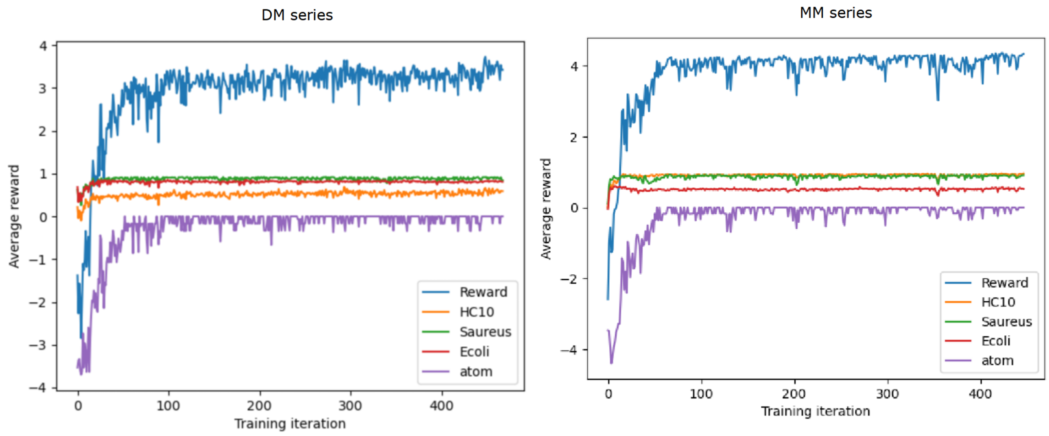

Supplementary Fig. 28 Comparison of RL training process for linear scaffold (scaffold 1-8 in Fig. S2) with all constrains. Left: fix the cationic subunit of the polymer as DM, Right: fix the cationic subunit of the polymer as MM. Reward settings: $a = 1, b = 1, c = 3, X = 11, Y = 1$, with only property reward of $\mathrm{MIC}_{S.\ aureus}$, $\mathrm{MIC}_{E.\ coli}$ HC_{10}, the penalty of invalid molecules and unexpected elements, and carbon number constrain. The results show that our model can fit any situation and our model can find the optimal or sub-optimal results even with various constrains.

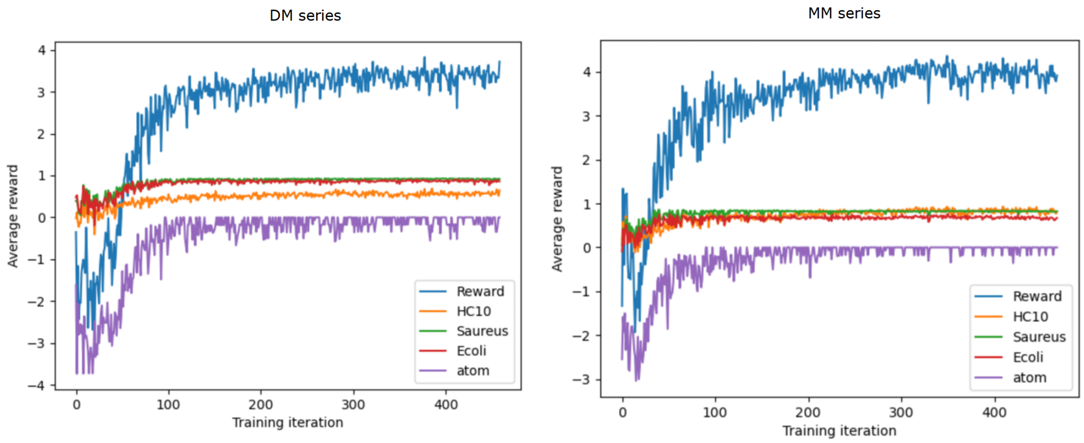

Supplementary Fig. 29 Comparison of RL training process for cyclic scaffold (scaffold 9-11 in Fig. S2) with all constrains. Left: fix the cationic subunit of the polymer as DM, Right: fix the cationic subunit of the polymer as MM. Reward settings: $a = 1, b = 1, c = 3, X = 11, Y = 1$, with only property reward of $\mathrm{MIC}_{S.\ aureus}$, $\mathrm{MIC}_{E.\ coli}$ HC_{10}, the penalty of invalid molecules and unexpected elements, and carbon number constrain. The results show that our model can fit any situation and our model can find the optimal or sub-optimal results even with various constrains.

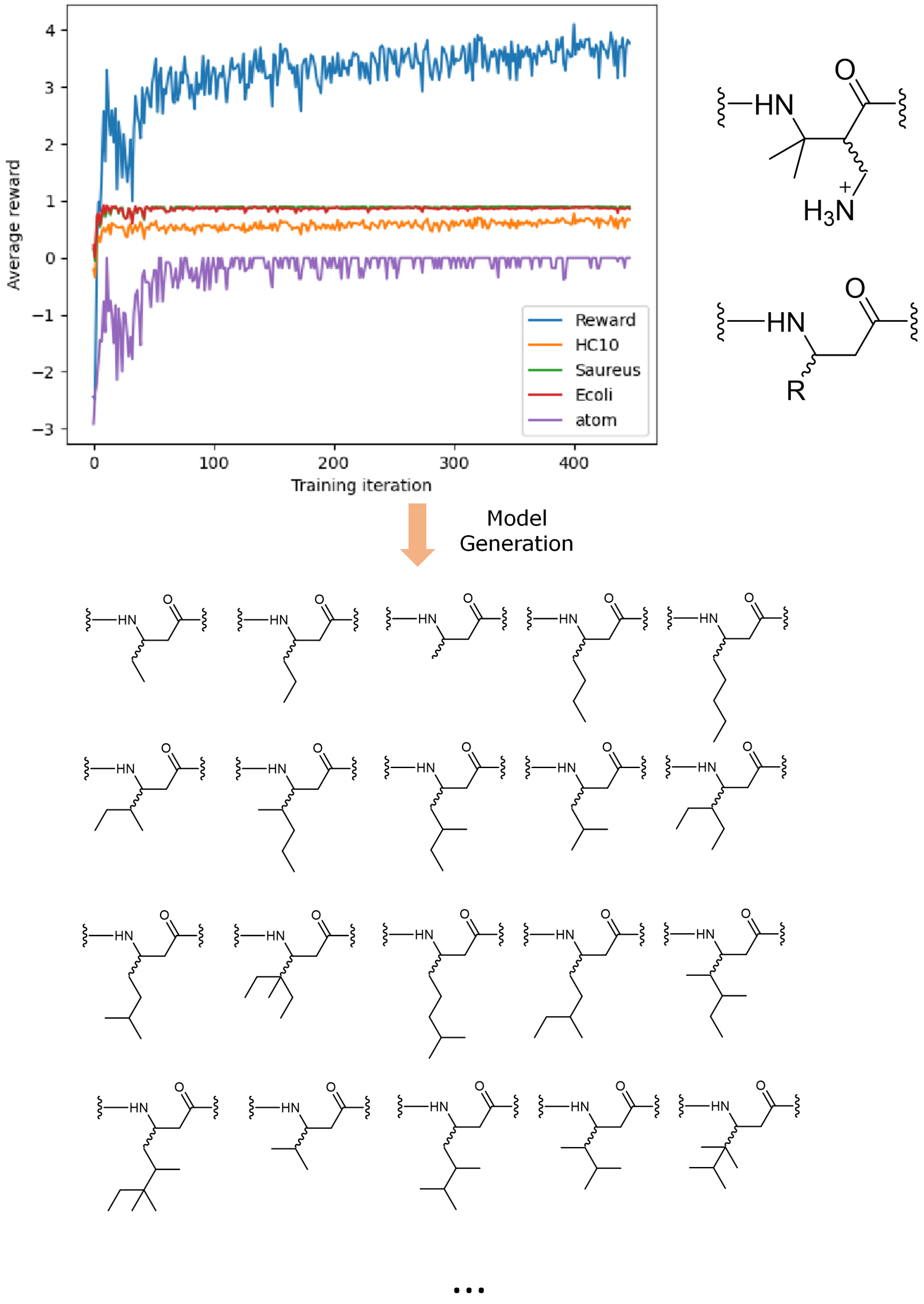

Supplementary Fig. 30 Generation setting: cationic:DM/hydrophobic: β^3-subunit. The fine-tuning process and the generated subunits in shown. Reward settings: $a = 1, b = 1, c = 3, X = 11, Y = 1$.

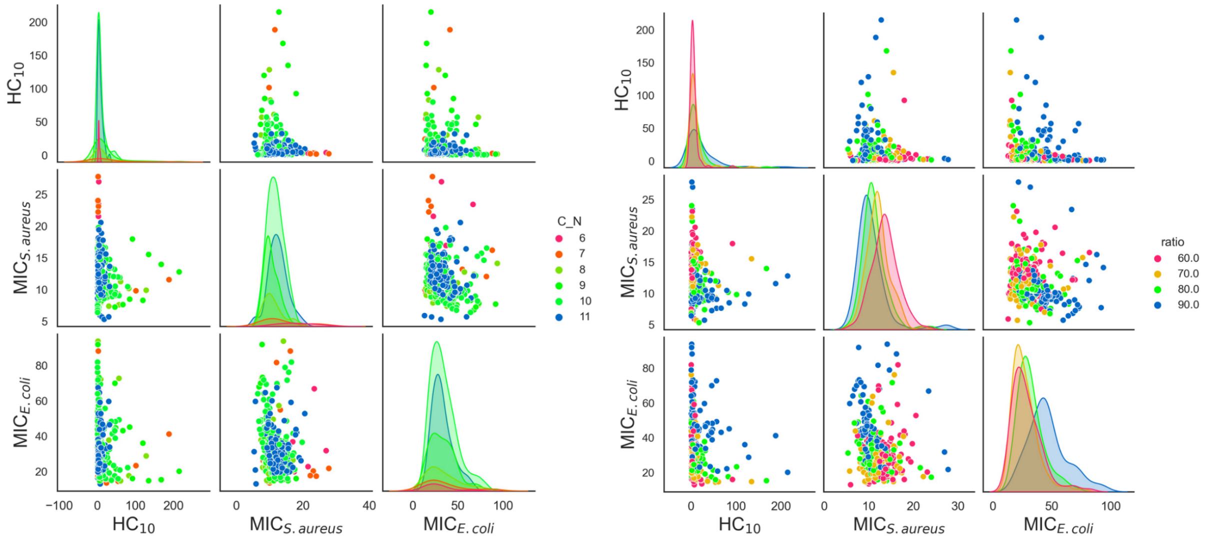

Supplementary Fig. 31 Generation setting:cationic:DM/hydrophobic: β^3-subunit. Property distribution of the predicted value of $\mathrm{MIC}_{S.\ aureus}$, $\mathrm{MIC}_{E.\ coli}$ and HC_{10}. The units for all properties are (μg mL^{-1}).

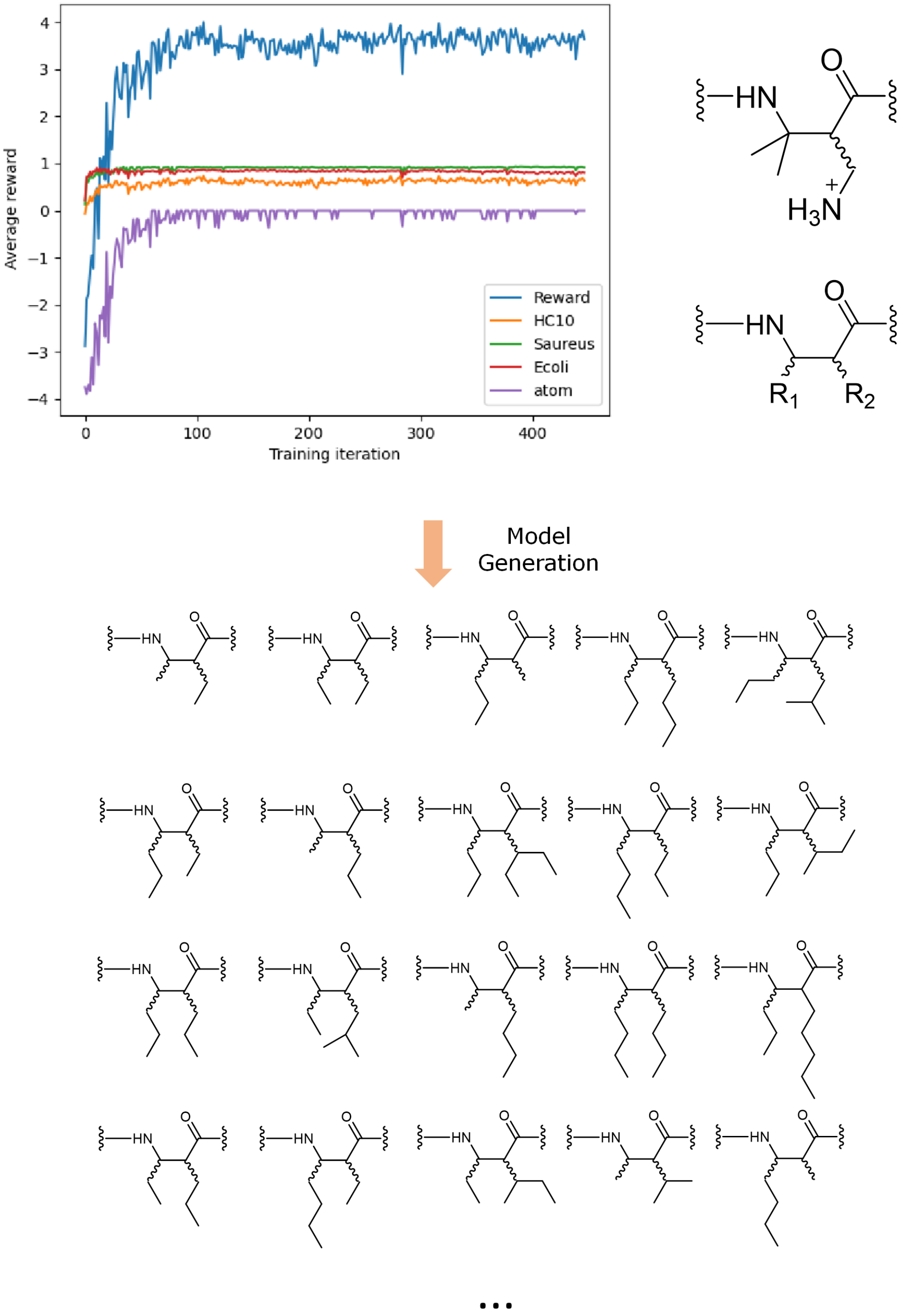

Supplementary Fig. 32 Generation setting:cationic:DM/hydrophobic: $\beta^{2,3}$-subunit. The fine-tuning process and the generated subunits is shown. Reward settings: $a = 1, b = 1, c = 3, X = 11, Y = 1$.

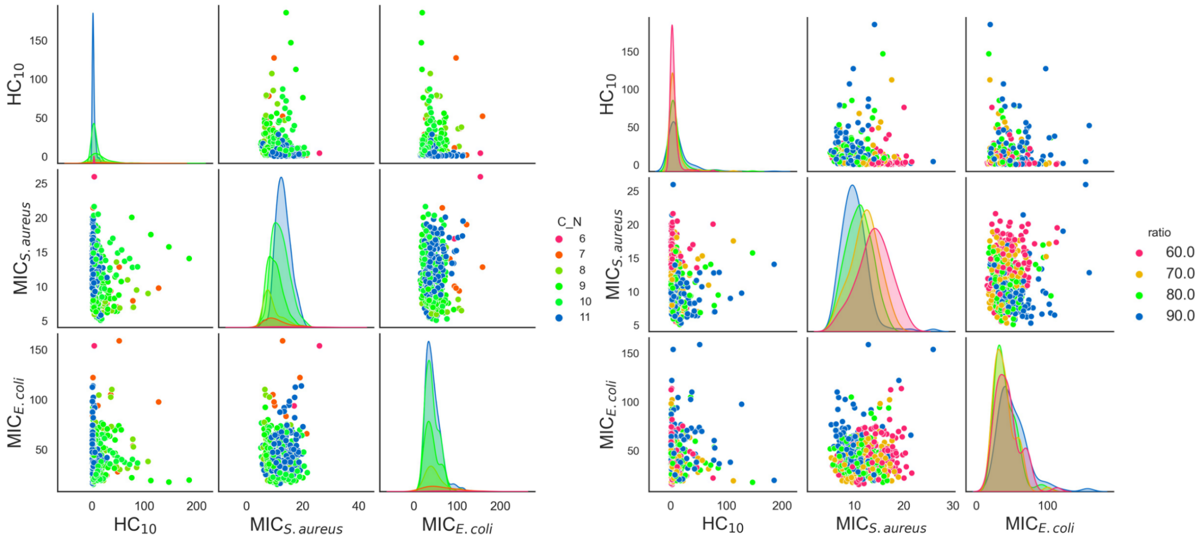

Supplementary Fig. 33 Generation setting: cationic:DM/hydrophobic: $\beta^{2,3}$**-subunit.** Property distribution of the predicted value of $\text{MIC}_{S.\ aureus}$, $\text{MIC}_{E.\ coli}$ and HC_{10}. The units for all properties are (μg mL^{-1}).

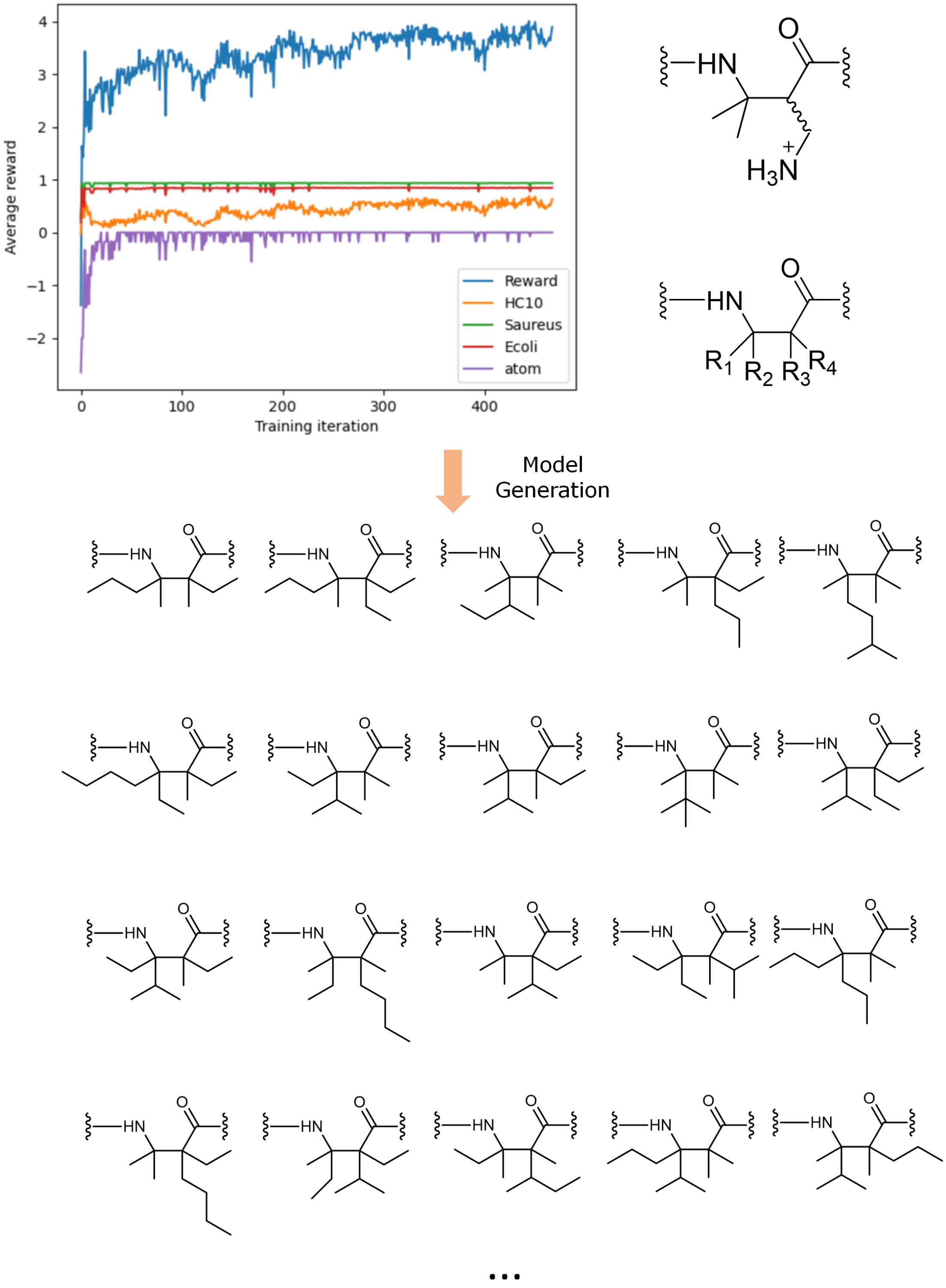

Supplementary Fig. 34 Generation setting:cationic:DM/hydrophobic: $\beta^{2,2,3,3}$-subunit. The fine-tuning process and the generated subunits is shown. Reward settings: $a = 1, b = 1, c = 3, X = 11, Y = 1$.

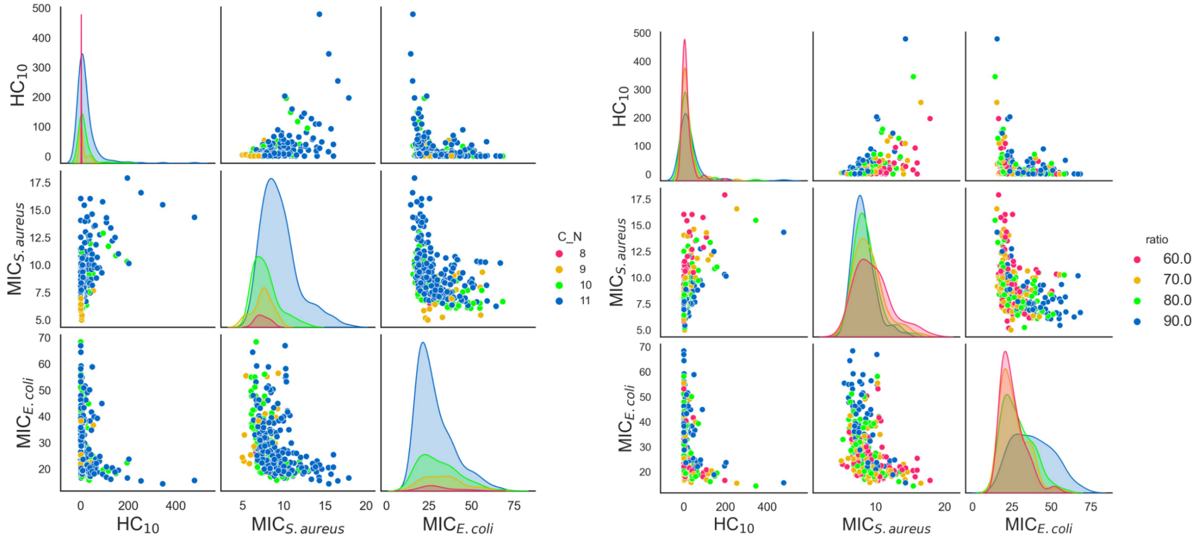

Supplementary Fig. 35 Generation setting:cationic:DM/hydrophobic: $\beta^{2,2,3,3}$-subunit. Property distribution of the predicted value of $MIC_{S.\ aureus}$, $MIC_{E.\ coli}$ and HC_{10}. The units for all properties are (μg mL^{-1}).

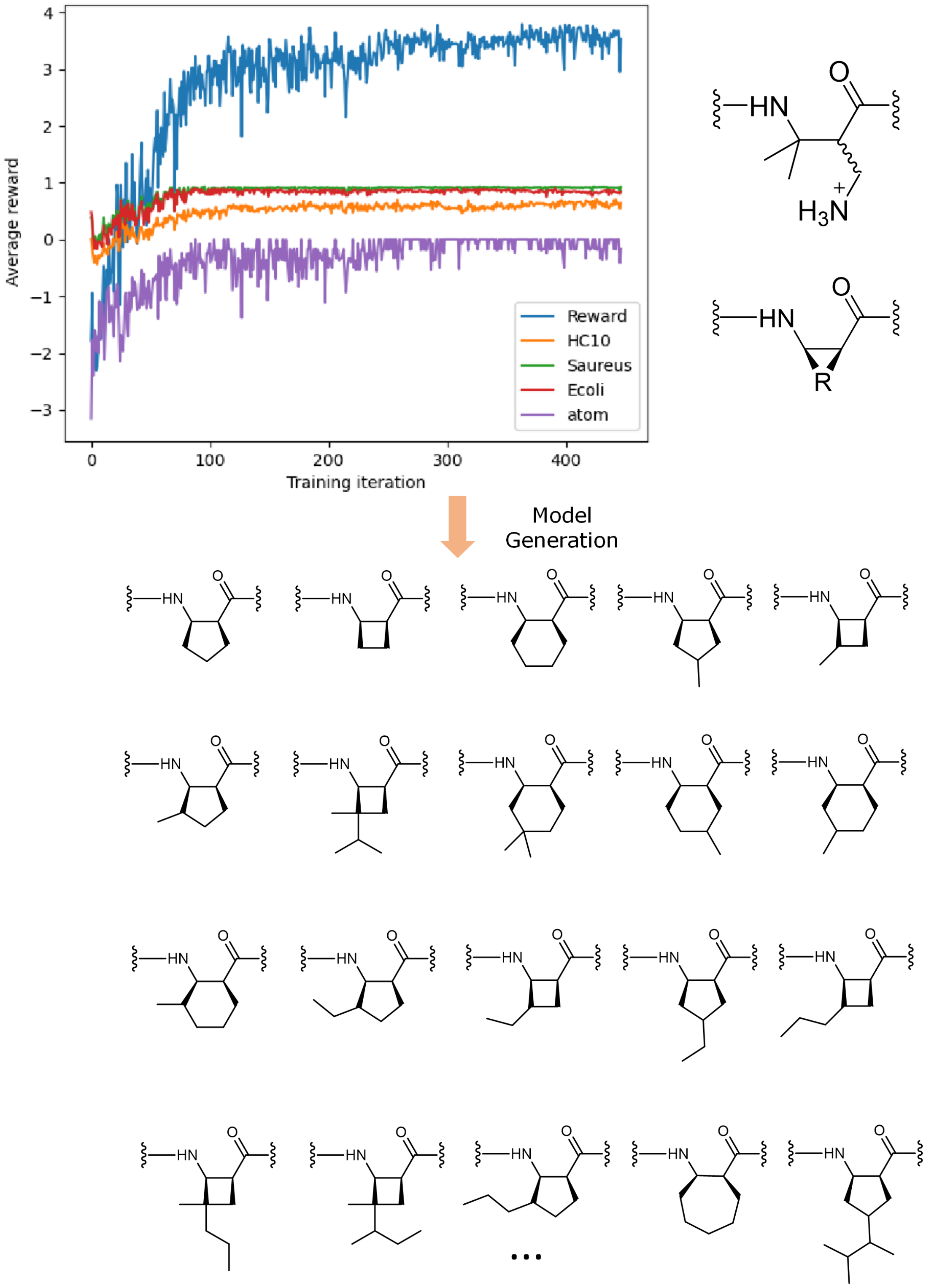

Supplementary Fig. 36 Generation setting: cationic:DM/hydrophobic: $\beta^{2,3}$-subunit cyclic. The fine-tuning process and the generated subunits is shown. Reward settings: $a = 1, b = 1, c = 3, X = 11, Y = 1$.

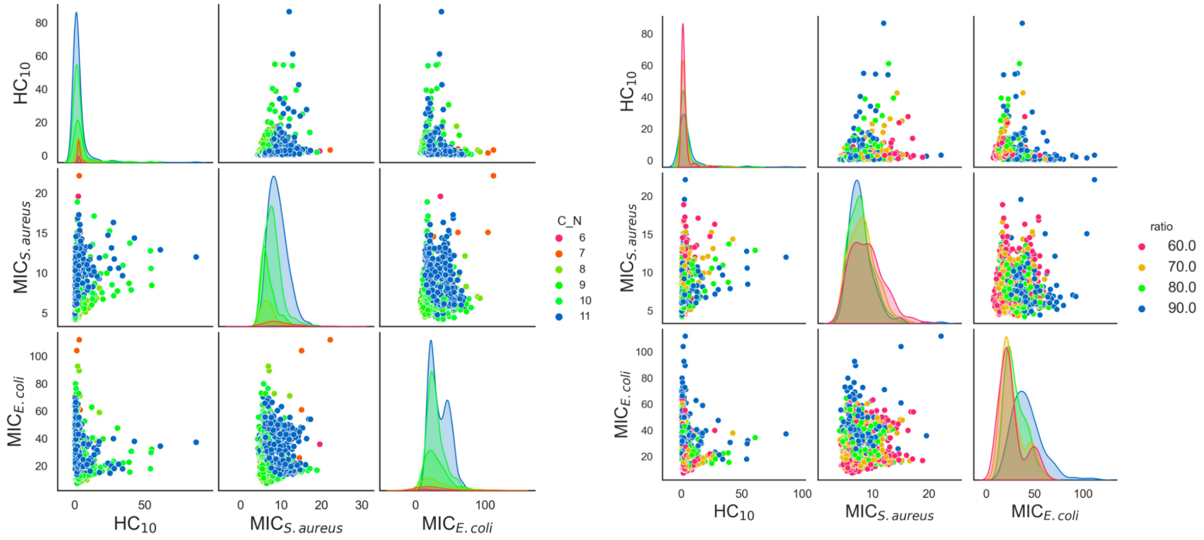

Supplementary Fig. 37 Generation setting: cationic:DM/hydrophobic: $\beta^{2,3}$-subunit cyclic. Property distribution of the predicted value of $\mathrm{MIC}_{S.\ aureus}$, $\mathrm{MIC}_{E.\ coli}$ and HC_{10}. The units for all properties are (µg mL^{-1}).

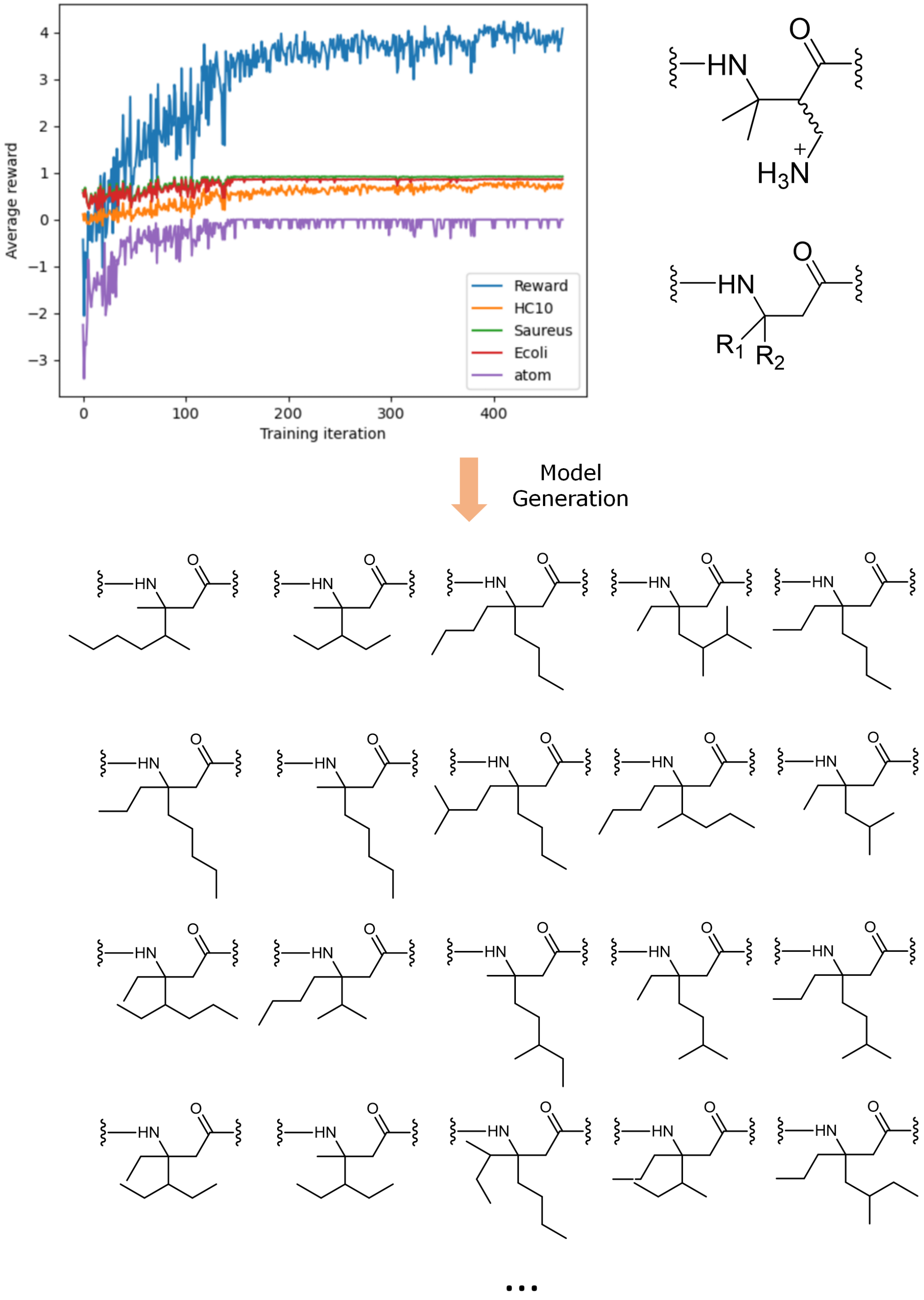

Supplementary Fig. 38 Generation setting: cationic:DM/hydrophobic: $\beta^{3,3}$-subunit. The fine-tuning process and the generated subunits is shown. Reward settings: $a = 1, b = 1, c = 3, X = 11, Y = 1$.

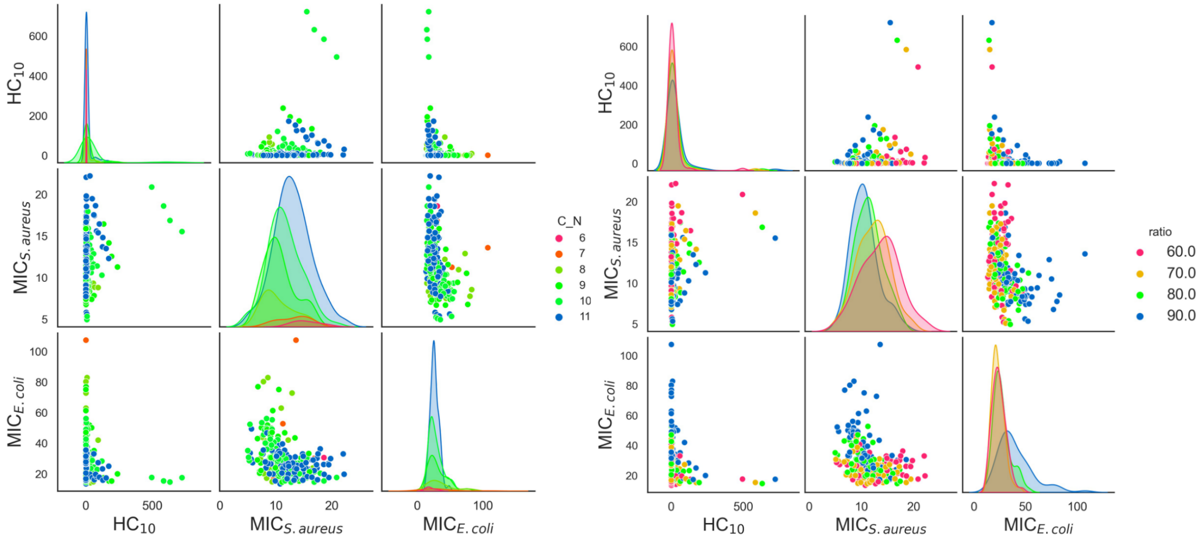

Supplementary Fig. 39 Generation setting: cationic:DM/hydrophobic: $\beta^{3,3}$**-subunit.** Property distribution of the predicted value of $\mathrm{MIC}_{S.\ aureus}$, $\mathrm{MIC}_{E.\ coli}$ and HC_{10}. The units for all properties are (μg mL^{-1}).

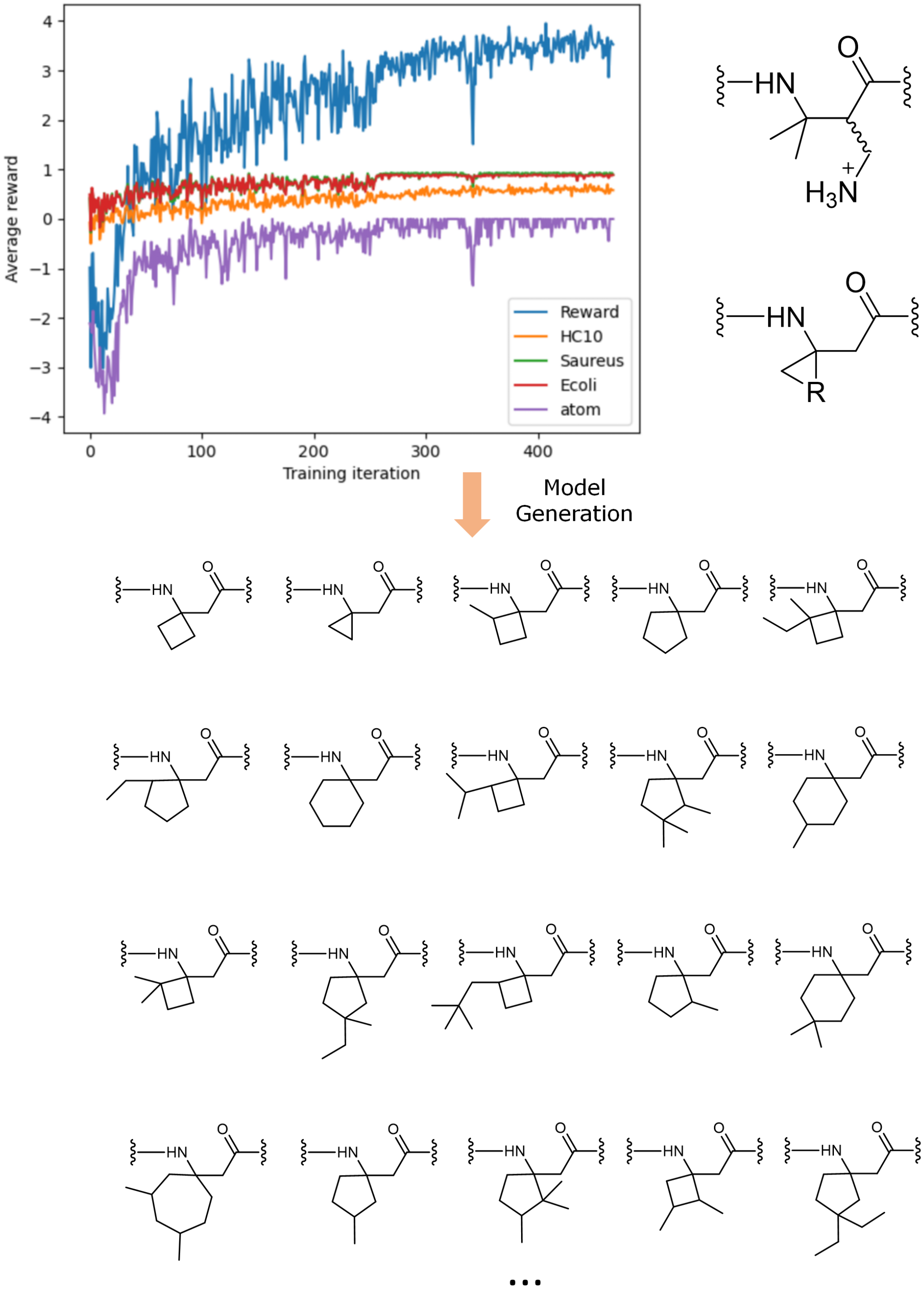

Supplementary Fig. 40 Generation setting: cationic:DM/hydrophobic: $\beta^{3,3}$-subunit cyclic. The fine-tuning process and the generated subunits is shown. Reward settings: $a = 1, b = 1, c = 3, X = 11, Y = 1$.

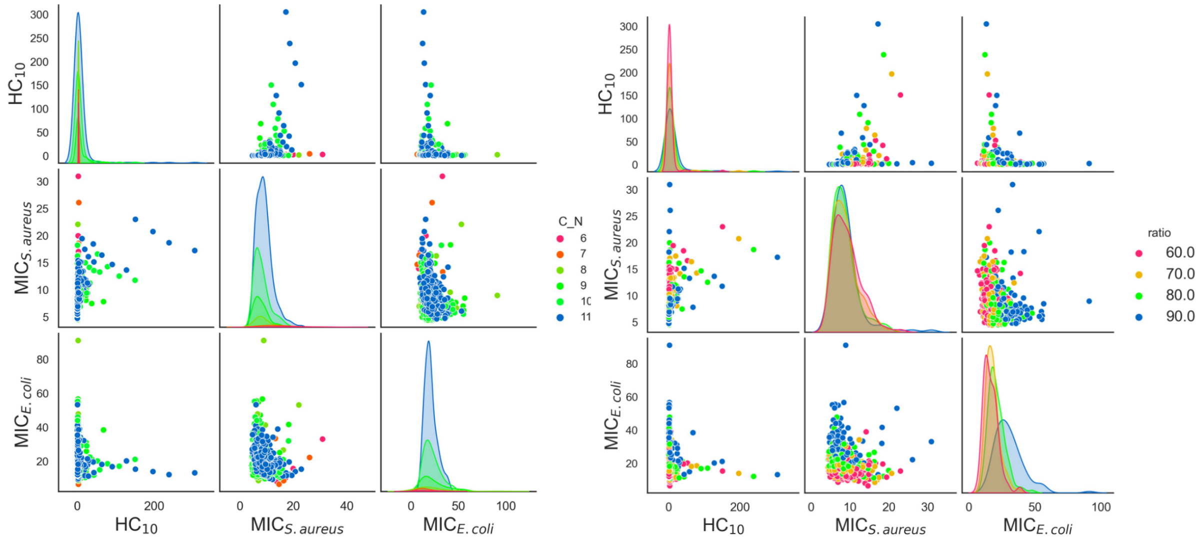

Supplementary Fig. 41 Generation setting: cationic:DM/hydrophobic: $\beta^{3,3}$-subunit cyclic. Property distribution of the predicted value of $\mathrm{MIC}_{S.\ aureus}$, $\mathrm{MIC}_{E.\ coli}$ and HC_{10}. The units for all properties are (µg mL^{-1}).

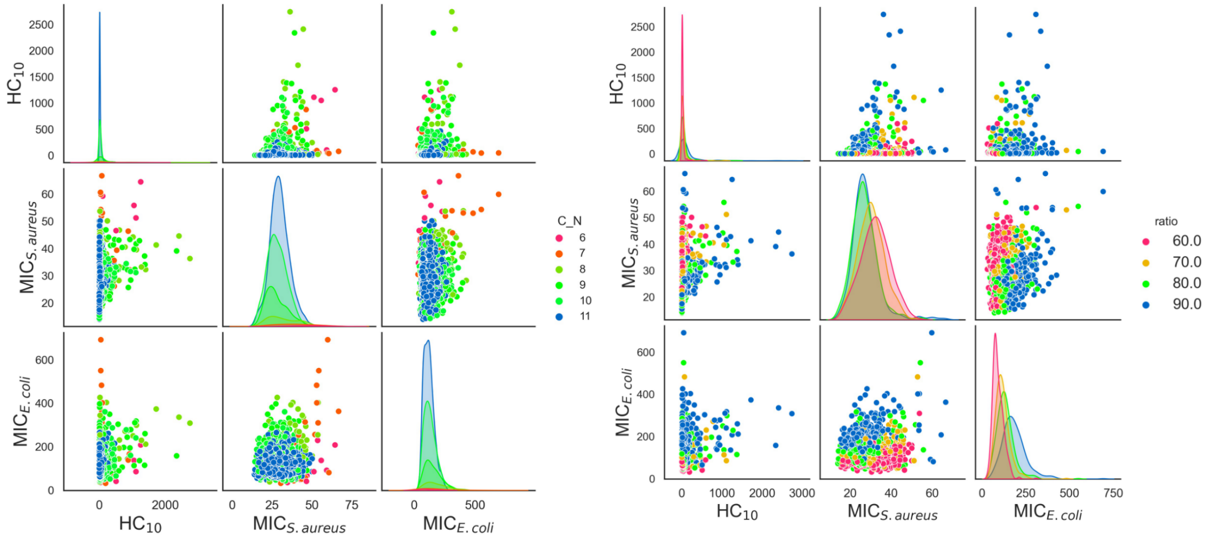

Supplementary Fig. 42 Generation setting:cationic:MM/hydrophobic: β^3-subunit. Property distribution of the predicted value of $\mathrm{MIC}_{S.\ aureus}$, $\mathrm{MIC}_{E.\ coli}$ and HC_{10} is shown. The units for all properties are (µg mL^{-1}). Reward settings: $a = 2, b = 2, c = 1, X = 11, Y = 1$.

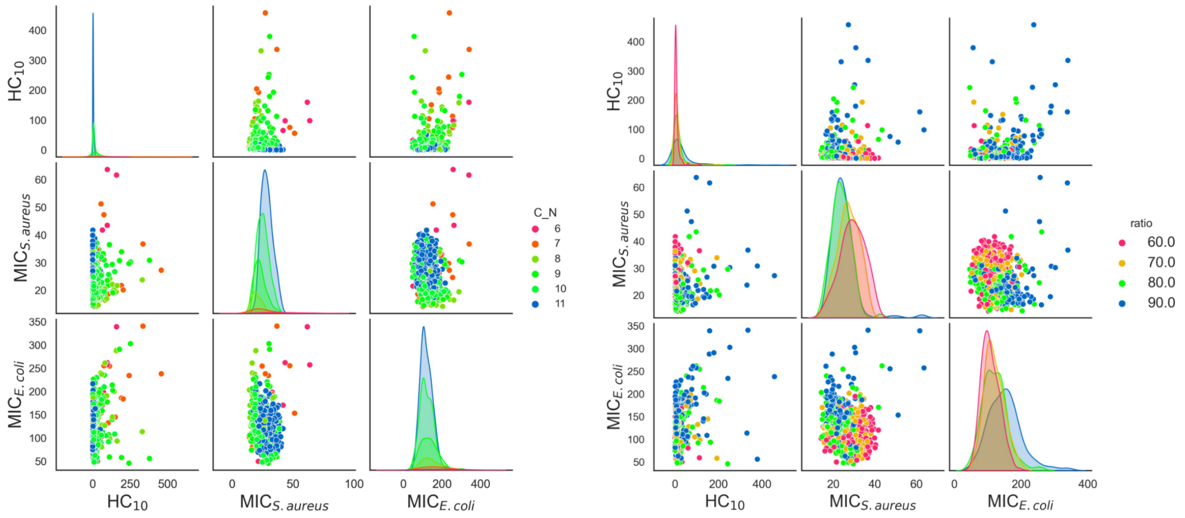

Supplementary Fig. 43 Generation setting: cationic:MM/hydrophobic: $\beta^{2,3}$-subunit. Property distribution of the predicted value of $\mathrm{MIC}_{S.\ aureus}$, $\mathrm{MIC}_{E.\ coli}$ and HC_{10} is shown. The units for all properties are (µg mL^{-1}). Reward settings: $a = 2, b = 2, c = 1, X = 11, Y = 1$.

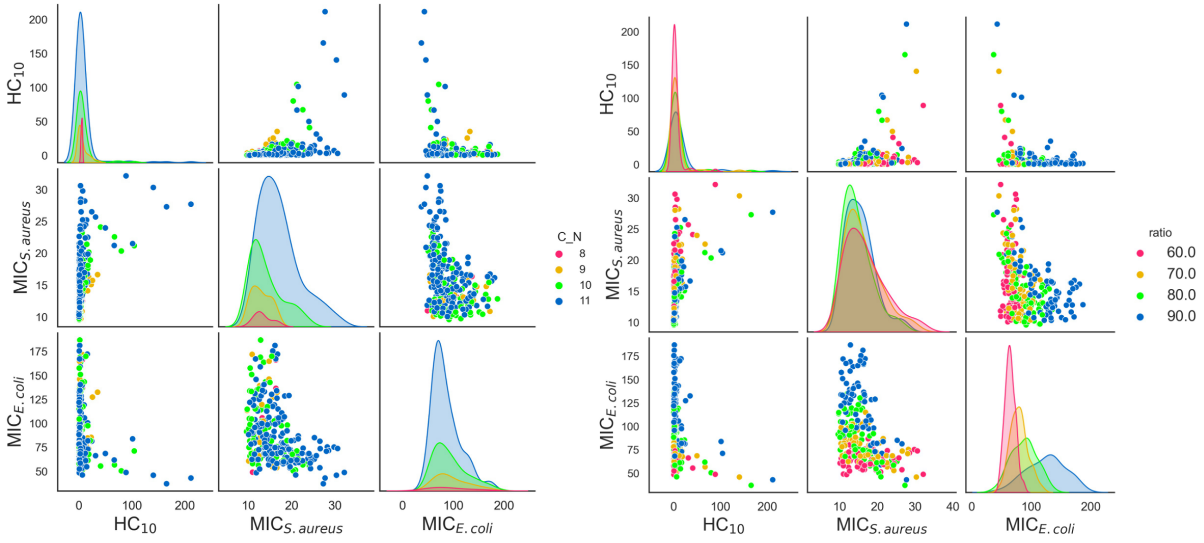

Supplementary Fig. 44 Generation setting: cationic:MM/hydrophobic: $\beta^{2,2,3,3}$-subunit. Property distribution of the predicted value of $\mathrm{MIC}_{S.\ aureus}$, $\mathrm{MIC}_{E.\ coli}$ and HC_{10} is shown. The units for all properties are (µg mL^{-1}). Reward settings: $a = 2, b = 2, c = 1, X = 11, Y = 1$.

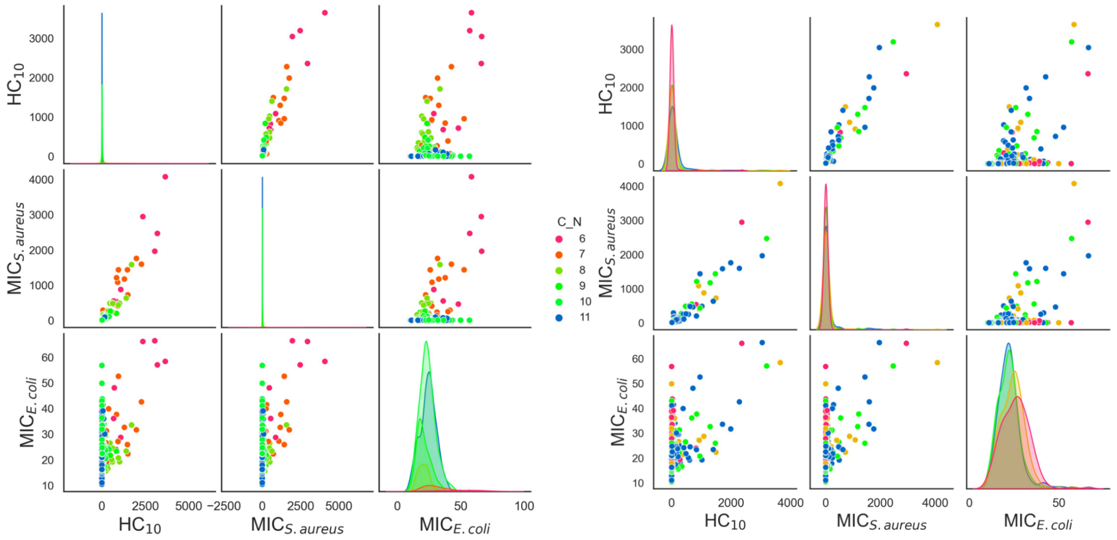

Supplementary Fig. 45 Generation setting: cationic:MM/hydrophobic: $\beta^{2,3}$-subunit cyclic. Property distribution of the predicted value of $\mathrm{MIC}_{S.\ aureus}$, $\mathrm{MIC}_{E.\ coli}$ and HC_{10} is shown. The units for all properties are (μg mL^{-1}). Reward settings: $a = 2, b = 2, c = 1, X = 11, Y = 1$.

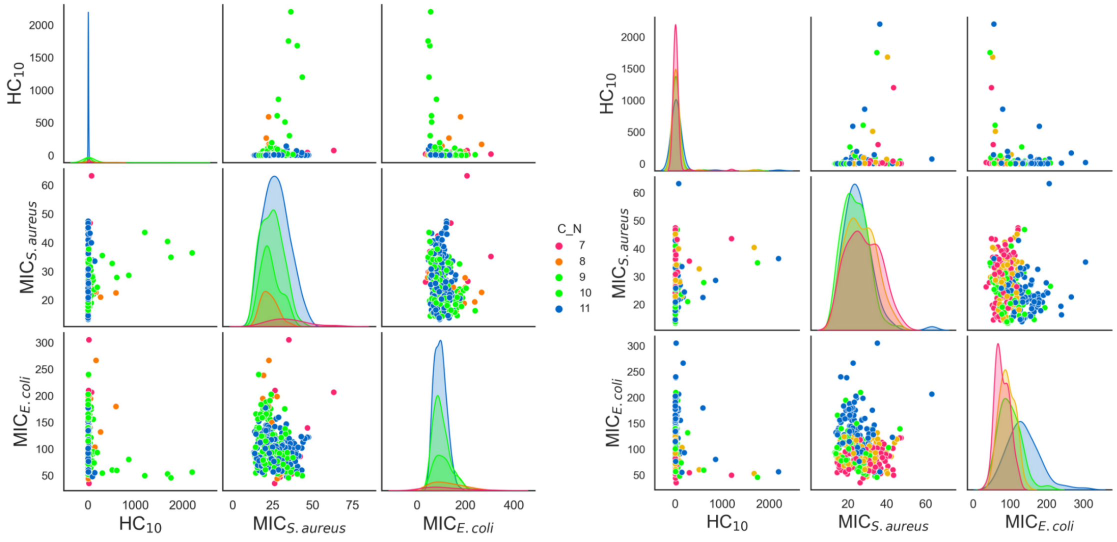

Supplementary Fig. 46 Generation setting: cationic:MM/hydrophobic: $\beta^{3,3}$-subunit. Property distribution of the predicted value of $\mathrm{MIC}_{S.\ aureus}$, $\mathrm{MIC}_{E.\ coli}$ and HC_{10} is shown. The units for all properties are (μg mL^{-1}). Reward settings: $a = 2, b = 2, c = 1, X = 11, Y = 1$.

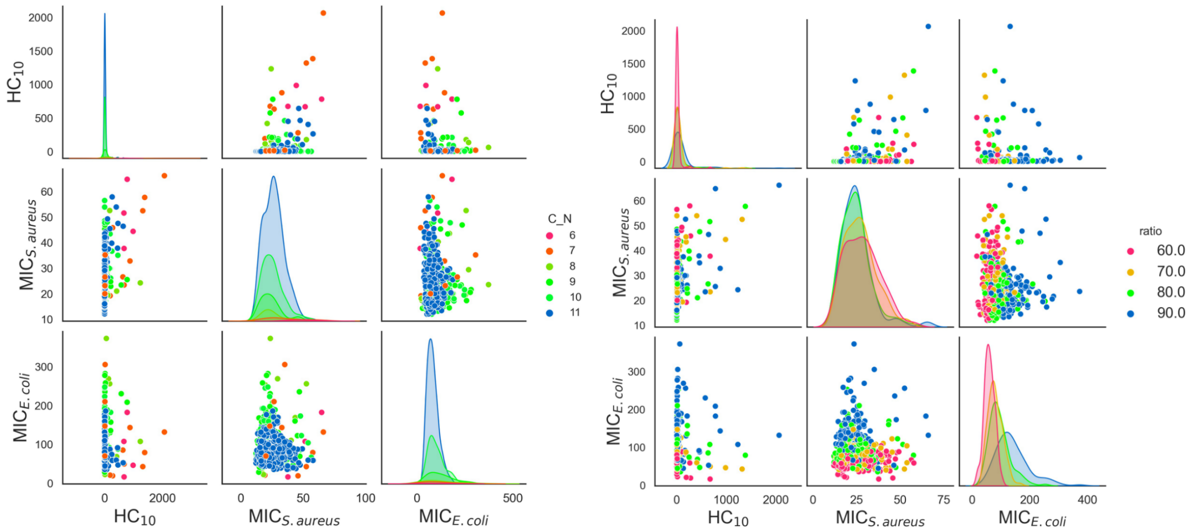

Supplementary Fig. 47 Generation setting: cationic:MM/hydrophobic: $\beta^{3,3}$-subunit cyclic. Property distribution of the predicted value of $\text{MIC}_{S.\ aureus}$, $\text{MIC}_{E.\ coli}$ and HC_{10} is shown. The units for all properties are (µg mL^{-1}). Reward settings: $a = 2, b = 2, c = 1, X = 11, Y = 1$.

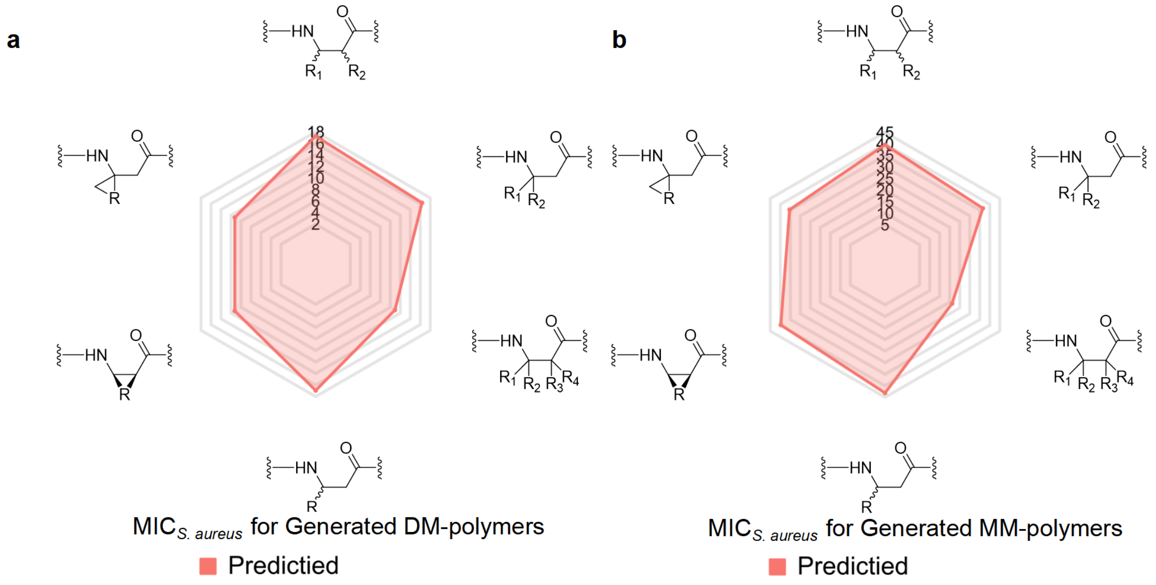

Supplementary Fig. 48 Distribution comparison between generated polymers with different cationic and hydrophobic subunits on $\text{MIC}_{S.aureus}$. a) cationic: DM b) cationic: MM. The unit of the value is µg mL^{-1}.

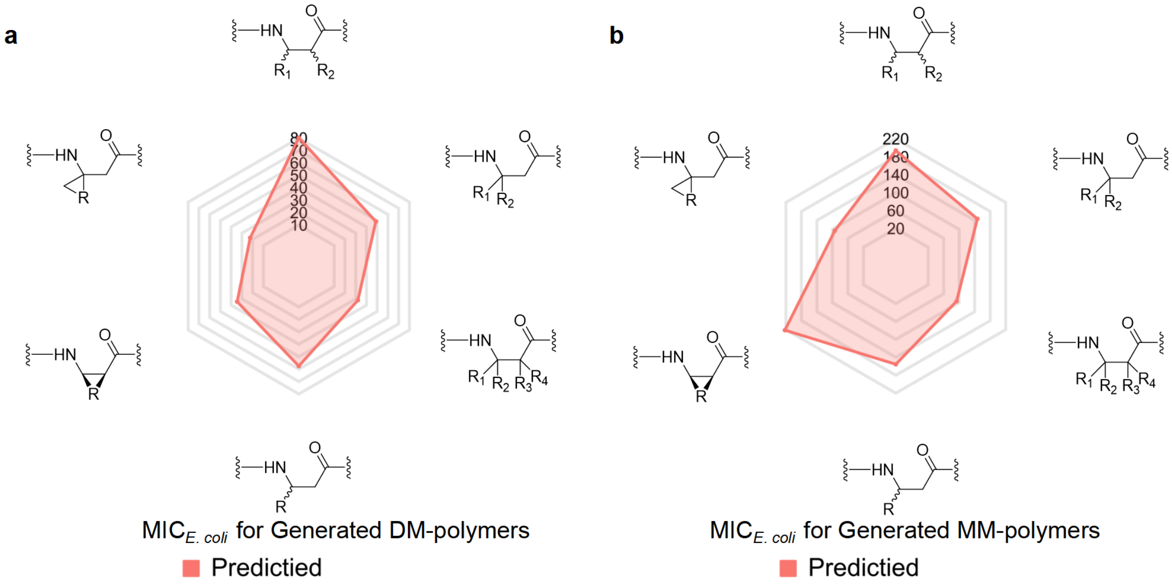

Supplementary Fig. 49 Distribution comparison between generated polymers with different cationic and hydrophobic subunits on MIC$_{E.coli}$. a) cationic: DM b) cationic: MM. The unit of the value is μg mL^{-1}.

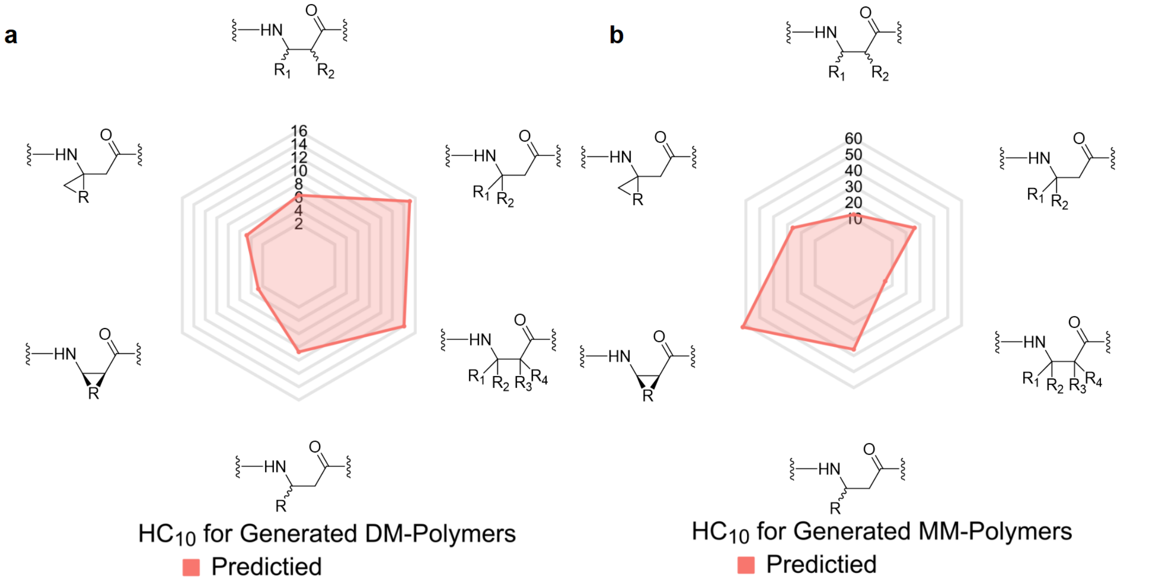

Supplementary Fig. 50 Distribution comparison between generated polymers with different cationic and hydrophobic subunits on HC$_{10}$. a) cationic: DM b) cationic: MM. The unit of the value is μg mL^{-1}.

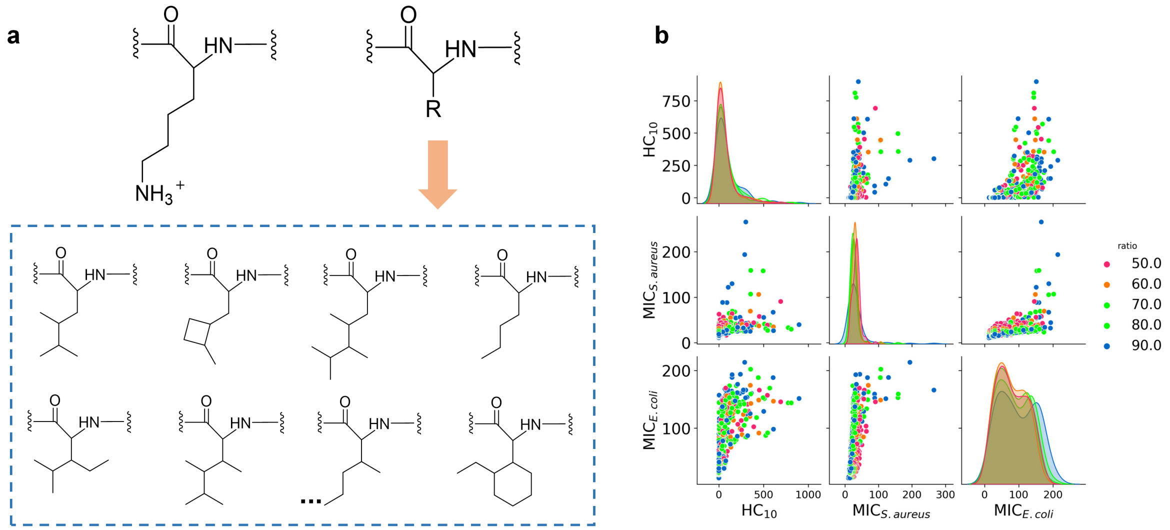

Supplementary Fig. 51 Polymer discovery with α-peptide scaffold. a) Various hydrophobic α-amino acid generated in the discovery process with fixed D,L-Lysine cationic subunit. b) Property distribution of the predicted value of $MIC_{S.aureus}$, $MIC_{E.coli}$ and HC_{10} is shown.The units for all properties are (μg mL^{-1}).

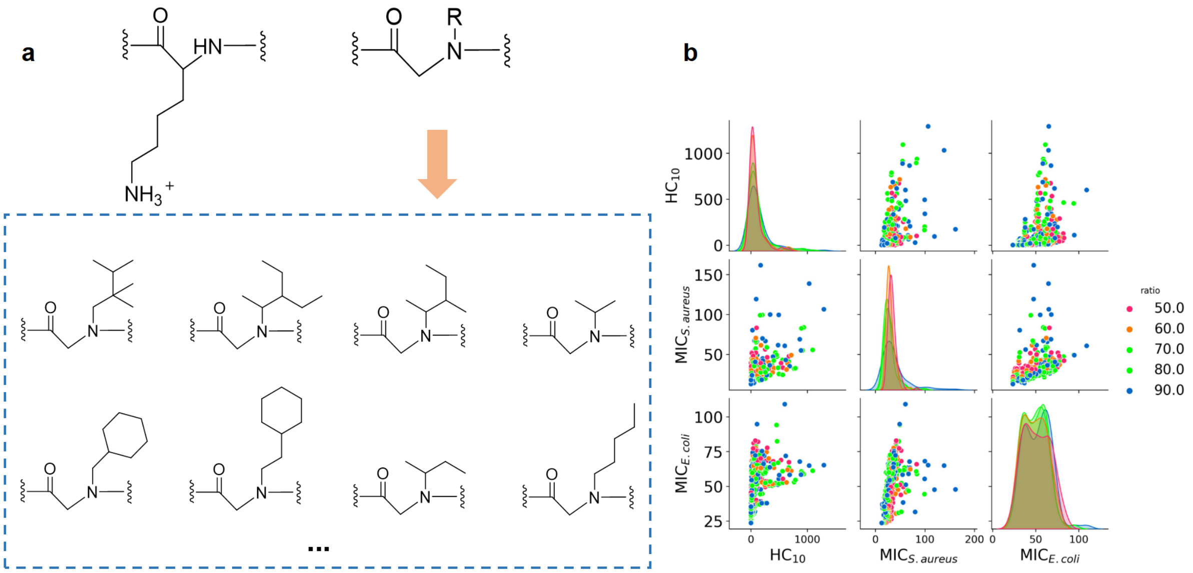

Supplementary Fig. 52 Polymer discovery with α-peptide/peptoid hybrid scaffold with *N*-substituted glycine peptoid. a) Various hydrophobic *N*-substituted glycine subunits generated in the discovery process with fixed D,L-Lysine cationic subunit. b) Property distribution of the predicted value of $MIC_{S.aureus}$, $MIC_{E.coli}$ and HC_{10} is shown. The units for all properties are (µg mL^{-1}).

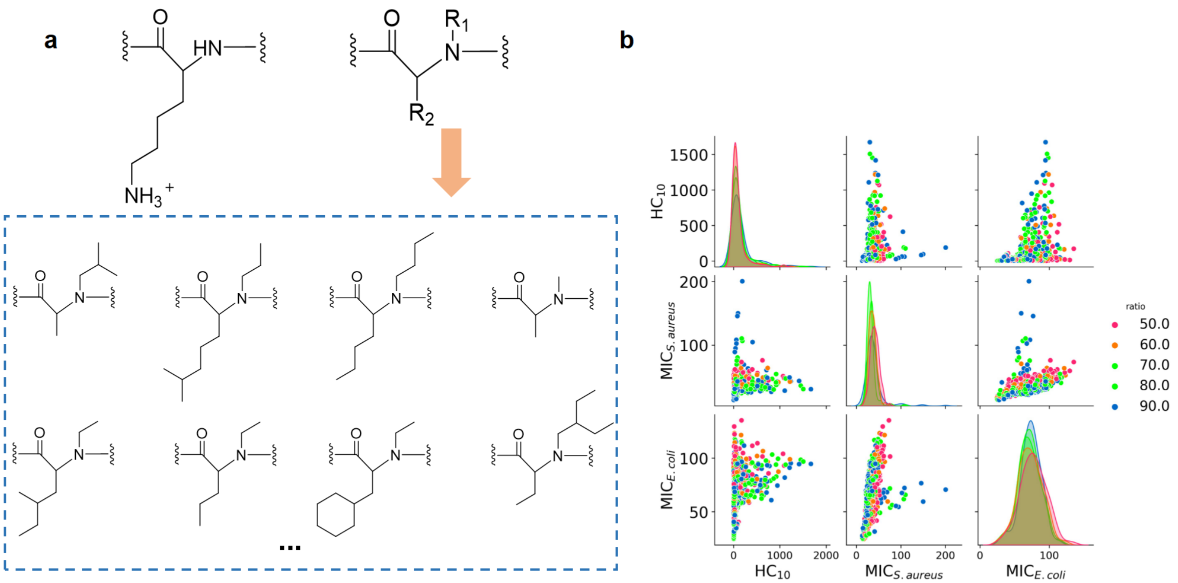

Supplementary Fig. 53 Polymer discovery with α-peptide/peptoid hybrid scaffold with disubstituted peptoid. a) Various hydrophobic *N*-substituted hydrophobic disubstituted subunit generated in the discovery process with fixed D,L-Lysine cationic subunit. b) Property distribution of the predicted value of $\mathrm{MIC}_{S.aureus}$, $\mathrm{MIC}_{E.coli}$ and HC_{10} is shown. The units for all properties are (µg mL^{-1}).

Candidate	Structure	x:y	HC_{10} (µg mL^{-1})	$MIC_{S. aureus}$ (µg mL^{-1})	$MIC_{E. Coli}$ (µg mL^{-1})
Candidate_α_1	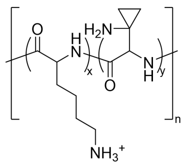	2:8	186.57	28.84	33.08
Candidate_α_ 2	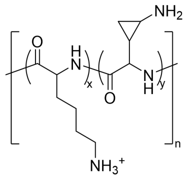	2:8	208.74	31.87	31.76
Candidate_α_ 3	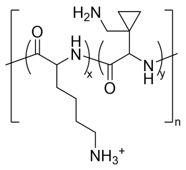	5:5	101.81	18.19	20.67
Candidate_α_ 4		6:4	103.47	20.67	29.85

Supplementary Fig. 54 α**-amino acid subunit optimized structures. The units for all properties are (µg mL^{-1}). We default n to 20 for prediction.**

Candidate	Structure	x:y	HC_{10} (µg mL^{-1})	$MIC_{S. aureus}$ (µg mL^{-1})	$MIC_{E. Coli}$ (µg mL^{-1})
Candidate_α_5	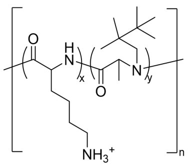	2:8	107.15	36.31	40.43
Candidate_α_ 6	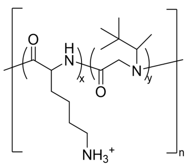	6:4	207.10	21.49	37.84
Candidate_α_ 7		7:3	242.58	19.60	37.65
Candidate_α_ 8	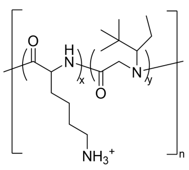	7:3	137.27	19.31	36.80
Candidate_α_ 9		8:2	157.65	17.41	36.91

Supplementary Fig. 55 α**-amino acid subunit optimized structures. The units for all properties are (µg mL^{-1}). We default n to 20 for prediction.**

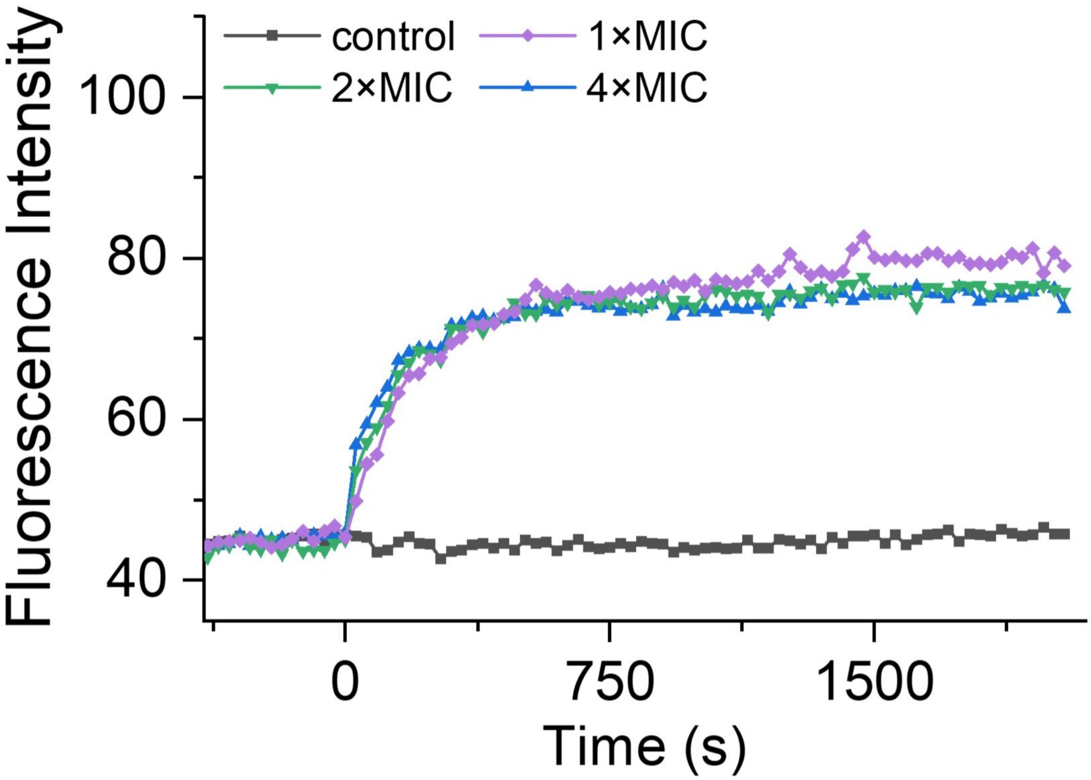

Supplementary Fig. 56 Cytoplasmic membrane permeabilization of $(DM_{0.8}\textit{i}Pen_{0.2})_{20}$ against *E. coli* R19

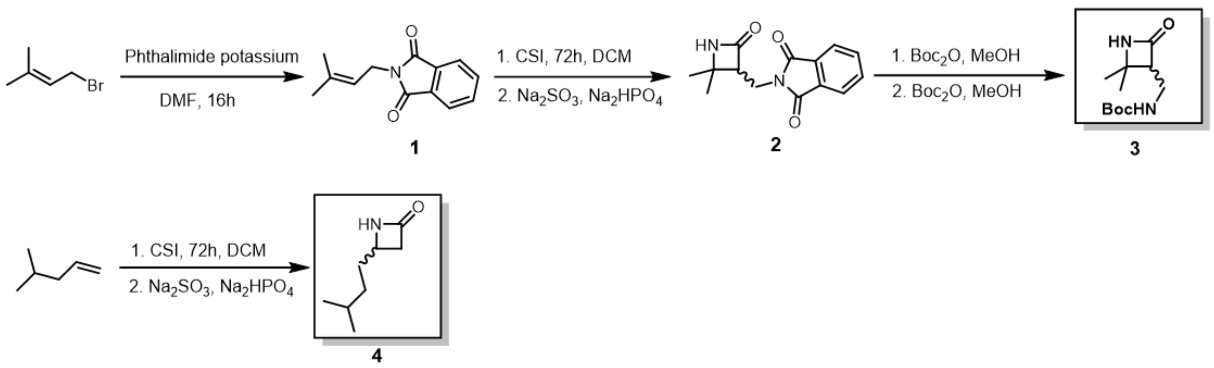

Supplementary Fig. 57 The synthetic routes to beta-lactam monomers.

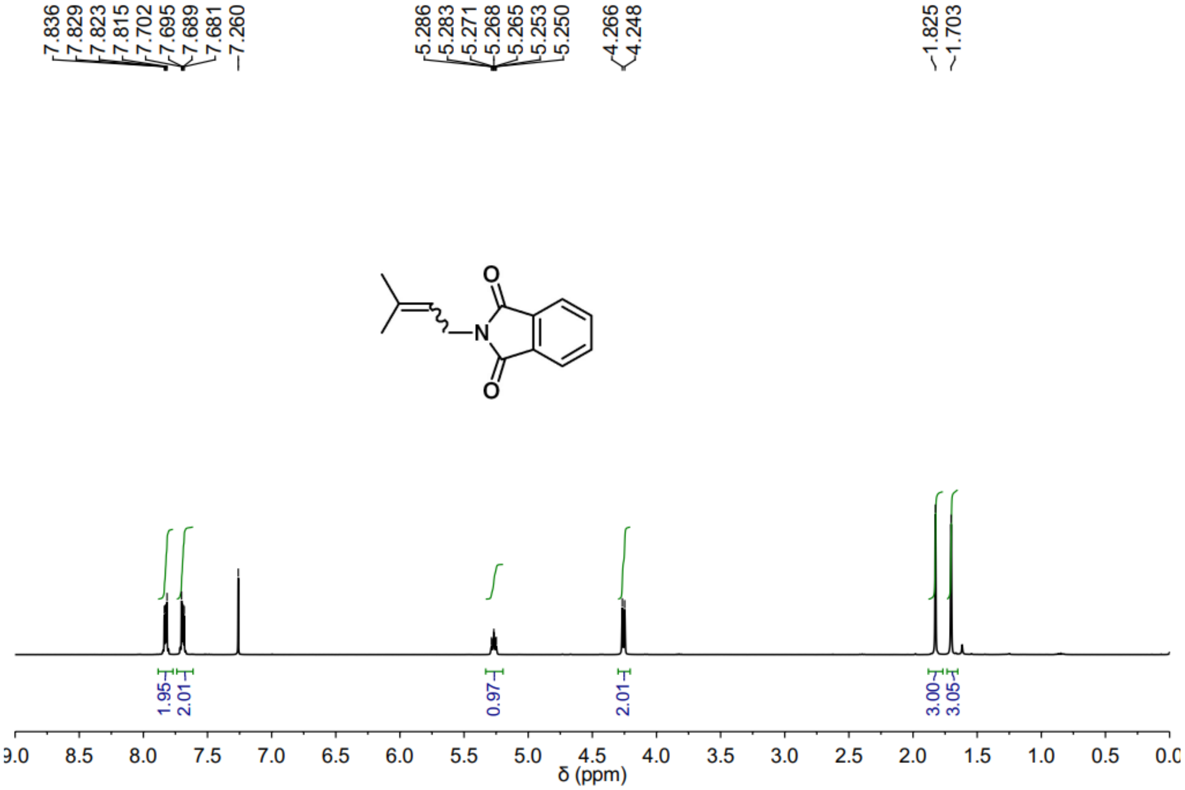

Supplementary Fig. 58 ^{1}H NMR spectrum of intermediate compound in $CDCl_3$, 400 MHz.

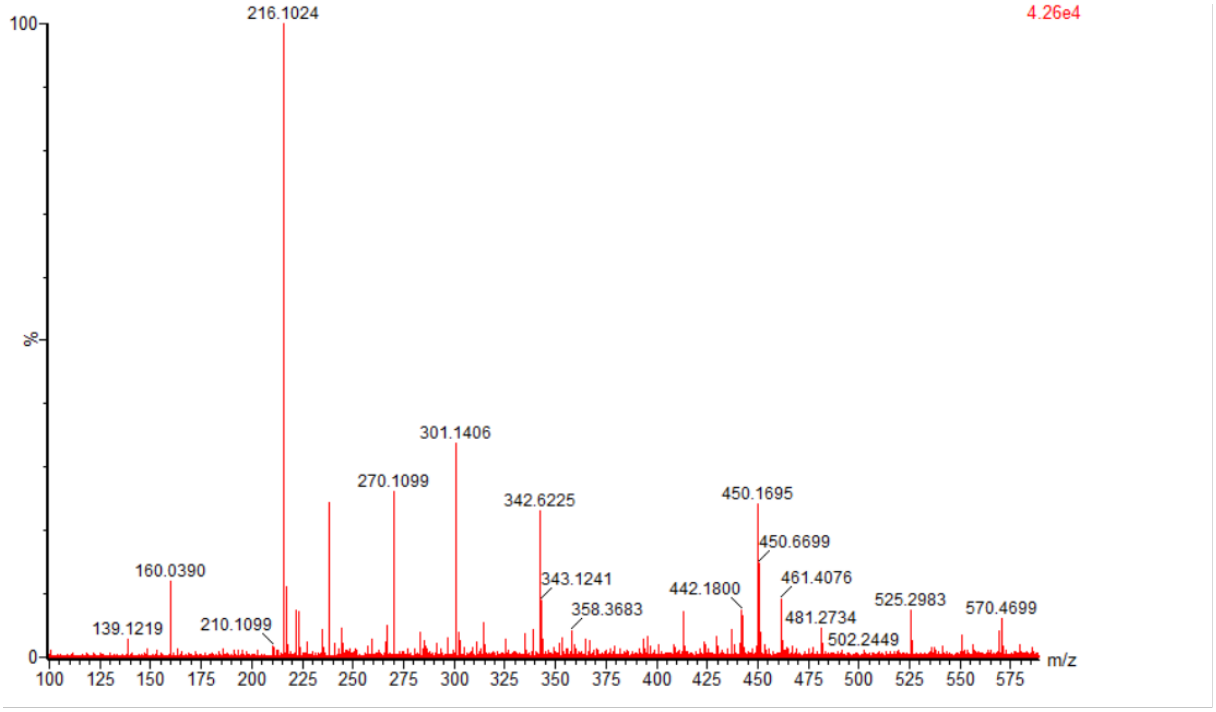

Supplementary Fig. 59 HRESI-MS spectrum of intermediate compound.

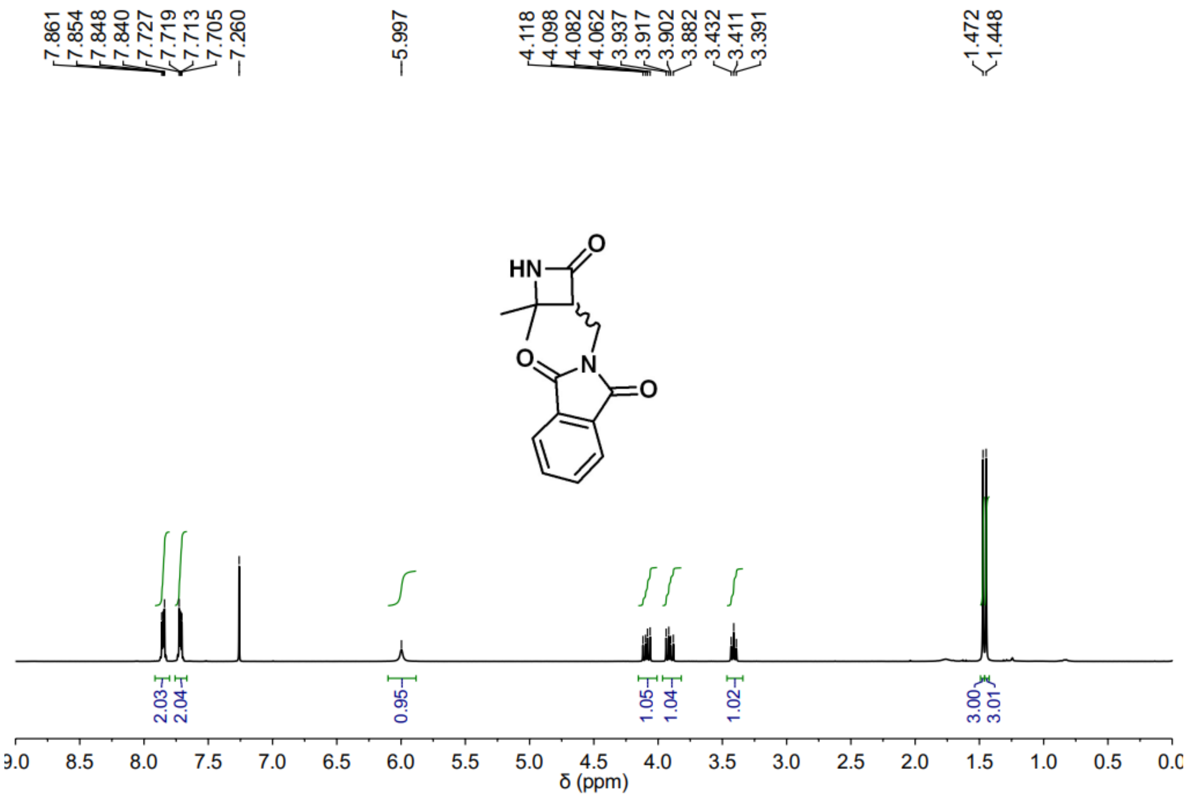

Supplementary Fig. 60 ^{1}H NMR spectrum of Maleimide-protected DM in $CDCl_3$, 400 MHz.

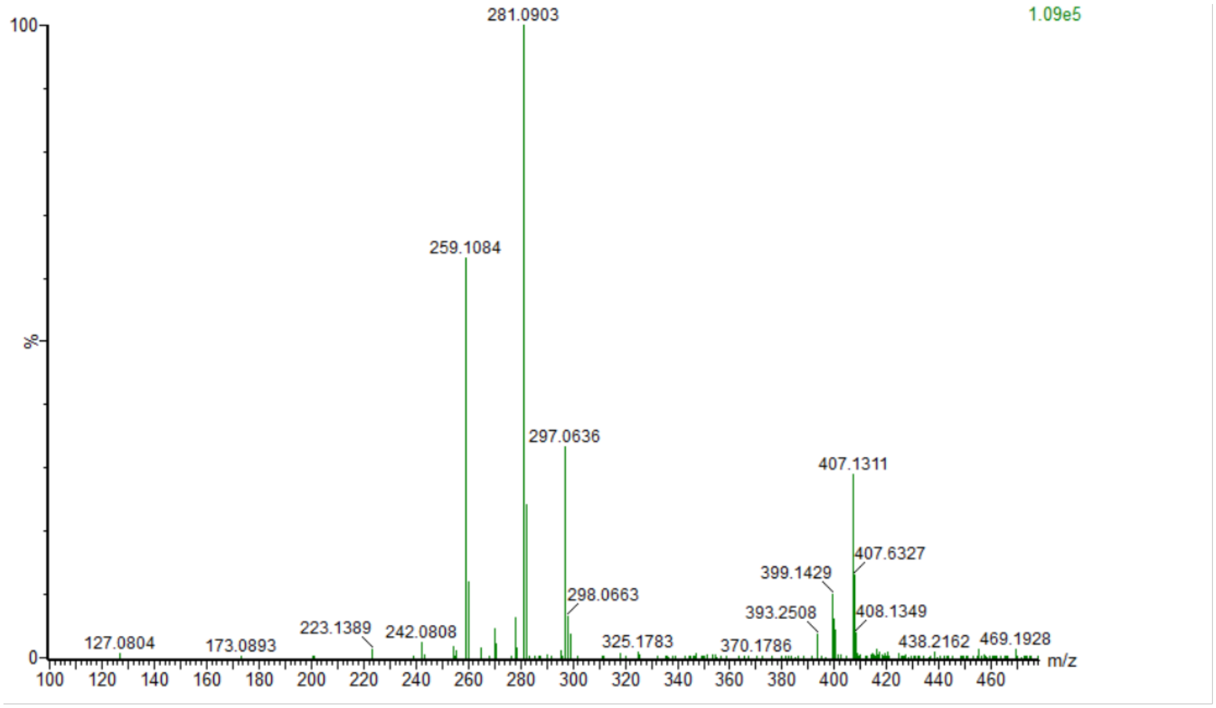

Supplementary Fig. 61 HRESI-MS spectrum of Maleimide-protected DM.

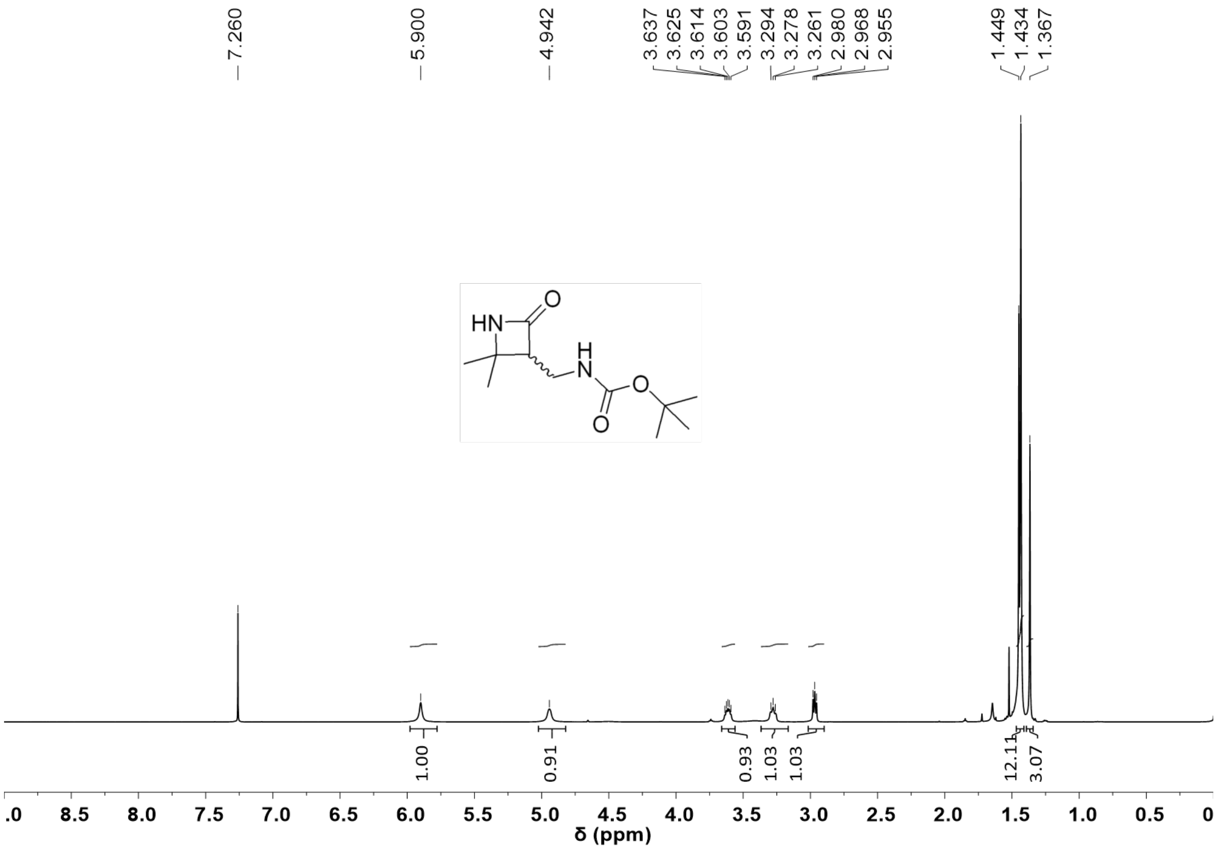

Supplementary Fig. 62 ^{1}H NMR spectrum of β-lactam DM in $CDCl_3$, 400 MHz.

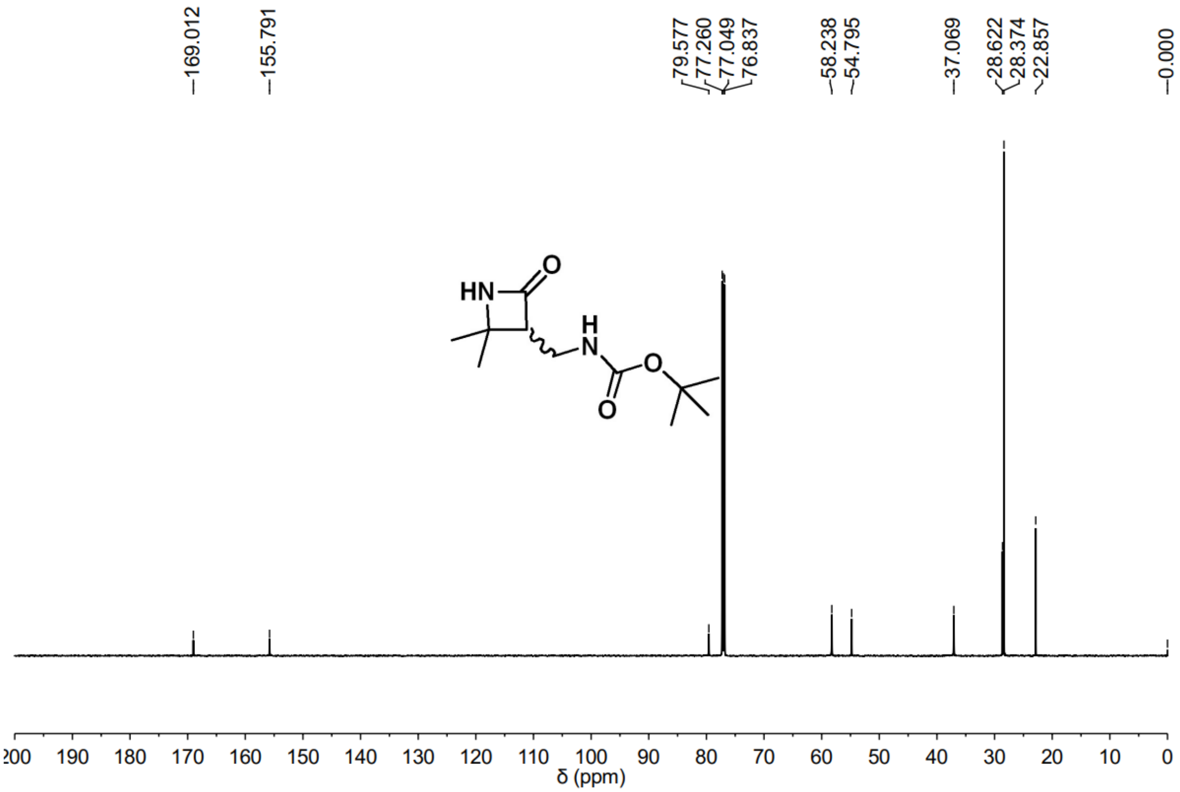

Supplementary Fig. 63 ^{13}C NMR spectrum of β-lactam DM in $CDCl_3$, 100 MHz.

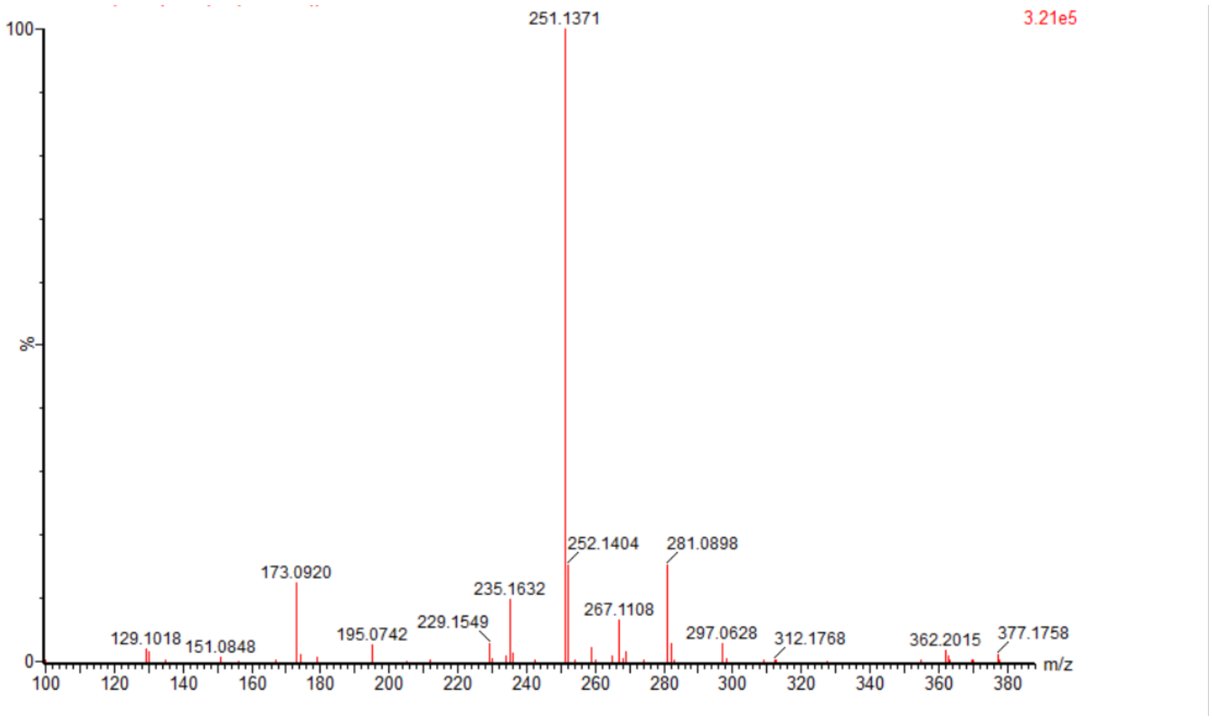

Supplementary Fig. 64 HRESI-MS spectrum of β-lactam DM.

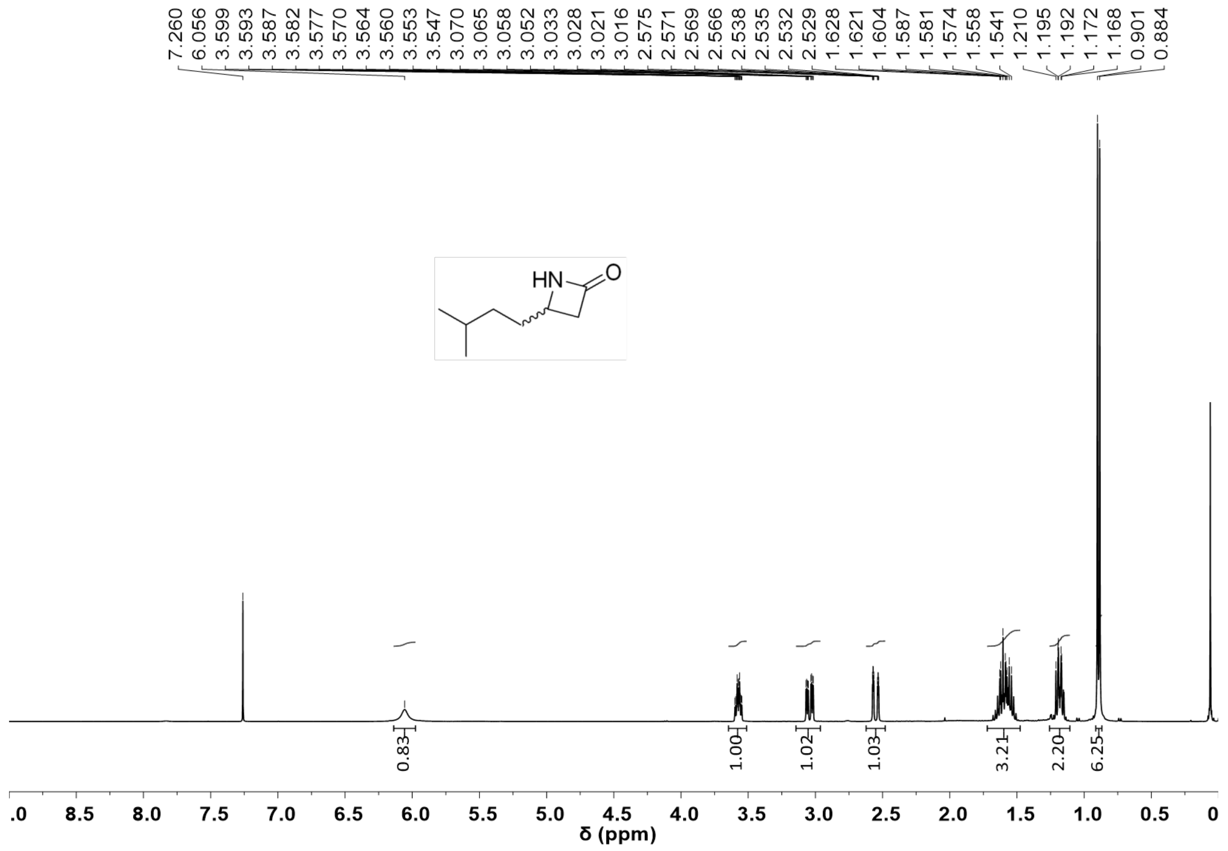

Supplementary Fig. 65 ^{1}H NMR spectrum of β-lactam *i*Pen in $CDCl_3$, 400 MHz.

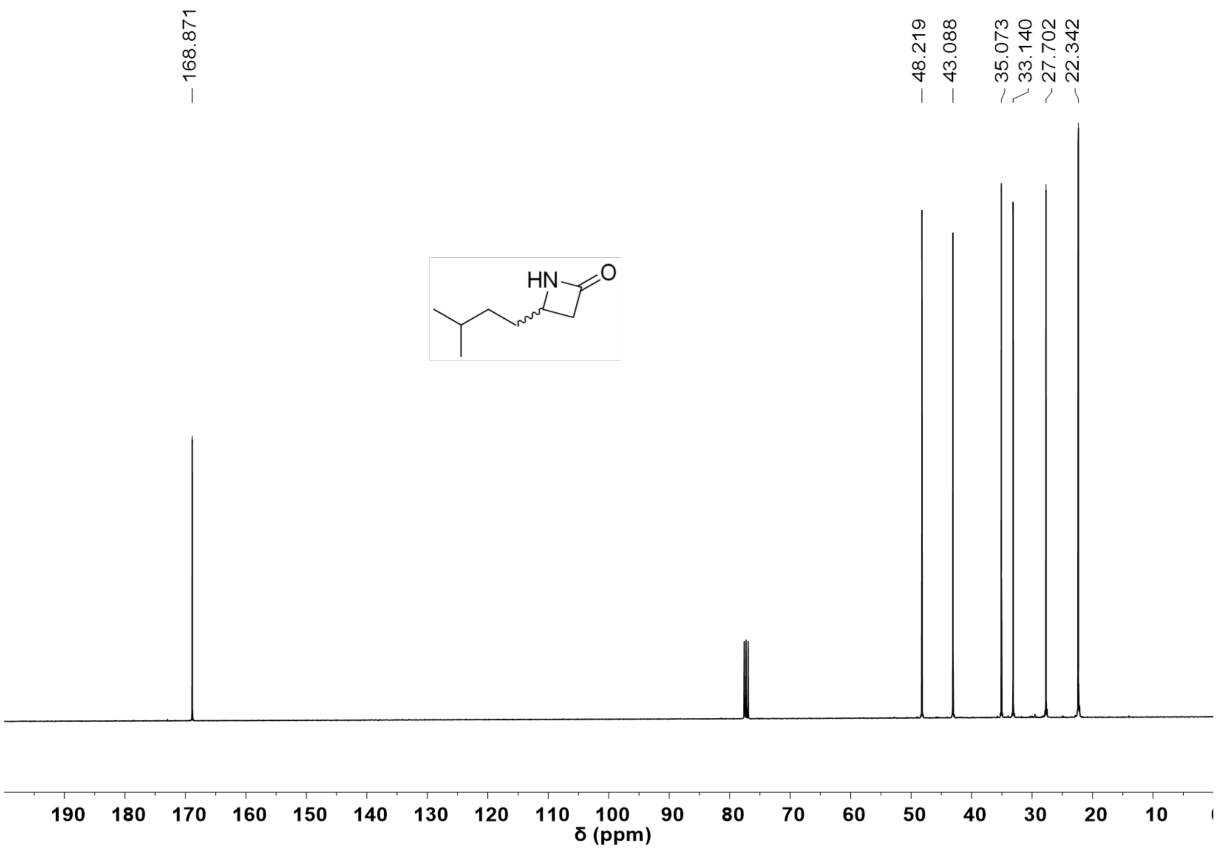

Supplementary Fig. 66 ^{13}C NMR spectrum of β-lactam *i*Pen in $CDCl_3$, 100 MHz.

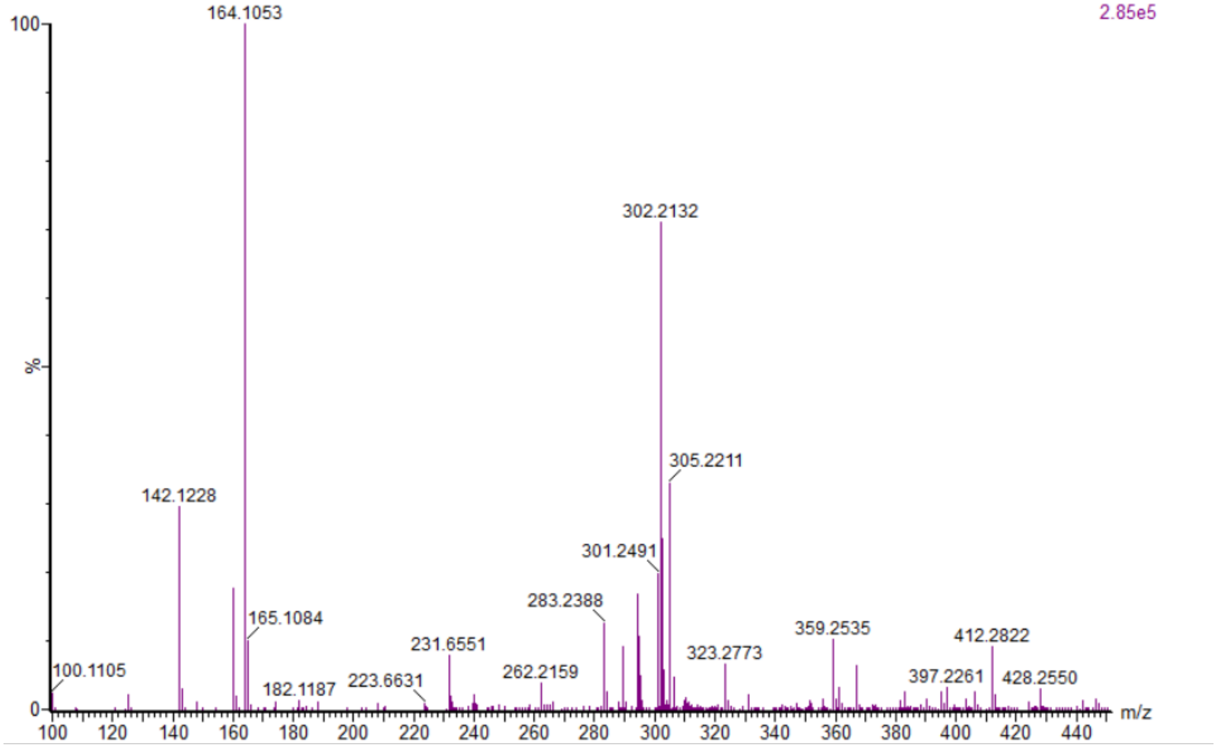

Supplementary Fig. 67 HRESI-MS spectrum of β-lactam *i*Pen.

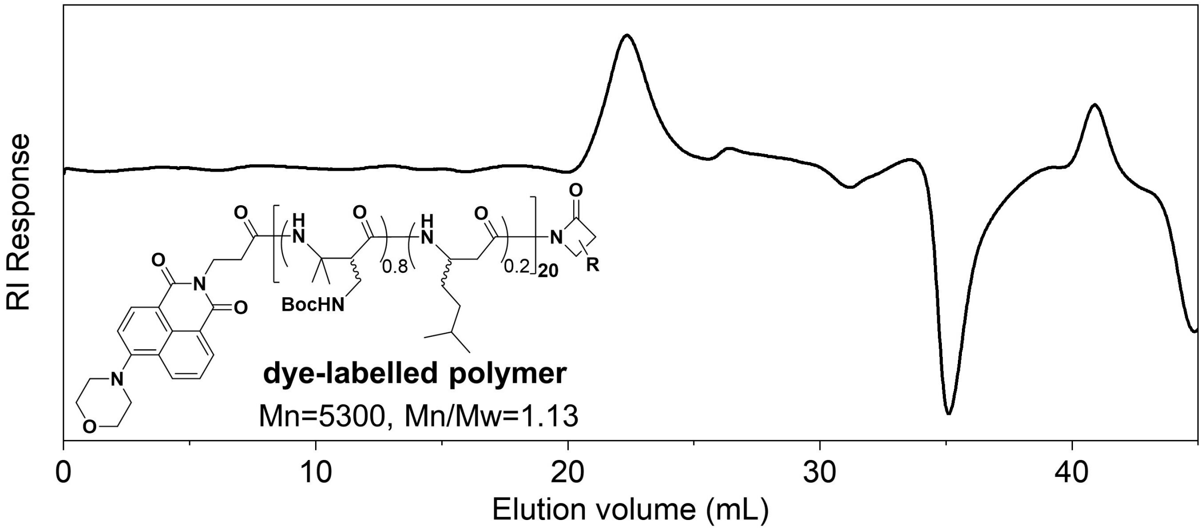

Supplementary Fig. 68 GPC trace of N-Boc protected dye-labelled polymer using DMF as the mobile phase

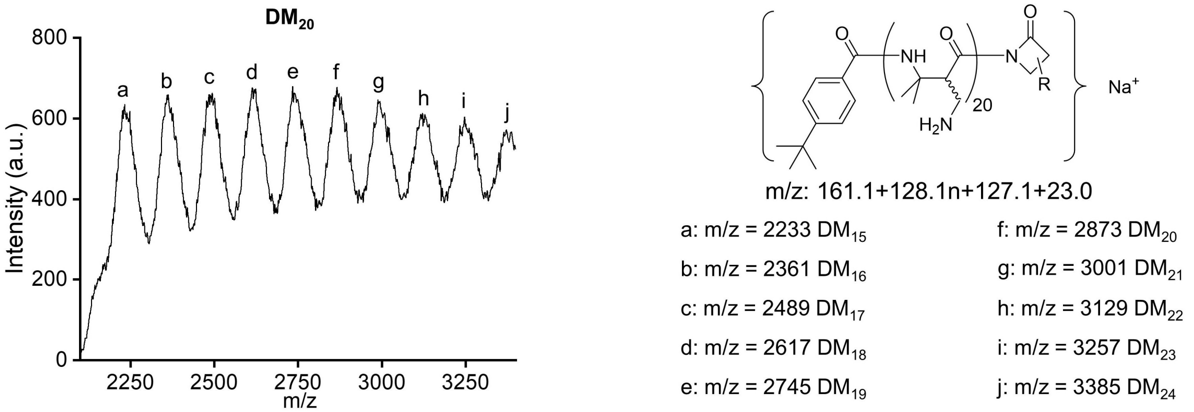

Supplementary Fig. 69 MALDI-TOF MS characterization of antibacterial β-amino acid polymer DM$_{20}$.

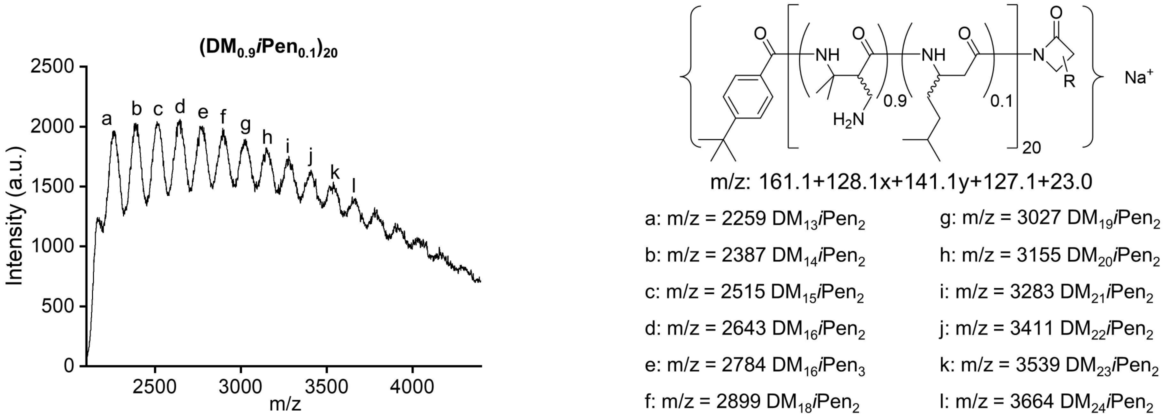

Supplementary Fig. 70 MALDI-TOF MS characterization of antibacterial β-amino acid polymer (DM$_{0.9}$*i*Pen$_{0.1}$)$_{20}$.

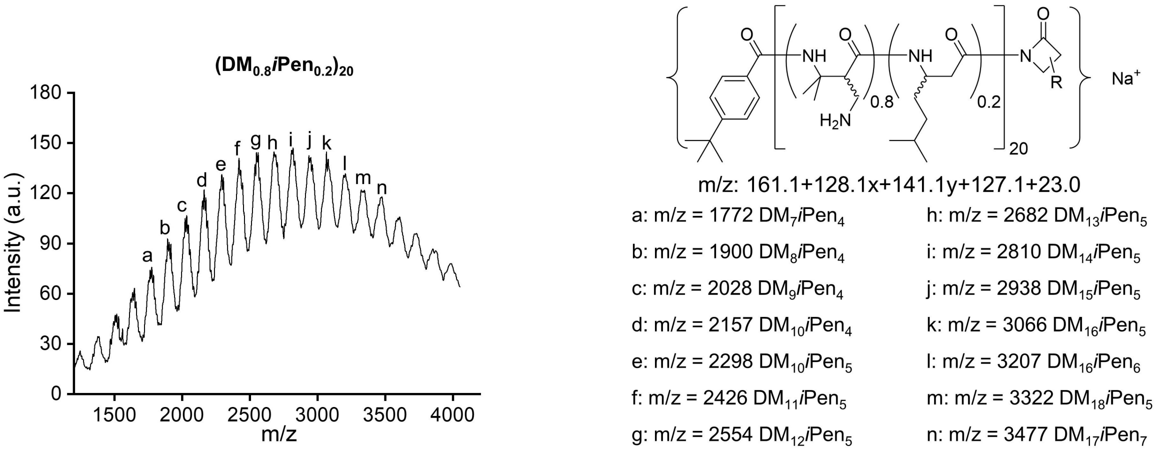

Supplementary Fig. 71 MALDI-TOF MS characterization of antibacterial β-amino acid polymer ($DM_{0.8}$*i*$Pen_{0.2}$)$_{20}$.

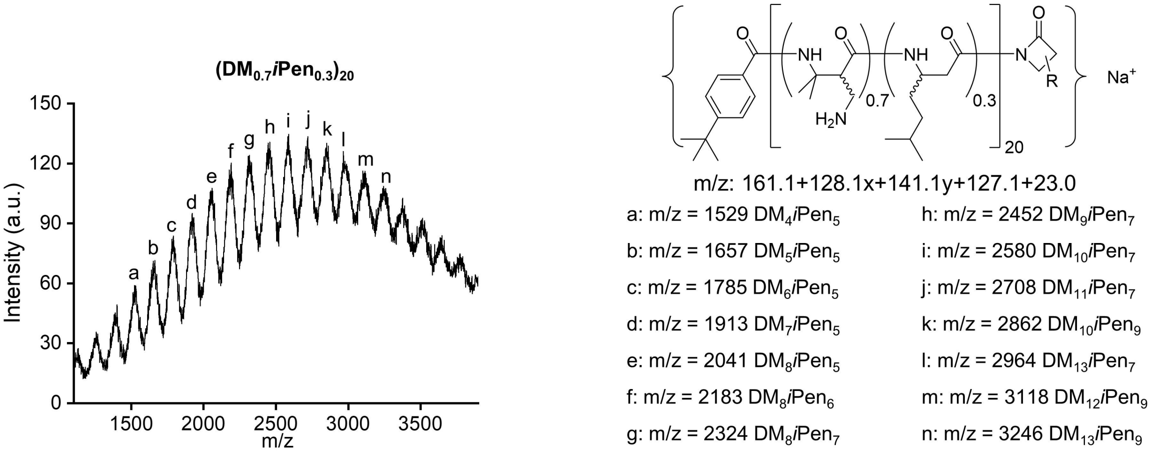

Supplementary Fig. 72 MALDI-TOF MS characterization of antibacterial β-amino acid polymer ($DM_{0.7}$*i*$Pen_{0.3}$)$_{20}$.

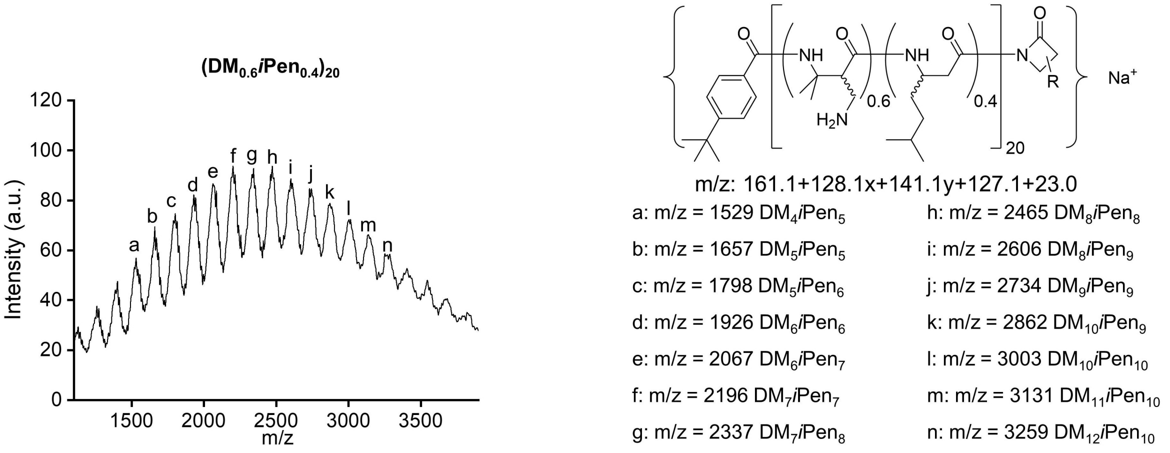

Supplementary Fig. 73 MALDI-TOF MS characterization of antibacterial β-amino acid polymer (DM$_{0.6}$*i* Pen$_{0.4}$)$_{20}$.

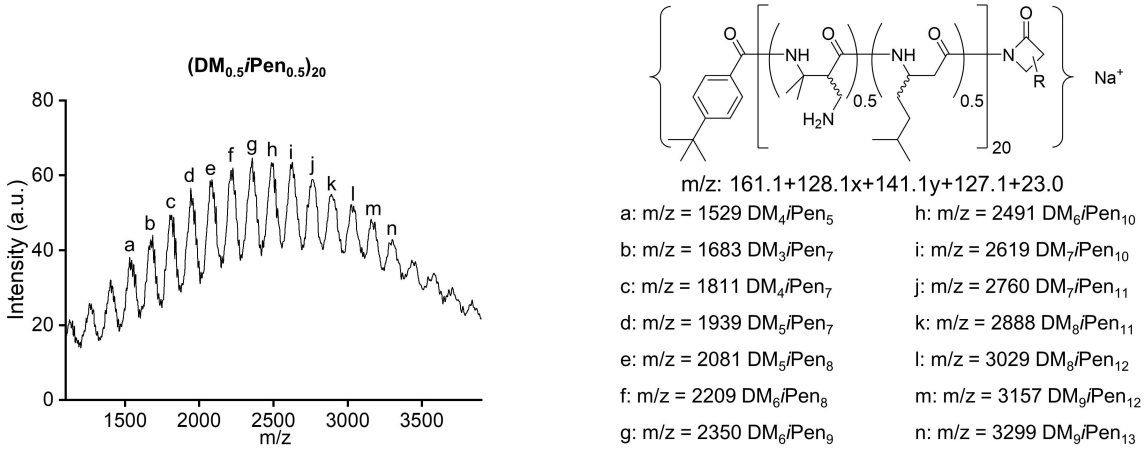

Supplementary Fig. 74 MALDI-TOF MS characterization of antibacterial β-amino acid polymer (DM$_{0.5}$*i*Pen$_{0.5}$)$_{20}$.

Supplementary Table 1. Scaffold sequence. "[*]" means the substitution token.

No	scaffold SMILES sequence	No	scaffold SMILES sequence
1	NC(CC=O)[*]	8	NC(C([*])(C=O)[*])[*]
2	NCC(C=O)[*]	9	NC1[*]C1C=O
3	NC(C(C=O)[*])[*]	10	NC1([*]C1)CC=O
4	NC([*])(CC=O)[*]	11	NCC1([*]C1)C=O
5	NCC([*])(C=O)[*]		
6	NC([*])(C(C=O)[*])[*]		
7	NC([*])(C([*])(C=O)[*])[*]		

Supplementary Table 2. Summary of the hyper-parameter ranges for the predictive model.

Hyper-Parameters	Descriptions	Range
Batch size	batch size used during the training of the model	16
Warmup Epochs	number of epochs during which the learning rate changes	5
Epoch	number of epochs for training	100
Layer number (GNN)	the number of GNN layers	5
Layer number (GRU)	the number of GRU layers	3
Layer number (FFN)	the number of Feed-Forward Network layers	5
Layer number (Trans)	the number of Transformer layers	6
Attention heads number	number of self-attention heads in Transformer	8
GNN Hidden	dimensionality of hidden layers in GNN	256
GRU Hidden	dimensionality of hidden layers in GRU	256
FNN Hidden	dimensionality of hidden layers in FNN	256
Transformer Hidden	dimensionality of hidden layers in Transformer	256
Initial Learning rate	initial learning rate of Noam learning rate scheduler	1e-4
Max Learning rate	max learning rate of Noam learning rate scheduler	2e-4
Final Learning rate	learning rate of Noam learning rate scheduler	1e-4

Supplementary Table 3. Quantitative predictive results on the value of $\mathrm{MIC}_{S.\ aureus}$, $\mathrm{MIC}_{E.\ coli}$ and HC_{10} with different polymer representations. Mean and standard deviation (in brackets) of R2 values are reported (n=10). Results in bold means the best results.

Task	R2 of Predicted $\mathrm{MIC}_{S.\ aureus}$, $\mathrm{MIC}_{E.\ coli}$ and HC_{10}		
Method	$\mathrm{MIC}_{S.\ aureus}$	$\mathrm{MIC}_{E.\ coli}$	HC_{10}
GBDT	0.673(0.094)	0.537(0.096)	0.834(0.071)
RF	0.666(0.087)	0.517(0.072)	0.810(0.064)
XGB	0.607(0.112)	0.536(0.064)	0.794(0.084)
Adaboost	0.591(0.132)	0.517(0.139)	0.794(0.064)
Morgan	0.503(0.053)	0.387(0.040)	0.827(0.016)
Graph	0.569(0.066)	0.413(0.034)	0.834(0.080)
Sequence	0.423(0.045)	0.419(0.043)	0.493(0.039)
Descriptors_Init	0.415(0.098)	0.245(0.064)	0.650(0.081)
Descriptors_Var	0.478(0.056)	0.299(0.111)	0.691(0.097)
Descriptors_Cor	0.472(0.027)	0.371(0.089)	0.722(0.055)
Descriptors_Opt	0.606(0.036)	0.415(0.105)	0.852(0.031)
Graph + Descriptors_Opt	0.673(0.064)	0.506(0.061)	0.863(0.067)
Sequence+Descriptors_Opt	0.690(0.043)	0.522(0.062)	0886(0.053)
Seq + Graph + Descriptors_Opt	**0.697(0.046)**	**0.556(0.051)**	**0.900(0.040)**

Supplementary Table 4. Quantitative predictive results on the value of $MIC_{S. aureus}$, $MIC_{E. coli}$ and HC_{10} with different polymer representations. Mean and standard deviation (in brackets) of RMSE values are reported (n=10). Results in bold means the best results.

Task	RMSE of Predicted $MIC_{S. aureus}$, $MIC_{E. coli}$ and HC_{10}		
Method	$MIC_{S. aureus}$	$MIC_{E. coli}$	HC_{10}
GBDT	0.808(0.078)	1.314(0.106)	1.559(0.238)
RF	0.855(0.091)	1.274(0.102)	1.609(0.324)
XGB	0.853(0.091)	1.281(0.119)	1.440(0.291)
Adaboost	0.865(0.089)	1.308(0.191)	1.623(0.244)
Morgan	1.025(0.053)	1.268(0.041)	1.341(0.062)
Graph	0.953(0.072)	1.240(0.036)	1.280(0.299)
Sequence	1.105(0.042)	1.233(0.046)	2.298(0.089)
Descriptors_Init	1.110(0.091)	1.407(0.059)	1.896(0.175)
Descriptors_Var	1.051(0.056)	1.352(0.105)	1.771(0.264)
Descriptors_Cor	1.058(0.027)	1.281(0.090)	1.696(0.163)
Descriptors_Opt	0.913(0.041)	1.208(0.047)	1.236(0.142)
Graph + Descriptors_Opt	0.829(0.082)	1.136(0.073)	1.161(0.282)
Sequence+Descriptors_Opt	0.809(0.058)	1.135(0.054)	1.063(0.253)
Seq + Graph + Descriptors_Opt	**0.799(0.062)**	**1.122(0.081)**	**0.990(0.196)**

Supplementary Table 5. Summary of the hyper-parameter ranges for the generative model.

Hyper-Parameters	Descriptions	Range
Batch size (pre-training)	batch size used during the pre-training process	256
Batch size (Reinforcement learning)	batch size used during the molecule sampling process in RL	30
Epoch	number of epochs for training	450
Layer number	the number of GRU encoder-decorator layers	3
Model Hidden	dimensionality of hidden layers	256
Learning rate (pre-training)	Learning rate for the agent pre-training	1e-3
Learning rate (Reinforcement learning)	Learning rate for fine-tuning the reinforcement agent	1e-9

Supplementary Table 6. DM/β^3-subunit structures. The units for all properties are (µg mL^{-1}). We default n to 20 for prediction.

Polymer	Structure	x	y	HC_{10}	$MIC_{S. aureus}$	$MIC_{E. coli}$
Polymer_1	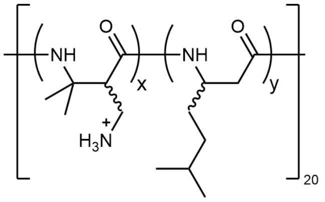	4	6	22.92	19.26	17.33
		5	5	39.92	15.77	16.55
		6	4	71.26	13.35	16.89
		7	3	112.64	11.26	20.70
		8	2	156.89	10.18	24.05
		9	1	217.33	9.90	39.21
Polymer_2	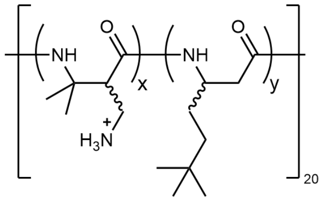	4	6	44.55	24.26	31.72
		5	5	69.49	21.18	21.33
		6	4	92.24	17.95	15.91
		7	3	134.65	15.53	14.68
		8	2	167.70	13.97	15.23
		9	1	214.80	12.78	20.04
Polymer_3	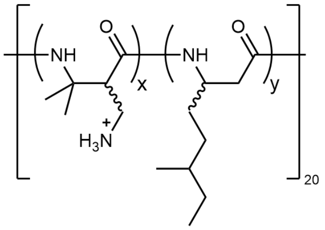	4	6	3.28	18.15	40.31
		5	5	5.64	15.61	25.13
		6	4	9.87	13.02	16.46
		7	3	23.13	10.80	15.62
		8	2	49.25	9.43	21.33
		9	1	119.64	8.31	24.94
Polymer_4	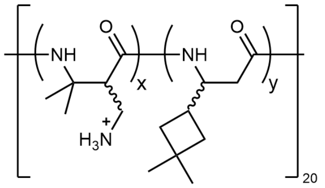	4	6	17.53	20.47	14.91
		5	5	31.02	17.79	12.25
		6	4	41.29	15.02	11.77
		7	3	64.30	13.34	13.15
		8	2	86.59	12.18	13.97
		9	1	117.30	11.65	19.47
Polymer_5	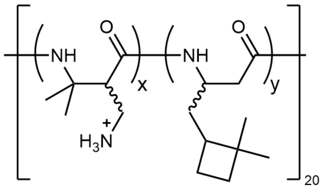	4	6	15.48	18.79	19.34
		5	5	28.43	16.56	14.84
		6	4	39.65	13.82	13.56
		7	3	63.01	12.02	14.14
		8	2	93.23	10.46	14.43
		9	1	131.47	9.93	20.52

Supplementary Table 7. DM/β^3-subunit structures. The units for all properties are (µg mL^{-1}). We default n to 20 for prediction.

Polymer	Structure	x	y	HC_{10}	$MIC_{S.\ aureus}$	$MIC_{E.\ coli}$
Polymer_6	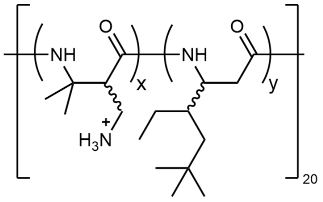	4	6	21.18	27.46	46.78
		5	5	32.51	24.39	32.60
		6	4	45.15	21.13	26.91
		7	3	66.24	15.50	23.29
		8	2	93.54	16.33	19.90
		9	1	131.99	21.31	21.31
Polymer_7	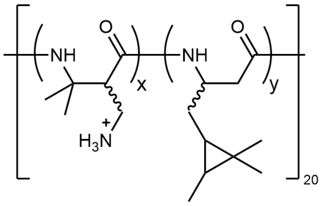	4	6	39.90	18.72	23.52
		5	5	61.97	17.30	21.22
		6	4	78.67	15.58	21.68
		7	3	115.40	14.38	21.10
		8	2	154.85	13.45	20.90
		9	1	211.73	12.85	25.31
Polymer_8	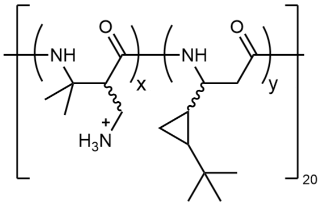	4	6	30.28	18.92	18.90
		5	5	44.64	17.74	16.38
		6	4	58.12	15.85	16.14
		7	3	86.07	14.39	15.76
		8	2	116.13	13.32	15.36
		9	1	164.65	12.73	18.78
Polymer_9	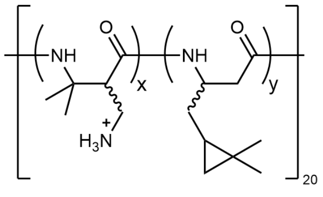	4	6	16.69	19.79	17.62
		5	5	31.28	18.16	14.63
		6	4	45.59	16.67	15.17
		7	3	67.70	15.31	15.95
		8	2	87.02	14.00	16.94
		9	1	127.93	13.81	23.81
Polymer_10	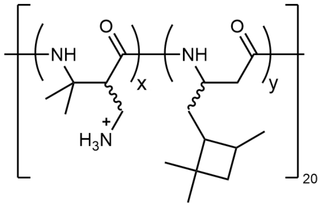	4	6	14.31	28.29	32.05
		5	5	23.38	16.47	25.01
		6	4	31.90	14.08	21.72
		7	3	50.95	12.26	19.50
		8	2	72.51	11.09	18.26
		9	1	107.00	10.43	21.41

Supplementary Table 8. DM/β^3-subunit structures. The units for all properties are (µg mL^{-1}). We default n to 20 for prediction.

Polymer	Structure	x	y	HC_{10}	$MIC_{S. aureus}$	$MIC_{E. coli}$
Polymer_11	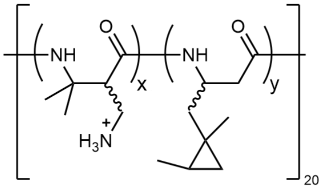	4	6	12.33	17.02	17.74
		5	5	22.38	15.44	14.40
		6	4	36.03	13.83	14.65
		7	3	64.34	12.72	15.02
		8	2	88.45	11.65	16.99
		9	1	120.83	11.21	24.01
Polymer_12	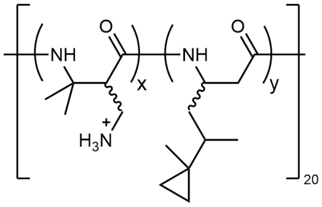	4	6	9.41	14.08	17.05
		5	5	17.97	12.93	14.55
		6	4	29.41	11.38	15.06
		7	3	52.06	10.50	16.11
		8	2	79.22	9.79	19.32
		9	1	110.06	9.32	25.19
Polymer_13	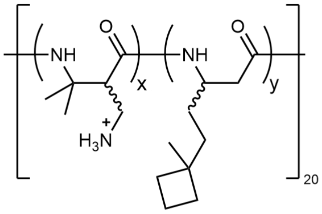	4	6	14.05	18.00	13.59
		5	5	25.54	15.61	10.11
		6	4	37.24	13.23	9.31
		7	3	66.09	11.27	10.99
		8	2	99.62	9.92	14.57
		9	1	133.11	9.39	22.82
Polymer_14	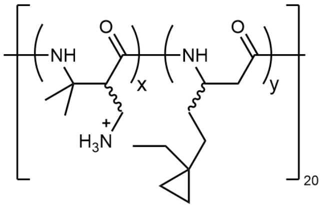	4	6	10.15	16.81	16.17
		5	5	18.95	15.27	12.89
		6	4	28.76	13.62	12.76
		7	3	54.91	12.31	14.48
		8	2	80.60	11.05	17.15
		9	1	112.25	10.66	25.37
Polymer_15	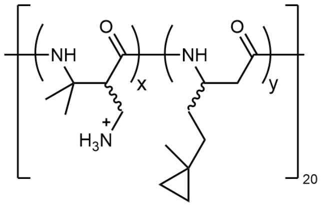	4	6	87.41	18.56	16.30
		5	5	127.70	16.57	13.12
		6	4	153.08	14.63	12.45
		7	3	228.38	13.30	13.43
		8	2	287.91	12.09	16.25
		9	1	401.57	12.27	24.97

Supplementary Table 9. DM/$\beta^{3,3}$-subunit cyclic structures. The units for all properties are (µg mL^{-1}). We default n to 20 for prediction.

Polymer	Structure	x	y	HC_{10}	$MIC_{S. aureus}$	$MIC_{E. coli}$
Polymer_1	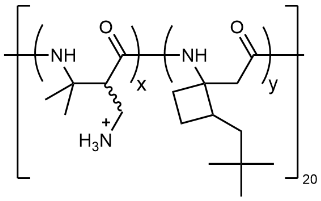	4	6	96.98	28.87	21.82
		5	5	131.90	25.97	17.15
		6	4	151.02	22.98	15.34
		7	3	196.57	20.73	13.94
		8	2	238.32	18.66	12.13
		9	1	305.03	17.22	13.19
Polymer_2	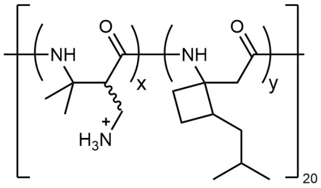	4	6	23.60	22.20	33.98
		5	5	40.40	19.37	24.68
		6	4	52.58	16.58	20.16
		7	3	78.62	14.24	18.52
		8	2	109.05	12.50	17.09
		9	1	150.11	11.74	21.11
Polymer_3	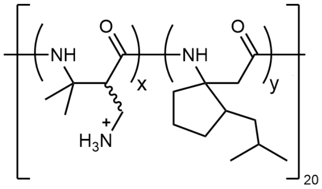	4	6	23.27	22.23	25.84
		5	5	34.55	21.61	21.31
		6	4	41.69	18.43	19.57
		7	3	63.93	16.39	18.14
		8	2	90.92	14.64	16.81
		9	1	127.88	13.62	20.18

Supplementary Table 10. DM/$\beta^{3,3}$-subunit structures. The units for all properties are (μg mL^{-1}). We default n to 20 for prediction.

Polymer	Structure	x	y	HC_{10}	$MIC_{S. aureus}$	$MIC_{E. coli}$
Polymer_1	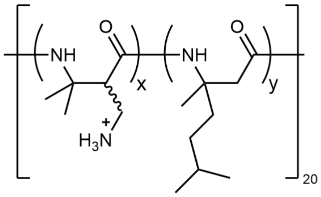	4	6	80.91	23.09	37.14
		5	5	120.95	19.74	24.25
		6	4	134.08	16.43	17.42
		7	3	173.33	14.15	15.25
		8	2	193.68	12.54	15.24
		9	1	237.55	11.27	19.92
Polymer_2	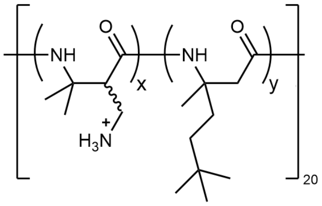	4	6	384.55	28.19	28.02
		5	5	475.43	24.64	21.01
		6	4	494.00	20.86	17.56
		7	3	583.14	18.59	15.30
		8	2	634.74	16.85	14.58
		9	1	721.03	15.52	17.45
Polymer_3	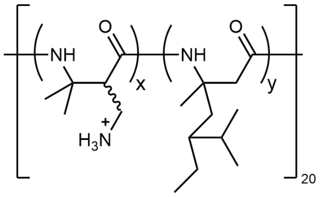	4	6	30.86	23.39	54.78
		5	5	44.66	20.64	39.99
		6	4	60.29	17.52	33.5
		7	3	92.41	15.38	27.26
		8	2	125.08	13.69	23.20
		9	1	171.97	12.29	24.95
Polymer_4	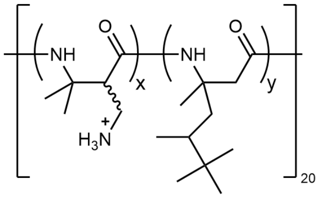	4	6	39.94	24.54	28.55
		5	5	57.11	21.73	21.83
		6	4	73.00	18.62	19.62
		7	3	101.71	16.43	17.74
		8	2	127.66	14.80	15.81
		9	1	152.35	13.48	17.26

Supplementary Table 11. DM/$\beta^{2,3}$-subunit structures. The units for all properties are (µg mL^{-1}). We default n to 20 for prediction.

Polymer	Structure	x	y	HC_{10}	$MIC_{S.\ aureus}$	$MIC_{E.\ coli}$
Polymer_1	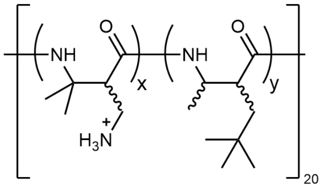	4	6	35.68	27.05	40.40
		5	5	54.88	23.45	28.87
		6	4	75.93	20.05	21.73
		7	3	112.38	17.55	18.89
		8	2	146.86	15.76	17.36
		9	1	185.62	14.07	19.37
Polymer_2	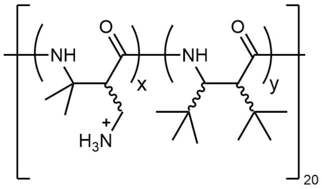	4	6	61.72	27.66	43.99
		5	5	79.71	25.19	36.97
		6	4	90.78	22.50	31.72
		7	3	112.78	20.80	28.24
		8	2	129.31	19.65	24.54
		9	1	154.56	18.35	24.72
Polymer_3	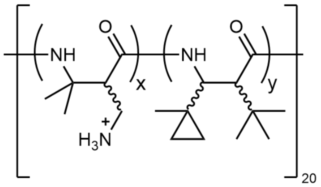	4	6	86.64	24.65	28.54
		5	5	107.09	22.80	25.71
		6	4	116.35	20.33	23.99
		7	3	146.58	18.57	22.40
		8	2	166.26	16.95	20.78
		9	1	193.41	15.82	23.39
Polymer_4	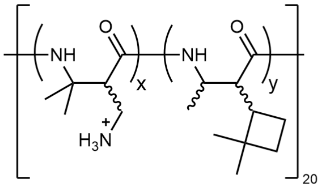	4	6	16.81	18.62	38.62
		5	5	26.31	16.50	39.98
		6	4	35.59	14.33	25.18
		7	3	54.17	12.80	22.10
		8	2	75.58	11.45	21.63
		9	1	100.80	10.74	24.05
Polymer_5	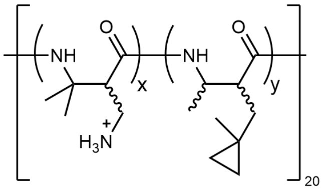	4	6	72.77	19.66	37.29
		5	5	104.40	17.93	28.32
		6	4	121.61	15.96	25.03
		7	3	169.76	14.37	23.18
		8	2	216.84	13.08	22.22
		9	1	290.15	12.48	24.42

Supplementary Table 12. DM/$\beta^{2,2,3,3}$-subunit structures. The units for all properties are (µg mL^{-1}). We default n to 20 for prediction.

Polymer	Structure	x	y	HC_{10}	$MIC_{S.\ aureus}$	$MIC_{E.\ coli}$
Polymer_1	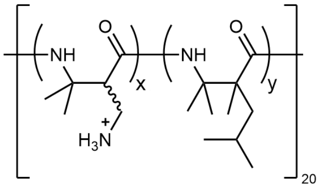	4	6	67.59	15.66	27.22
		5	5	77.90	14.58	21.10
		6	4	94.16	12.86	19.58
		7	3	116.48	11.67	19.21
		8	2	147.87	10.83	18.22
		9	1	195.12	10.29	22.29
Polymer_2	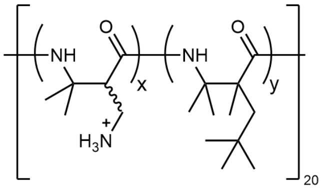	4	6	142.15	21.58	22.91
		5	5	162.52	19.84	18.30
		6	4	196.28	17.88	16.51
		7	3	253.56	16.55	15.40
		8	2	344.82	15.46	14.30
		9	1	478.64	14.32	15.56
Polymer_3	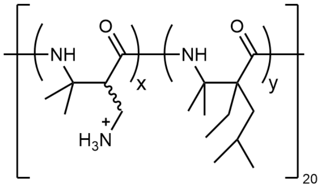	4	6	83.18	17.88	27.17
		5	5	91.48	16.01	22.00
		6	4	108.18	13.84	20.75
		7	3	132.86	12.17	20.35
		8	2	158.62	10.99	19.43
		9	1	202.37	10.12	23.65
Polymer_4	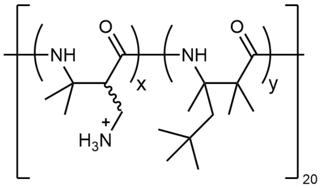	4	6	70.67	19.64	22.83
		5	5	79.61	17.83	19.20
		6	4	92.06	15.73	17.97
		7	3	108.34	14.27	16.80
		8	2	126.34	13.34	16.02
		9	1	144.44	12.48	18.78

Supplementary Table 13. MM/$\beta^{2,3}$-subunit cyclic structures. The units for all properties are (µg mL^{-1}). We default n to 20 for prediction.

Polymer	Structure	x	y	HC_{10}	$MIC_{S. aureus}$	$MIC_{E. coli}$
Polymer_1	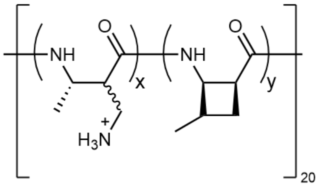	4	6	100.73	22.84	11.37
		5	5	514.60	21.26	15.12
		6	4	822.65	23.95	18.50
		7	3	1083.94	28.69	46.24
		8	2	676.55	36.10	93.03
		9	1	715.54	48.06	157.52
Polymer_2	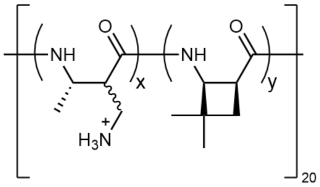	4	6	8.92	21.71	9.03
		5	5	36.78	19.71	11.05
		6	4	76.70	21.77	15.51
		7	3	237.35	25.60	24.97
		8	2	263.78	31.29	40.23
		9	1	386.62	39.89	62.67
Polymer_3	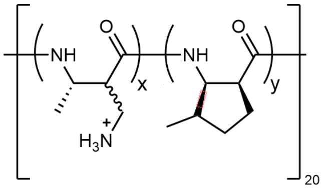	4	6	13.34	20.85	12.29
		5	5	66.87	18.23	17.40
		6	4	281.56	19.30	21.15
		7	3	1491.08	22.28	51.58
		8	2	1469.25	25.90	86.35
		9	1	1986.04	31.66	141.02
Polymer_4	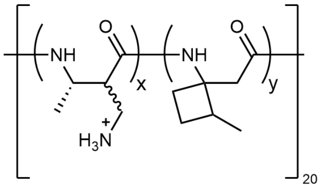	4	6	7.89	20.62	11.87
		5	5	31.11	18.12	16.06
		6	4	191.84	19.19	20.41
		7	3	674.38	23.40	49.16
		8	2	632.14	26.56	85.02
		9	1	876.06	32.79	143.05
Polymer_5	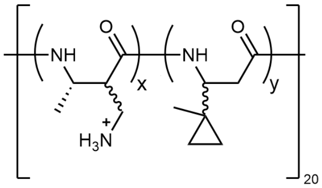	4	6	24.90	31.52	23.57
		5	5	103.46	24.91	24.86
		6	4	188.76	60.45	30.69
		7	3	446.70	35.58	62.32
		8	2	525.96	39.33	87.43
		9	1	873.60	46.32	137.55

Supplementary Table 14. DM/β^3-subunit best optimized structures. The units for all properties are (μg mL^{-1}). We default n to 20 for prediction.

Candidate	Structure	x	y	HC_{10}	$MIC_{S. aureus}$	$MIC_{E. coli}$
Candidate_1	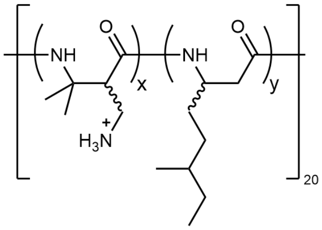	9	1	119.64	8.31	24.94
Candidate_2	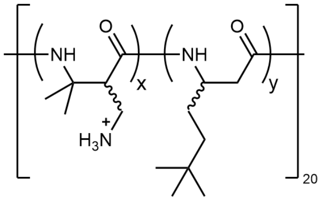	7	3	134.65	15.53	14.68
Candidate_3		8	2	167.70	13.97	15.23
Candidate_4		9	1	214.80	12.78	20.04
Candidate_5	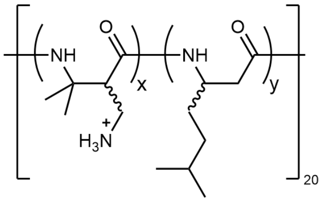	7	3	112.64	11.26	20.70
Candidate_6	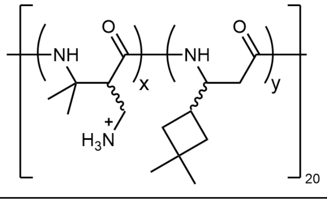	9	1	117.30	11.65	19.47
Candidate_7	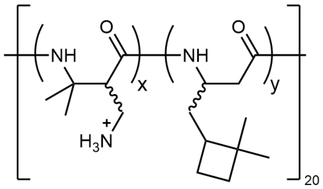	9	1	131.47	9.93	20.52
Candidate_8	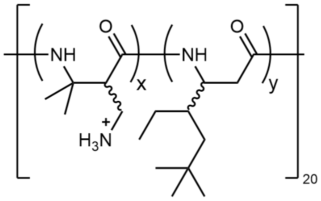	9	1	131.99	21.31	21.31
Candidate_9	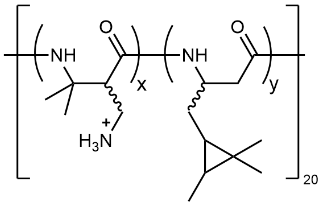	7	3	115.40	14.38	21.10
Candidate_10		8	2	154.85	13.45	20.90
Candidate_11		9	1	211.73	12.85	25.31

Supplementary Table 15. DM/β^3-subunit best optimized structures. The units for all properties are (μg mL^{-1}). We default n to 20 for prediction.

Candidate	Structure	x	y	HC_{10}	$MIC_{S.\ aureus}$	$MIC_{E.\ coli}$
Candidate_12	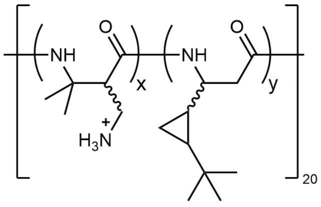	8	2	116.13	13.32	15.36
Candidate_13		9	1	164.65	12.73	18.78
Candidate_14	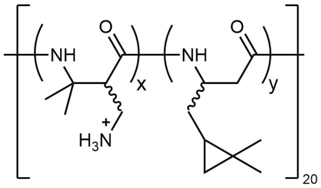	9	1	127.93	13.81	23.81
Candidate_15	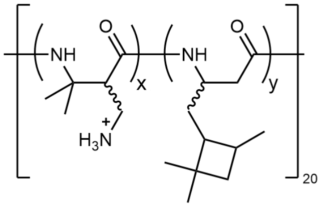	9	1	107.00	10.43	21.41
Candidate_16	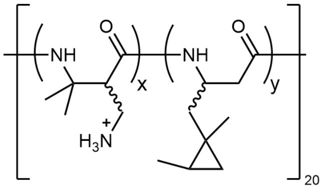	9	1	120.83	11.21	24.01
Candidate_17	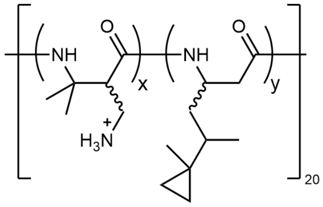	9	1	110.06	9.32	25.19
Candidate_18	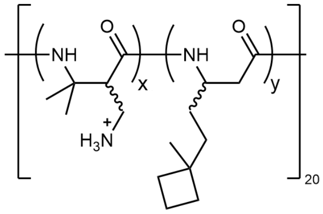	9	1	133.11	9.39	22.82
Candidate_19	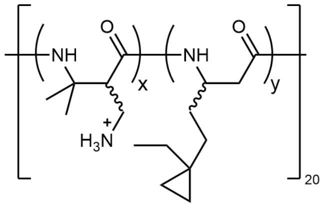	9	1	112.25	10.66	25.37

Supplementary Table 16. DM/$\beta^{3,3}$-subunit cyclic best optimized structures. The units for all properties are (μg mL^{-1}). We default n to 20 for prediction.

Candidate	Structure	x	y	HC_{10}	$MIC_{S. aureus}$	$MIC_{E. coli}$
Candidate_20	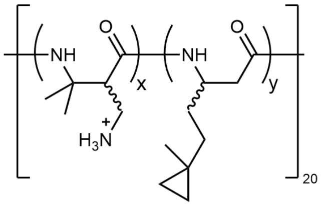	5	5	127.70	16.57	13.12
Candidate_21		6	4	153.08	14.63	12.45
Candidate_22		7	3	228.38	13.30	13.43
Candidate_23		8	2	287.91	12.09	16.25
Candidate_24		9	1	401.57	12.27	24.97
Candidate_25	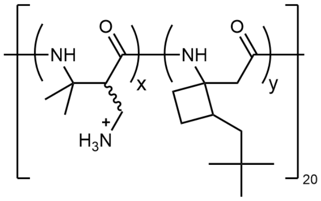	6	4	151.02	22.98	15.34
Candidate_26		7	3	196.57	20.73	13.94
Candidate_27		8	2	238.32	18.66	12.13
Candidate_28		9	1	305.03	17.22	13.19
Candidate_29	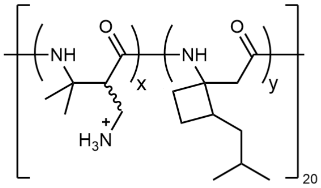	8	2	109.05	12.50	large17.09
Candidate_30		9	1	150.11	11.74	21.11
Candidate_31	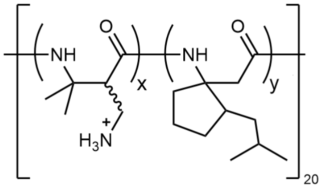	9	1	127.88	13.62	20.18

Supplementary Table 17. DM/$\beta^{3,3}$-subunit best optimized structures. The units for all properties are (μg mL^{-1}). We default n to 20 for prediction.

Candidate	Structure	x	y	HC_{10}	$MIC_{S.\ aureus}$	$MIC_{E.\ coli}$
Candidate_32	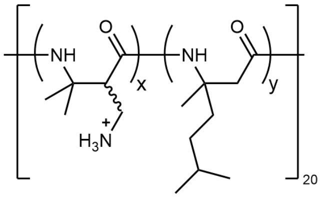	5	5	120.95	19.74	24.25
Candidate_33		6	4	134.08	16.43	17.42
Candidate_34		7	3	173.33	14.15	15.25
Candidate_35		8	2	193.68	12.54	15.24
Candidate_36		9	1	237.55	11.27	19.92
Candidate_37	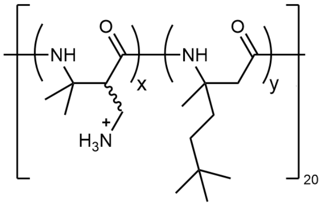	5	5	475.43	24.64	21.01
Candidate_38		6	4	494.00	20.86	17.56
Candidate_39		7	3	583.14	18.59	15.30
Candidate_40		8	2	634.74	16.85	14.58
Candidate_41		9	1	721.03	15.52	17.45
Candidate_42	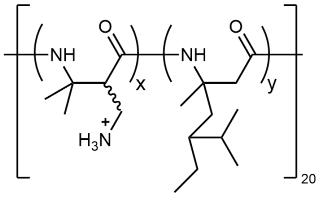	8	2	125.08	13.69	23.20
Candidate_43		9	1	171.97	12.29	24.95
Candidate_44	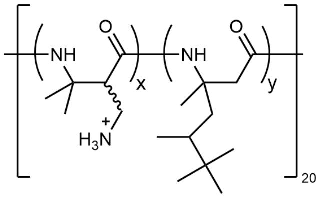	7	3	101.71	16.43	17.74
Candidate_45		8	2	127.66	14.80	15.81
Candidate_46		9	1	152.35	13.48	17.26

Supplementary Table 18. DM/$\beta^{2,3}$-subunit best optimized structures. The units for all properties are (µg mL^{-1}). We default n to 20 for prediction.

Candidate	Structure	x	y	HC_{10}	$MIC_{S. aureus}$	$MIC_{E. coli}$
Candidate_47	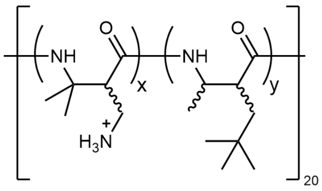	7	3	112.38	17.55	18.89
Candidate_48		8	2	146.86	15.76	17.36
Candidate_49		9	1	185.62	14.07	19.37
Candidate_50	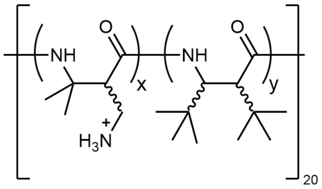	8	2	129.31	19.65	24.54
Candidate_51		9	1	154.56	18.35	24.72
Candidate_52	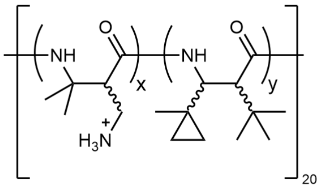	6	4	116.35	20.33	23.99
Candidate_53		7	3	146.58	18.57	22.40
Candidate_54		8	2	166.26	16.95	20.78
Candidate_55		9	1	193.41	15.82	23.39
Candidate_56	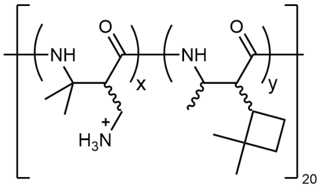	9	1	100.80	10.74	24.05
Candidate_57	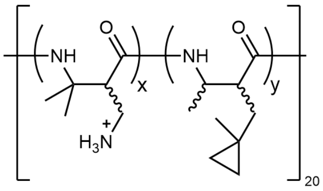	6	4	121.61	15.96	25.03
Candidate_58		7	3	169.76	14.37	23.18
Candidate_59		8	2	216.84	13.08	22.22
Candidate_60		9	1	290.15	12.48	24.42

Supplementary Table 19. DM/$\beta^{2,2,3,3}$-subunit best optimized structures. The units for all properties are (µg mL^{-1}). We default n to 20 for prediction.

Candidate	Structure	x	y	HC_{10}	$MIC_{S.\ aureus}$	$MIC_{E.\ coli}$
Candidate_61	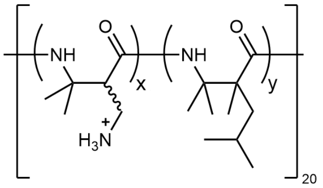	7	3	116.48	11.67	19.21
Candidate_62		8	2	147.87	10.83	18.22
Candidate_63		9	1	195.12	10.29	22.29
Candidate_64	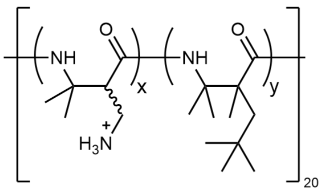	4	6	142.15	21.58	22.91
Candidate_65		5	5	162.52	19.84	18.30
Candidate_66		6	4	196.28	17.88	16.51
Candidate_67		7	3	253.56	16.55	15.40
Candidate_68		8	2	344.82	15.46	14.30
Candidate_69		9	1	478.64	14.32	15.56
Candidate_70	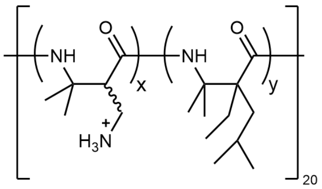	6	4	108.18	13.84	20.75
Candidate_71		7	3	132.86	12.17	20.35
Candidate_72		8	2	158.62	10.99	19.43
Candidate_73		9	1	202.37	10.12	23.65
Candidate_74	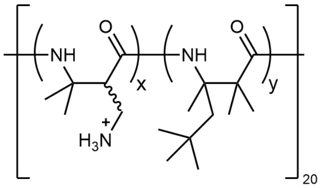	7	3	108.34	14.27	16.80
Candidate_75		8	2	126.34	13.34	16.02
Candidate_76		9	1	144.44	12.48	18.78

Supplementary Table 20. MM/subunit best optimized structures. The units for all properties are (µg mL^{-1}). We default n to 20 for prediction.

Candidate	Structure	x	y	HC_{10}	$MIC_{S.\ aureus}$	$MIC_{E.\ coli}$
Candidate_77	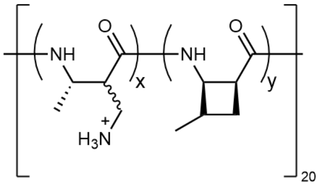	4	6	100.73	22.84	11.37
Candidate_78		5	5	514.60	21.26	15.12
Candidate_79		6	4	822.65	23.95	18.50
Candidate_80	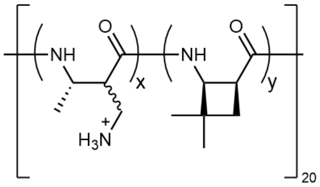	7	3	237.35	25.60	24.97
Candidate_81	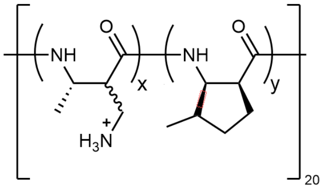	6	4	281.56	19.30	21.15
Candidate_82	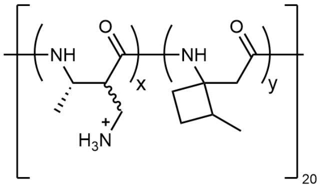	6	4	191.84	19.19	20.41
Candidate_83	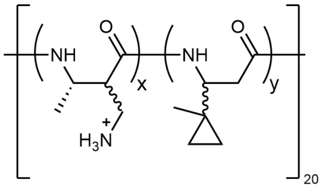	5	5	103.46	24.91	24.86

Supplementary References

[1] Wu, Y., Zhang, D., Ma, P., Zhou, R., Hua, L., Liu, R.: Lithium hexamethyldisilazide initiated superfast ring opening polymerization of alpha-amino acid n-carboxyanhydrides. Nat. Commun. 9(1), 1-10 (2018)

[2] Kuroda, K., DeGrado, W.F.: Amphiphilic polymethacrylate derivatives as antimicrobial agents. J. Am. Chem. Soc. 127(12), 4128-4129 (2005)

[3] Sovadinova, I., Palermo, E.F., Urban, M., Mpiga, P., Caputo, G.A., Kuroda, K.: Activity and mechanism of antimicrobial peptide-mimetic amphiphilic polymethacrylate derivatives. Polymers 3(3), 1512-1532 (2011)

[4] Kuroda, K., Caputo, G.A., DeGrado, W.F.: The role of hydrophobicity in the antimicrobial and hemolytic activities of polymethacrylate derivatives. Chemistry-A European Journal 15(5), 1123-1133 (2009)

[5] Locock, K.E., Michl, T.D., Stevens, N., Hayball, J.D., Vasilev, K., Postma, A., Griesser, H.J., Meagher, L., Haeussler, M.: Antimicrobial polymethacrylates synthesized as mimics of tryptophan-rich cationic peptides. ACS Macro Lett. 3(4), 319-323 (2014)

[6] Palermo, E.F., Sovadinova, I., Kuroda, K.: Structural determinants of antimicrobial activity and biocompatibility in membrane-disrupting methacrylamide random copolymers. Biomacromolecules 10(11), 3098-3107 (2009)

[7] Ilker, M.F., N¨usslein, K., Tew, G.N., Coughlin, E.B.: Tuning the hemolytic and antibacterial activities of amphiphilic polynorbornene derivatives. J. Am. Chem. Soc. 126(48), 15870-15875 (2004)

[8] Sambhy, V., Peterson, B.R., Sen, A.: Antibacterial and hemolytic activities of pyridinium polymers as a function of the spatial relationship between the positive charge and the pendant alkyl tail. Angew. Chem. 120(7), 1270-1274 (2008)

[9] Oda, Y., Kanaoka, S., Sato, T., Aoshima, S., Kuroda, K.: Block versus random am-

phiphilic copolymers as antibacterial agents. Biomacromolecules 12(10), 3581-3591 (2011)
