# Supplementary material for: AI-guided few-shot inverse design of HDP-mimicking polymers against drug-resistant bacteria
Source: Nat Commun. 2024 Jul 26;15:6288. doi: 10.1038/s41467-024-50533-4 (PMC11282099; doi:10.1038/s41467-024-50533-4)
Supplement: Supplementary file 3 — Description of Additional Supplementary Files [file 41467_2024_50533_MOESM3_ESM.docx]

**Description of Additional Supplementary Files**

**Supplementary Data 1:** Raw data for collected β-amino acid polymers with corresponding value of MICS. aureus, MICE. coli, HC10.

**Supplementary Data 2:** SMIELS sequence defined for β-amino acid polymers.

**Supplementary Data 3:** SMILES sequence for β-amino acids and natural α--amino acid used in graph grammar distillation.

**Supplementary Data 4:** SMILES sequence of the scaffolds to pre-train the generative model.

**Supplementary Data 5:** SMILES sequence of the decorators to pre-train the generative model.

**Supplementary Data 6:** Raw data for other types of antibacterial polymers.
