# Supplementary material for: AI-guided few-shot inverse design of HDP-mimicking polymers against drug-resistant bacteria
Source: Nat Commun. 2024 Jul 26;15:6288. doi: 10.1038/s41467-024-50533-4 (PMC11282099; doi:10.1038/s41467-024-50533-4)
Supplement: Supplementary file 10 — Reporting Summary [file 41467_2024_50533_MOESM10_ESM.pdf]

Corresponding author(s): Runhui Liu, Yang Tang

Last updated by author(s): May 20, 2024

# Reporting Summary

Nature Portfolio wishes to improve the reproducibility of the work that we publish. This form provides structure for consistency and transparency in reporting. For further information on Nature Portfolio policies, see our Editorial Policies and the Editorial Policy Checklist.

## Statistics

For all statistical analyses, confirm that the following items are present in the figure legend, table legend, main text, or Methods section.

| n/a | Confirmed | |
|---|---|---|
| ☐ | ☒ | The exact sample size (*n*) for each experimental group/condition, given as a discrete number and unit of measurement |
| ☐ | ☒ | A statement on whether measurements were taken from distinct samples or whether the same sample was measured repeatedly |
| ☐ | ☒ | The statistical test(s) used AND whether they are one- or two-sided<br>*Only common tests should be described solely by name; describe more complex techniques in the Methods section.* |
| ☒ | ☐ | A description of all covariates tested |
| ☒ | ☐ | A description of any assumptions or corrections, such as tests of normality and adjustment for multiple comparisons |
| ☐ | ☒ | A full description of the statistical parameters including central tendency (e.g. means) or other basic estimates (e.g. regression coefficient) AND variation (e.g. standard deviation) or associated estimates of uncertainty (e.g. confidence intervals) |
| ☐ | ☒ | For null hypothesis testing, the test statistic (e.g. *F*, *t*, *r*) with confidence intervals, effect sizes, degrees of freedom and *P* value noted<br>*Give P values as exact values whenever suitable.* |
| ☒ | ☐ | For Bayesian analysis, information on the choice of priors and Markov chain Monte Carlo settings |
| ☐ | ☒ | For hierarchical and complex designs, identification of the appropriate level for tests and full reporting of outcomes |
| ☒ | ☐ | Estimates of effect sizes (e.g. Cohen's *d*, Pearson's *r*), indicating how they were calculated |

*Our web collection on statistics for biologists contains articles on many of the points above.*

## Software and code

Policy information about availability of computer code

| | |
|---|---|
| Data collection | Python (version:3.7.10) was used to curate the dataset. The code is available at https://github.com/TianyuWu813/polymer_generation and https://github.com/TianyuWu813/polymer_prediction.<br>We used AVANCE III 400 spectrometer (400 MHz) with TopSpin software (version:3.1), Ascend 600 spectrometer (600 MHz) with TopSpin software (version:4.0.3), Hitachi S-4800 Field Emission Scanning Electron Microscope operated with FE-PC SEM software (version: 3.18), Leica TCS SP8 with LAS X imaging software (version: 1.0.0.) |
| Data analysis | Python (version:3.7.10) and Pytorch (version: 1.2.0)were used to implement the polymer predictive and generative models. The codes are available at https://github.com/TianyuWu813/polymer_generation and https://github.com/TianyuWu813/polymer_prediction. Mordred (version: 1.2.0) as well as in-house python codes were used to calculate descriptors. MLforCOE (version: 0.0.0) was used to run the descriptors downselection.<br>Origin (version: 9.65.169), MestReNova (version: 11.0.18998), Breeze 2 (version: 2154-C). |

For manuscripts utilizing custom algorithms or software that are central to the research but not yet described in published literature, software must be made available to editors and reviewers. We strongly encourage code deposition in a community repository (e.g. GitHub). See the Nature Portfolio guidelines for submitting code & software for further information.

## Data

Policy information about availability of data

All manuscripts must include a data availability statement. This statement should provide the following information, where applicable:

- Accession codes, unique identifiers, or web links for publicly available datasets
- A description of any restrictions on data availability
- For clinical datasets or third party data, please ensure that the statement adheres to our policy

All collected raw data of β-amino acid polymers, α-amino acid polymers, polymethacrylates, polymethacrylamides and other categories are available on GitHub: https://github.com/TianyuWu813/polymer_prediction for polymer property prediction . All raw data to train the generative model are available on https://github.com/TianyuWu813/polymer_generation. The source data for all figures and tables in the manuscript and in the Supplementary Information are provided with this paper. Source data are provided with this paper.

## Research involving human participants, their data, or biological material

Policy information about studies with human participants or human data. See also policy information about sex, gender (identity/presentation), and sexual orientation and race, ethnicity and racism.

| | |
|---|---|
| Reporting on sex and gender | The authors declare no sex or gender data was involved in this manuscript. |
| Reporting on race, ethnicity, or other socially relevant groupings | The authors declare no race, ethnicity, or other socially relevant groupings data was involved in this manuscript. |
| Population characteristics | The authors declare no population characteristics was involved in this manuscript. |
| Recruitment | The authors declare no recruitment data was involved in this manuscript. |
| Ethics oversight | The authors declare no human participants or human data was involved in this manuscript. |

Note that full information on the approval of the study protocol must also be provided in the manuscript.

# Field-specific reporting

Please select the one below that is the best fit for your research. If you are not sure, read the appropriate sections before making your selection.

☒ Life sciences ☐ Behavioural & social sciences ☐ Ecological, evolutionary & environmental sciences

For a reference copy of the document with all sections, see nature.com/documents/nr-reporting-summary-flat.pdf

# Life sciences study design

All studies must disclose on these points even when the disclosure is negative.

| | |
|---|---|
| Sample size | For AI design, the sample size is mainly decided by manually collected experimental data or previously reported data, totally n=86. These data is randomly split into trained/tested data (8/2) in each phase of polymer property prediction. |
| Data exclusions | The authors declare no data exclusion. |
| Replication | For the replication of AI design part, In the descriptor downselection phase, a 15-fold cross validation is applied. In the polymer property prediction phase, independent datasets consisting of randomly split trained/tested data (8/2) were used to train the predictive model for polymer properties with 10 independent runs with different random seeds. In the phase of polymer generation, one independent run is applied.<br>At least three independent measurements were taken for the hemolysis and cytotoxicity of polymers against different cells, getting similar results. The SEM sample was prepared once, and at least 50 fungal cells were observed individually in the sample, showing successful results similar to the representative SEM images. |
| Randomization | In the descriptor downselection phase, a 15-fold cross validation is applied. In the polymer property prediction phase, independent datasets consisting of randomly split trained/tested data (8/2) were used to train the predictive model for polymer properties with 10 independent runs with different random seeds. Also independent datasets consisting of randomly selected AI-generated/screened data were used for estimating the mean and the variance of attributes. |
| Blinding | Blinding was not relevant, as this study provides an AI-guided framework for accelerated antimicrobial discovery and the evaluation of AI-generated antimicrobial mimicking polymers. |

# Reporting for specific materials, systems and methods

We require information from authors about some types of materials, experimental systems and methods used in many studies. Here, indicate whether each material, system or method listed is relevant to your study. If you are not sure if a list item applies to your research, read the appropriate section before selecting a response.

## Materials & experimental systems

| n/a | Involved in the study |
| --- | --- |
| x | Antibodies |
| | x Eukaryotic cell lines |
| x | Palaeontology and archaeology |
| x | Animals and other organisms |
| x | Clinical data |
| x | Dual use research of concern |
| x | Plants |

## Methods

| n/a | Involved in the study |
| --- | --- |
| x | ChIP-seq |
| x | Flow cytometry |
| x | MRI-based neuroimaging |

# Eukaryotic cell lines

Policy information about cell lines and Sex and Gender in Research

| | |
| --- | --- |
| Cell line source(s) | Human umbilical vein endothelial cell line (HUVEC) and the African green monkey kidney fibroblasts (COS-7) cells were obtained from the Cell Bank of the Chinese Academy of Sciences (Shanghai, China).<br>All sourced blood for hemolysis assays were donated by the Shanghai RuiJin Rehabilitation Hospital before the blood is disposed as scheduled and no recruitment information was supplied to the researchers of this project as per the agreement with University of East China University of Science and Technology Human Ethics Approval, therefore recruitment information are unknown. |
| Authentication | Human umbilical vein endothelial cell line (HUVEC) and the African green monkey kidney fibroblasts (COS-7) cells were not authenticated by us repeatedly. |
| Mycoplasma contamination | No mycoplasma contamination. |
| Commonly misidentified lines<br>(See ICLAC register) | No commonly misidentified lines. |

# Plants

| | |
| --- | --- |
| Seed stocks | We declare no plants were used in this manuscript. |
| Novel plant genotypes | We declare no plants were used in this manuscript. |
| Authentication | We declare no plants were used in this manuscript. |
